# Discovery of Novel Imidazopyridine GSK-3β Inhibitors Supported by Computational Approaches

**DOI:** 10.3390/molecules25092163

**Published:** 2020-05-05

**Authors:** Rosa Buonfiglio, Federica Prati, Martina Bischetti, Claudia Cavarischia, Guido Furlotti, Rosella Ombrato

**Affiliations:** Angelini Pharma S.p.A., Viale Amelia, 70, 00181 Rome, Italy; rosa.buonfiglio@angelinipharma.com (R.B.); federica.prati@angelinipharma.com (F.P.); martina.bischetti@angelinipharma.com (M.B.); claudia.cavarischia@angelinipharma.com (C.C.); guido.furlotti@angelinipharma.com (G.F.)

**Keywords:** glycogen synthase kinase-3β (GSK-3β), 1H-indazole-3-carboxamide core (INDZ), imidazo[1,5-a]pyridine-1-carboxamide core (IMID 1), imidazo[1,5-a]pyridine-3-carboxamide core (IMID 2), CNS permeability, molecular docking, molecular dynamics (MD), quantum mechanics (QM), X-ray crystallography, NMR characterization

## Abstract

The interest of research groups and pharmaceutical companies to discover novel GSK-3β inhibitors has increased over the years considering the involvement of this enzyme in many pathophysiological processes and diseases. Along this line, we recently reported on 1H-indazole-3-carboxamide (INDZ) derivatives **1**–**6**, showing good GSK-3β inhibition activity. However, they suffered from generally poor central nervous system (CNS) permeability. Here, we describe the design, synthesis, and in vitro characterization of novel imidazo[1,5-a]pyridine-1-carboxamide (IMID 1) and imidazo[1,5-a]pyridine-3-carboxamide (IMID 2) compounds (**7**–**18**) to overcome such liability. In detail, structure-based approaches and fine-tuning of physicochemical properties guided the design of derivatives **7**–**18** resulting in ameliorated absorption, distribution, metabolism, and excretion (ADME) properties. A crystal structure of **16** in complex with GSK-3β enzyme (PDB entry 6Y9S) confirmed the in silico models. Despite the nanomolar inhibition activity, the new core compounds showed a reduction in potency with respect to INDZ derivatives **1**–**6**. In this context, Molecular Dynamics (MD) and Quantum Mechanics (QM) based approaches along with NMR investigation helped to rationalize the observed structure activity relationship (SAR). With these findings, the key role of the acidic hydrogen of the central core for a tight interaction within the ATP pocket of the enzyme reflecting in good GSK-3β affinity was demonstrated.

## 1. Introduction

Glycogen synthase kinase-3 (GSK-3) is a ubiquitous, multifunctional and constitutively active serine/threonine kinase, involved in numerous pathways and over one hundred different putative substrates [1].

In humans, GSK-3 enzyme is encoded by two independent genes that regulate the expression of two diverse isoforms, namely GSK-3α and GSK-3β, with high sequence homology (~98%) in their kinase domain [2] and substantial functional overlap [3,4,5].

GSK-3β is a significantly networked enzyme, which regulates a plethora of physiological functions in peripheral tissues and the central nervous system (CNS), spanning from metabolism and cell cycle regulation to brain development. Accordingly, balanced GSK-3β regulation and signaling is key to human physiology but often compromised in diverse disease conditions such as diabetes, cancers, and brain disorders [6,7]. As for CNS-related pathologies, aberrant GSK-3β activity has been reported in neurological or neurodevelopmental diseases, such as neurodegenerative disorders (Alzheimer’s and Parkinson’s diseases), bipolar disorder, depression, and Fragile X syndrome [8,9,10,11]. Moreover, clinical, genetic, and pharmacological investigations suggested that GSK-3β inhibition may attenuate signaling dysfunction in such diseases [12,13]. Indeed, the role of GSK-3β in mood disorder was highlighted by studies on the mechanism of actions of the established treatments lithium and valproate, which were both found to inhibit GSK-3β [14,15]. 

Based on this evidence, intensive efforts have been made in the search for novel GSK-3β inhibitors as innovative therapeutic agents, which, however, have not been translated into clinical practice yet. Therefore, GSK-3β inhibitors are still needed.

**Figure 1:**
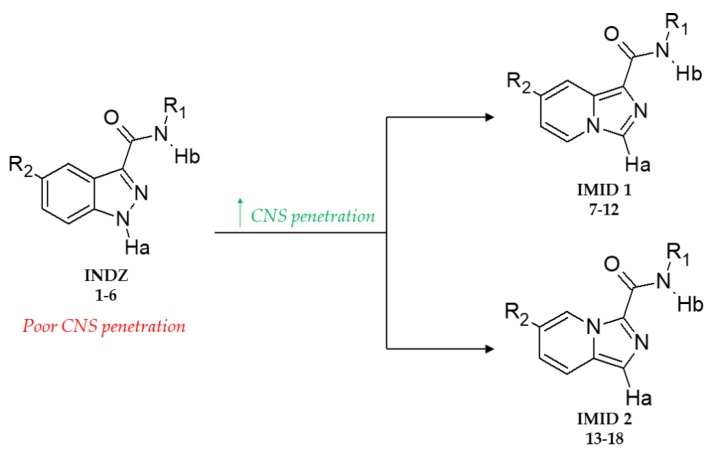
Exploration of IMID 1 and IMID 2 derivatives **7**–**18** starting from previous INDZ **1**–**6** to improve CNS penetration.

On these premises, a discovery program started at Angelini Pharma with a computer-aided protocol based on structure-based virtual screening and similarity search, which led to the identification of the 1H-indazole-3-carboxamide moiety (INDZ) as a new scaffold for GSK-3β inhibition [16]. Subsequent optimization campaigns followed to optimize GSK-3β inhibition potency, as well as ADMET properties, and resulted in INDZ derivatives **1**–**6** [17,18,19] (Figure 1 and Table 1). 

Despite few derivatives showing promising in vitro profiles, enhanced blood brain barrier (BBB) penetration was still desirable to hinder CNS disorders. Indeed, the BBB is a dynamic and functional structure which poses a vast challenge in the development of drugs acting on the CNS [20]. The major route of drug permeation through the BBB is passive diffusion, which can be boosted by fine-tuning several physicochemical properties. A variety of approaches has been put forward to guide the design of BBB penetrant compounds based upon these features [21,22,23]. 

On these bases, starting from the INDZ core, compound physicochemical property evaluation and computer-aided methods were applied to support a scaffold hopping approach leading to the identification of novel imidazo[1,5-a]pyridine-1-carboxamide (IMID 1) and imidazo[1,5-a]pyridine-3-carboxamide (IMID 2) derivatives **7**–**18** (Figure 1 and Table 1) as putative GSK-3β inhibitors with improved in vitro CNS permeability. Specifically, structure-based approaches were applied to predict ligand–protein interactions, which were confirmed by the high-resolution co-crystal structure of compound **16** with human GSK-3β enzyme (PDB entry 6Y9S). Additionally, computational investigations based on Molecular Dynamics (MD) simulations and Quantum Mechanics (QM) helped to retrospectively rationalize the structure activity relationships (SARs) of the in vitro tested compounds. NMR-based studies were also carried out to further substantiate the observed experimental data.

## 2. Results and Discussion

### 2.1. Chemistry

**Scheme 1:**
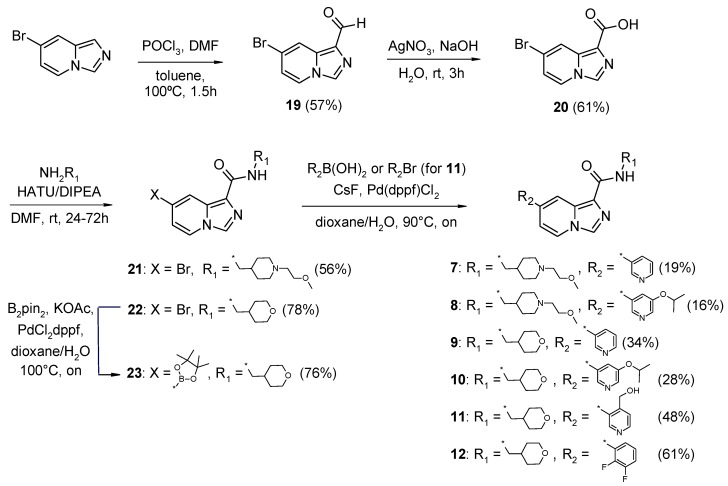
Synthesis of imidazo[1,5-*a*]pyridine-1-carboxamide derivatives **7**–**12**.

**Scheme 2:**
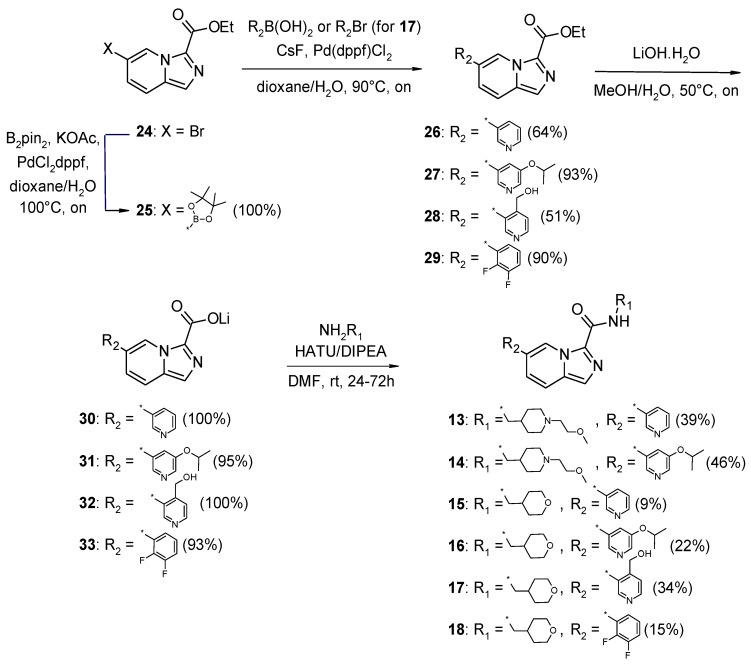
Synthesis of imidazo[1,5-*a*]pyridine-3-carboxamide derivatives **13**–**18**.

INDZ derivatives **1**–**6** were synthesized as previously described [17,18,19], whereas imidazo[1,5-a]pyridines **7**–**18** were prepared according to the linear synthetic routes depicted in Scheme 1 and Scheme 2. Specifically, IMID 1 derivatives **7**–**10** and **12** were obtained in a four-step synthesis starting from the commercially available 7-bromoimidazo[1,5-*a*]pyridine (Scheme 1). 7-bromoimidazo[1,5-*a*]pyridine underwent Vilsmeier–Haack reaction in the presence of DMF and phosphorus oxychloride to provide the corresponding aldehyde (**19**). Subsequent silver nitrate-mediated oxidation led to the carboxylic acid **20**, which was then coupled to 1-[1-(2-methoxyethyl)piperidin-4-yl]methanamine and 1-(oxan-4-yl)methanamine under standard carboxylic acid-amine coupling reaction to afford amides **21** and **22**, respectively. Final Suzuki coupling of **21** and **22** with the desired boronic acids gave the target compounds **7**–**10** and **12**. The alkoxy-substituted pyridinyl derivative **11** was synthesized by the additional conversion of the 7-bromo-*N*-[(oxan-4-yl)methyl]imidazo[1,5-a]pyridine-1-carboxamide **22** to the corresponding boronic ester **23** by treatment with bis(pinacolato)diboron and subsequent Suzuki coupling with (3-bromopyridin-4-yl)methanol (Scheme 1). 

IMID 2 derivatives **13**–**16** and **18** (Scheme 2) were prepared starting from the commercially available 6-bromoimidazo[1,5-*a*]pyridine-3-carboxylate **24**. Bromide **24** underwent standard Suzuki coupling reaction with the desired boronic acids to give the corresponding intermediates **26**, **27**, and **29**. Subsequent ester hydrolysis with lithium hydroxide provided lithium carboxylate salts **30**, **31**, and **33**, which were finally coupled to 1-[1-(2-methoxyethyl)piperidin-4-yl]methanamine and 1-(oxan-4-yl)methanamine to provide the target compounds. Similarly to compound **11**, synthesis of the alkoxy-substituted pyridinyl derivative **17** required the additional conversion of bromide **24** to the corresponding boronic ester **25**, which then entered the same three-step synthetic route as described above (Scheme 2).

### 2.2. Derivatives ***1**–**6*** and Rationale for New Design

**Figure 2:**
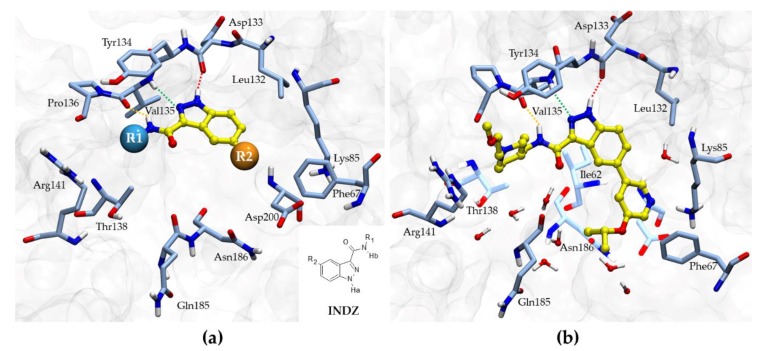
(**a**) Generic binding mode of the INDZ core in the ATP binding domain of GSK-3β enzyme extracted from crystallographic studies discussed elsewhere [16,17,19]. The 2D structure of the INDZ core with explicit Ha and Hb atoms is also showed; (**b**) X-ray co-crystal structure of inhibitor **2** in complex with GSK-3β enzyme (PDB entry 6Y9R). The deep, central and outer H-bond interactions are represented as red, green, and orange dashed lines, respectively. The most relevant residues of the ATP binding site are showed in light blue (for clarity, light transparency is used for some amino acids). Relevant water molecules around inhibitor **2** are also displayed. Both figures were prepared with VMD 1.9.4 [24].

The INDZ core was previously identified as an attractive scaffold for the development of novel GSK-3β inhibitors [16,17,18,19]. Through in silico and crystallographic studies [16,17,19], it was found to bind in the ATP binding domain of GSK-3β enzyme between the N- and C-terminal lobes (Figure 2a). The INDZ moiety is located at the adenine binding site and contacts the hinge residues via three hydrogen bonds (H-bonds) referred to as: 1) “deep” engaging the hydrogen (Ha) at position N-1 of the core with Asp133 carbonyl group, 2) “central” involving the N-2 of the core and Val135 N-H group, and 3) “outer” between the hydrogen (Hb) of the carboxamide group and Val135 carbonyl (the three interactions are represented in Figure 2a as red, green, and orange dashed lines, respectively). In addition, R_1_ and R_2_ substituents appended to the central core are oriented towards Arg141 in the external solvent accessible part of the binding pocket and the inner cavity in proximity of Lys85, respectively.

Compounds **1**–**6**, published ahead as potent GSK-3β inhibitors [17,18,19], are reported herein as representative derivatives of the INDZ series and served as starting point for an optimization exercise towards the development of novel compounds with improved CNS penetration. Compounds **1**–**6** showed outstanding GSK-3β inhibitory activity in the nanomolar range, with derivatives **2** and **4** being the most potent of the set (IC_50_ = 4 and 9 nM, respectively). Interestingly, both compounds **2** and **4** carry an isopropoxypyridinyl group as R_2_ substituent, resulting from 3 to 5-fold more potent than the corresponding pyridinyl analogues **1** and **3**, respectively. The high-resolution X-ray crystal structure of **2** in complex with GSK-3β (2.08 Å, PDB entry 6Y9R) supported the expected binding mode (Figure 2b). Here, the piperidine chain was solvent exposed towards Arg141, the INDZ was anchored to the hinge domain via the tridentate H-bond interaction, the pyridine formed a H-bond with the catalytic Lys85, and the isopropoxyl group faced the ribose binding site. In proximity of the ligand, a network of hydration sites was found involving residues like Ile62, Leu132, Thr138, Gln185, Asn186, and Asp200. Interestingly, MD simulations (see below) highlighted that the isopropoxyl group of **2** might be also involved in additional water-mediated interactions, justifying the boost in potency with respect to the pyridinyl analogue **1**. Replacement of the R_2_ pyridinyl group of **3** with a (hydroxymethyl)pyridinyl (**5**) or a di-F-phenyl (**6**) also retained double-digit nanomolar inhibition activity. Finally, when comparing the enzymatic potency of the pyridinyl compound **1** against **3** or the isopropoxypyridinyl derivative **2** against **4**, it appeared that the two matching pairs resulted almost equipotent, suggesting that the R_1_ substituents do not significantly impact on the GSK-3β inhibitory activity. This is in line with the observed INDZ binding mode, where the R_1_ group is solvent oriented.

Alongside enzymatic potency evaluation, preliminary brain permeability needs also to be assessed for compounds to be successfully advanced in the CNS therapeutic area. Indeed, BBB penetration is still the bottleneck in brain drug development and is the single most important factor limiting the future growth of neurotherapeutics. The MDCK-MDR1 cell line is considered a good surrogate of the in vivo BBB model. Therefore, we report herein on compounds **2**, **3**, **4**, and **6** that, being the most potent derivatives of the dataset, were selected as INDZ representatives for experimental testing in an in vitro MDCK-MDR1 permeability assay (Table 1) [25]. The permeability coefficients (P_app_) were calculated by measuring the transport of the compounds from the apical (A) to the basolateral (B) direction (Table 1 and Equation (1)). Efflux Ratio (ER) was also measured (see Equation (2)) to quantify efflux transport across a cell monolayer expressing P-glycoproteins (P-gp), by assessing transport in both directions (A-B and B-A) [26]. 

Interestingly, compounds **3** and **6** showed moderate permeability (P_app_ = 15.8 × 10^−6^ and 13.3 × 10^−6^ cm/s, respectively), whereas compounds **2** and **4** resulted only poorly permeable (P_app_ < 1.5 × 10^−6^ cm/s). Moreover, high ER values were obtained for derivatives **2** and **4** and lower ratios for compounds **3** and **6**. The observed experimental data could be rationalized by analysis of the physicochemical profile. Indeed, several properties have consistently been found to be relevant for optimal brain exposure, such as lipophilicity, expressed by the calculated logarithm of the octanol/water partition coefficient (cLogP) or calculated logarithm of the octanol/water partition coefficient at physiological pH 7.4 (cLogD), topological polar surface area (TPSA), ionization state (pK_a_), number of H-bond donors (HBD) and acceptors (HBA), rotatable bond (RB) count, and molecular weight (MW). Up to now, many published works endeavor to provide guidance in terms of physicochemical property range for the design of CNS penetrant molecules [27]. Many of these studies have focused on the profile of marketed CNS drugs. As an example, the median values derived from an analysis of marketed CNS drugs conducted at Ely Lilly define a CNS drug profile as: cLogP = 3.1, cLogD = 1.9, TPSA = 40.5 Å, most basic pK_a_ = 7.9, HBD = 1, HBA = 4, RB count = 4, and MW = 300. Obviously, these numbers are median values, and there are many marketed drugs with properties at either end of the range [27]. Overall, CNS drug property studies, despite providing different values, came to the common conclusion that CNS drugs are generally smaller, more lipophilic, with fewer HBDs, and lower TPSA compared with oral non-CNS drugs [21]. At this regard, cLogD (pH = 7.4), TPSA, the most basic pK_a_, HBD, and MW of compounds **1**–**6** were calculated here with ACD/Percepta 2017.2, for permeability estimation. Overall, the higher P_app_ values of derivative **3** and **6** with respect to **2** and **4** could be ascribed to a more desirable CNS profile, characterized by higher lipophilicity (in particular **6**), reduced H-bond potential (TPSA), and lower molecular size (Table 1). Moreover, derivatives **2** and **4** showed higher ER compared to **3** and **6**, a result in agreement with the observation that increasing the numbers of HBA and MW would confer greater likelihood of P-gp efflux [28,29]. 

In the context of CNS drug design, the HBD count, an important H-bond capacity descriptor, is a critical CNS differentiating parameter [21,22,23]. Indeed, it has been reported that increased HBD count is associated with lower passive solubility and higher risk of interaction with efflux transporters. Particularly, compounds with HBD > 2 are highly unlikely to be brain penetrant. Therefore, we conceived a new scaffold hopping design strategy to reduce the HBD count of the INDZ core (HBD = 2) and boost the BBB penetration of the current series. In particular, we replaced the indazole N-Ha with a C-Ha group and shuffled the ring nitrogen atoms resulting in the IMID 1 and IMID 2 cores (Table 1). These modifications were intended to reduce the acidity of the Ha atom so to enhance permeability, while retaining the tridentate H-bond interaction with the hinge residues and enzymatic potency. 

We expected SAR to be reasonably transferrable from the INDZ to the IMID 1 and IMID 2 series; thus, new derivatives **7**–**18** were obtained by decorating the IMID 1 and IMID 2 cores with the same R_1_ and R_2_ substituents discussed above for INDZs **1**–**6**.

### 2.3. In Silico Investigation of Novel ***7**–**18*** Derivatives

Taking advantage of the crystal structure of **2** in complex with GSK-3β, we ran Glide Standard Precision (SP) ligand docking [30,31] and Molecular Dynamics (MD) simulations [32] to evaluate the binding mode and the stability of the new series compared to the related INDZ compounds (see Materials and Methods for details). It followed that the docked ligands accommodated the central core in the ATP binding site similarly to the INDZ group, while major variability in the orientations of R_1_ and R_2_ groups was observed. Such conformational variability was mainly ascribed to: (i) the inherent flexibility of the R_1_ appended moieties, (ii) the open solvent-exposed binding site occupied by the R_1_ groups, and (iii) the wide binding site filled by the R_2_ residues. 

**Figure 3:**
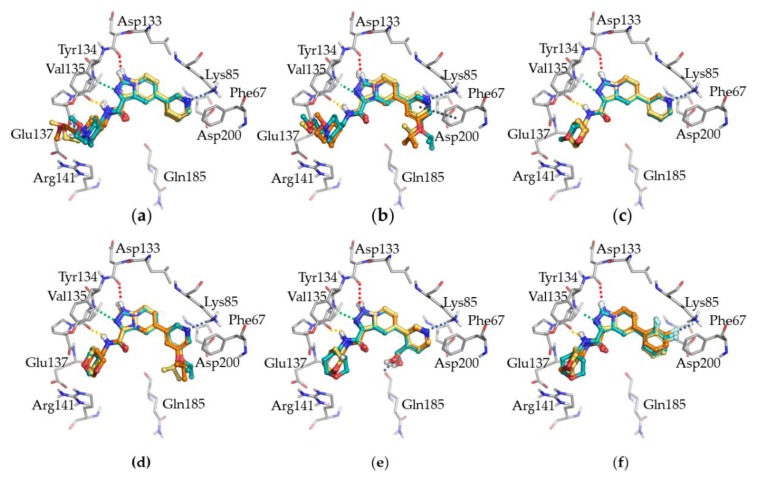
Binding poses of INDZ, IMID 1, and IMID 2 derivatives, reported as yellow, cyan, and orange ball and sticks representation, respectively, in complex with GSK-3β. Each panel represents a triad bearing the same R_1_ and R_2_ groups, (**a**) **1**-**7**-**13**, (**b**) **2**-**8**-**14**, (**c**) **3**-**9**-**15**, (**d**) **4**-**10**-**16**, (**e**) **5**-**11**-**17**, (**f**) **6**-**12**-**18**. The most relevant residues of the ATP binding site are also showed in grey. Deep, central, and outer H-bonds are represented in red, green, and orange, respectively, while dark dashed lines refer to other interactions involving Phe67, Lys85, and Gln185.

Figure 3 highlights the good overlap of INDZ-IMID 1-IMID 2 triads bearing the same R_1_ and R_2_ groups. 

Deep visual inspection and energetics considerations (from −8.95 to −7.72 kcal/mol docking score) led to the selection of one pose per ligand for further studies (Table 2). For a comprehensive understanding of the IMID 1 and IMID 2 binding mode, the RMSD of the triplets was first calculated based on the common substructure of the core scaffold and carboxamide portion (excluding hydrogen atoms), while the more flexible R_1_ and R_2_ groups were disregarded (Table 2). In the match pair analysis, the indazole was considered as reference structure.

The new cores showed little displacement, in the range 0.15–0.67 Å RMSD, indicating very high similarity to each other in the binding site. For sake of completeness, RMSD of the common substructure of the whole molecules is also reported in Table 2, showing more variability within each triplet when R_1_ and R_2_ groups are considered.

In this work, MD was intended as a postprocessing tool to validate and refine Glide SP docking solutions. In addition, we aimed to evaluate the complex stability since co-crystal structures were unavailable at that time. The basic assumption was that a bad docking pose would have generated an unstable MD trajectory, during which the ligand could even leave the binding site. Conversely, a meaningful docking pose would have displayed stable and specific interactions with the target, showing a low RMSD over time, with respect to the starting conformation [33]. 

Based on the docking results, compounds seemed to reasonably occupy the ATP binding site of GSK-3β kinase. To confirm this hypothesis, we ran MD simulations of all derivatives using Desmond (see Materials and Methods). To define the optimum MD time scale, 500 ns simulations of **2**, **8**, and **14** in complex with GSK-3β enzyme were initially run. After a deep analysis, we found that the first 100 ns were representative of the overall 500 ns trajectories. In fact, the main structural events were generally preserved and the interaction fraction with the most relevant residues of the binding site were comparable (Figure S1 in Supplementary Materials). Therefore, a 100 ns time scale was picked for all compounds as a good compromise between structural sampling thoroughness and computational cost. A single 100 ns MD simulation per compound was assumed to provide exhaustive conformational sampling given that the same combination of the R1 and R2 groups (considered the most fluctuating portions of the compounds) was simulated three times within each triplet. 

**Figure 4:**
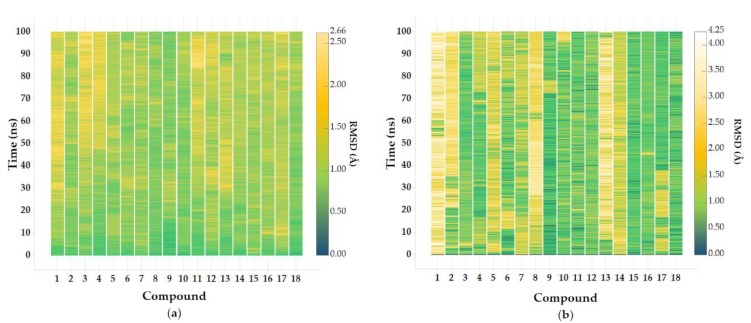
RMSD evolution of (**a**) protein and (**b**) ligand with respect to the protein, for each 100 ns MD simulation using frame 0 as reference. The RMSD ranges are 0–2.66 Å in a and 0–4.25 Å in b (dark green-yellow-white color scale corresponds to 0 to 4.25Å values).

Both protein and ligand RMSDs from the initial structures were calculated for each MD trajectory showing good structural stability of the whole systems (Figure 4). In detail, the former spanned 0 to 2.66 Å and the latter 0 to 4.25 Å. Larger ligand RMSD values were observed in compounds bearing the R_1_ piperidine group, which fluctuated in the solvent-exposed pocket. For each complex, RMSD of protein and ligand resulted to be quite comparable, meaning that the ligand thoroughly occupied the initial binding site without diffusing away. Ligand RMSF was also evaluated to investigate the most flexible moieties of the inhibitors (Figure S2). In all systems, R_1_ and R_2_ groups showed the highest RMSF values compared to the stable core scaffolds. 

The pattern of H-bond interaction involving the hinge residues was essentially maintained along all the simulations. In detail, Val135 carbonyl interacted with carboxamide N-Hb via stable H-bond with comparable persistence between INDZ and corresponding IMID 1 and IMID 2 compounds along the MD trajectories. Conversely, the interaction occurrence of the central H-bond within each triad was variable, with INDZ derivatives contacting Val135 more persistently than IMID 1 and IMID 2 analogues (Table S1). The inner H-bond involving Asp133 was found in all systems although interaction distances and angles changed from INDZ to IMID 1 and IMID 2 core scaffolds (see below). These results suggest that INDZ derivatives are more robustly anchored to the hinge residues than IMID 1 and IMID 2 analogues. Nevertheless, none of the simulations run on the three cores showed ligands moving away from the binding pocket. The amide carbonyl was exposed to water molecules through which it interacted with Ile62 and Gln185 to a different extent in all trajectories spanning from 10 up to 50% persistence (Figure S3). Albeit not preserved during the entire simulations, this along with the outer H-bond seemed to stabilize the amide group preventing its rotation. Different results were obtained for the analyzed R_1_ and R_2_ groups. In **1-7**-**13** and **2**-**8**-**14** systems, the highly flexible piperidine group fluctuated in the open solvent-exposed pocket and contacted Gln72 and Pro136 via intermittent direct or indirect interactions (Figure S3). A water-bridged H-bond involving the R_1_ oxanyl ring was mostly observed with Pro136 in the remaining systems (Figure S3). Other solvent-mediated contacts were found along all MD simulations between the R_1_ group and Tyr134, Glu137, and Arg141. Overall, such indirect bonds randomly observed along the simulations reflected the high flexibility of the R_1_ groups and the open binding pocket in which they lied. On the other side, aromatic and heteroaromatic rings variably interacted with the surrounding residues. In the **1**-**7**-**13** triad, the pyridine system stably occupied the region around the positively charged Lys85 residue, but optimal geometry for a stable contact was found to a variable extent in the three systems. Pi–pi interaction between the heteroaromatic ring and Phe67 was detected along the trajectories moving the nitrogen atom away from Lys85. The meta-pyridinyl substituent of **2**, **8**, and **14** derivatives was found to engage Asp200 from the highly conserved motif DFG via a water-mediated H-bond. Consequently, the pyridinyl ring fairly rotated to ease the rearrangement of the 5-substituent preventing a stable contact of its nitrogen atom with Lys85. De facto, this charged-assisted H-bond occurred no more than 30% of the simulation time in **2** and **8** trajectories and was not found in the **14** system. Moving to derivatives **3** and **15**, the unsubstituted pyridinyl ring preserved the contact with the positively charged Lys85 for over 70% MD simulations. Instead, rotation of pyridine ring in **9** moved away the nitrogen atom resulting in 50% occurrence of such an H-bond. The R_2_ group was also placed towards Phe67 in a pi–pi stacking interaction. Next, compounds **10** and **16** showed none or weak interaction with Lys85, while the isopropoxyl group confirmed the anticipated interaction with Asp200 via a water molecule. The corresponding INDZ derivative **4** contacted Lys85 more persistently and the oxygen atom of the meta substituent underwent also an indirect interaction with Gln185 (Figure S3). The indirect contact with Asp200 occurred around 50% of the simulation time in all systems with the isopropoxyl group. Compared to the interaction pattern observed in unsubstituted pyridinyl derivatives, this additional bond could contribute to ameliorated affinity for GSK-3β. In the **5**-**11**-**17** triad, the R_2_ hydroxymethyl group contacted Gln185 through a direct or water-mediated H-bond (Figure S3). An indirect interaction of the ortho substituent with Asn186 was also observed. In addition, Lys85 occasionally contacted the pyridinyl nitrogen of **5**. Finally, the **6**-**12**-**18** triad mostly orientated the halogen atoms of the di-F-phenyl group towards Lys85 so promoting an electrostatic-like interaction (Figure S3).

In **1**–**18** systems, hydrophobic contacts were also observed where ligands engaged Ile62, Val70, Ala83, Leu132, Leu188, and Tyr134 at different extents. A timeline representation of the contacts (H-bonds, hydrophobic, ionic, water bridges) with protein is reported in Figure S4. 

On the whole, ligand docking and MD simulations provided valuable structural information about the new IMID 1 and IMID 2 molecules. The initial binding poses appeared to be stable with preserved contacts across the MD trajectories, with subtle diversity of central and deep H-bonds. Thus, the newly designed compounds were basically suggested as comparable to the previous INDZ analogues.

### 2.4. In Vitro Data of Novel IMID 1 and IMID 2 Derivatives

Driven by the promising results, compounds **7**–**18** were synthesized as reported in Chemistry Section 2.1. Their biological activity was determined using an in vitro GSK-3β assay described elsewhere [17,19]. It followed that **7**–**18** exhibited good inhibition activity mostly in the nanomolar scale with an approximate 8- to 50-fold increase in IC_50_ with respect to **1**–**6** (Table 1). In vitro MDCK-MDR1 permeability of IMID 1 and IMID 2 derivatives was also experimentally assessed (Table 1). Derivatives **12**, **15**, and **18** resulted in improved permeability compared to their INDZ analogues. In this respect, a decrease of TPSA was observed moving from INDZ **6** to the corresponding analogues **12** (IMID 1) and **18** (IMID 2), and from **3** to **15**. The SAR analysis highlighted the detrimental impact of the positively charged piperidine ring of **2**-**8**-**14** triplet and derivative **13** on CNS permeability. In fact, these compounds showed low P_app_ values. When the piperidine was replaced with an oxanyl ring, while keeping the isopropoxypyridinyl group on the other side (**2** vs. **4** and **8** vs. **10**), a low CNS penetration was still observed, indicating a coexisting unfavorable contribution (high TPSA) of the R_2_ moiety to permeability. The comparison of the **4**–**10** pyridinyl derivatives with the corresponding **6**–**12** di-F-phenyl molecules confirmed an ameliorated BBB permeability when lipophilicity raises. 

In the new series of compounds, a general reduction of the ER values was also observed. This was consistent with published guidelines according to which removal of H-bond groups or reduction of H-bond strength can reduce P-gp efflux [34]. Moreover, it is known that compounds with the most basic pK_a_ of less than 8.0 have a much higher likelihood of being identified as a non-P-gp substrate [34]. This unambiguously emerged from the measured ER of **8**, **13**, and **14** and by comparing derivatives **8** and **10** and **13** and **15**.

Hence, the optimization of physicochemical properties and the introduction of focused chemical modifications to contain the critical HBD count of IMID 1 and IMID 2 series enabled to successfully overcome the indazole issues for CNS penetration. 

### 2.5. Retrospective In Silico Analyses and NMR Studies to Rationalize in Vitro Data 

**Figure 5:**
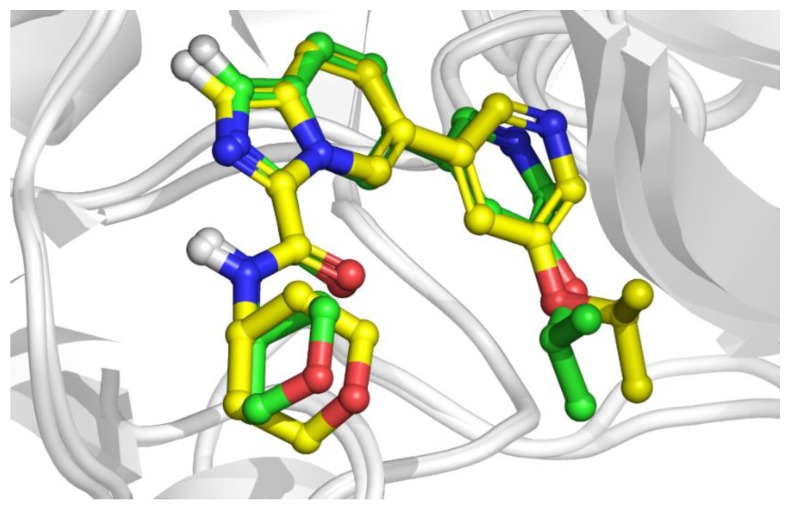
Overlap of the docking poses (yellow) versus co-crystal structure (green) of **16** in the complex with GSK-3β enzyme.

This far, ligand docking and MD simulations were described driving the INDZ optimization campaign in a predictive way. To strengthen the results of the calculations, we generated a high-resolution X-ray structure of **16** in complex with the protein (PDB entry 6Y9S). In line with our prediction, **16** confirmed to inhibit GSK-3β enzyme by binding within the ATP pocket. The tridentate interaction with the hinge residues, the oxanyl ring towards Arg141, R_2_ group contacting Lys85, and a pool of water molecules towards the open cavity and the back pocket were found. Compared to the docked structure, a very good consensus was observed with core RMSD = 0.35 Å (Figure 5), thus validating our models. The experimental binding mode also confirmed the stable water-bridged contact of the isopropoxyl group with Asp200 detected in the MD simulation (Figure S3). 

Thus, we carried out other in silico analyses starting from MD simulations to elucidate retrospectively the factors responsible for the unanticipated potency decrease of the new series. Due to the highly similar inhibition activity, the following is not meant to provide a rank of IMID 1 and IMID 2 compounds. Our results are intended to compare the new derivatives with the INDZ analogues instead, regardless of the tiny IC_50_ differences within each core series.

First, the average binding free energy (ΔG_bind) based on the molecular mechanics-generalized born surface area (MM-GBSA) was calculated from each production trajectory [35,36]. Interestingly, the INDZ molecules always resulted in better MM-GBSA scores compared to the corresponding IMID 1 and IMID 2 derivatives, in agreement with the in vitro potency (Table 3). In fact, energetics terms turned out to be generally more favorable for the INDZ compounds within each triplet.

In both the IMID series, we supposed the drop in potency to be attributable to the less acidic C-Ha group of the central core. Together with other moieties, it tightly anchors the compounds in the binding pocket, but the nature of the H-bond is weaker compared to the one involving conventional INDZ N-Ha. The general characteristics of this type of interaction to take into account are: (i) the distance between the hydrogen and the acceptor (A), (ii) the angle formed by the donor (D), the hydrogen itself and A, and (iii) the strength [37,38]. To different levels, these components were herein investigated across the molecules. 

**Figure 6:**
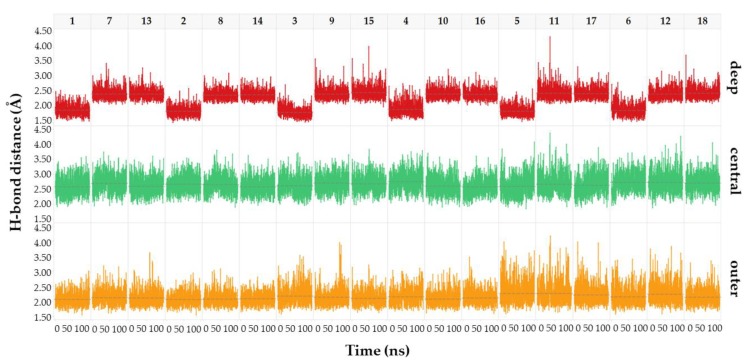
Distance of the deep (red), central (green) and outer (orange) H-bond interactions across 100 ns MD simulation of derivatives **1**–**18**. The plots are ordered per INDZ-IMID 1-IMID 2 triplet. Dashed lines represent the mean value of the H-bond distance.

We examined how the binding mode changes as the core scaffolds are varied, in terms of distance between each hinge binding atom of the ligands and the partner heavy atom of Asp133 and Val135 along the MD simulations. The analysis revealed that moving from INDZ to IMID 1 and IMID 2 analogues, an elongation of the deep H-bond occurred, while the outer and central interactions remained approximately constant in length (red versus green and orange scatter plots in Figure 6). The stretching of the deep H-bond was found in IMID 1 and IMID 2 compounds of all triplets regardless of the R_1_ and R_2_ moieties. This suggests a possible rotation of the ligand about the hinge binding residues which might affect the SAR and elucidate the potency reduction. As anticipated in the MD discussion, persistence percentage of the central H-bond is higher in INDZ compounds compared to IMID 1 and IMID 2 analogues. The optimally spaced deep H-bond of the INDZ system might reflect on such high persistence. Conversely, the central H-bond is more scattered along the simulations of IMID 1 and IMID 2 derivatives as a consequence of the elongated interaction of the C-Ha. 

**Figure 7:**
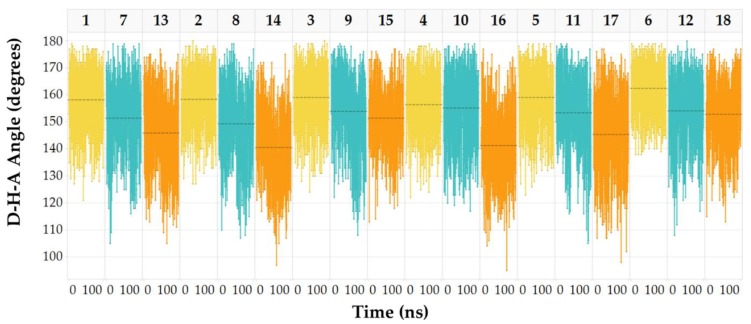
D-H-A angles (degrees) of the deep H-bond interaction across 100 ns simulation of **1**–**18** systems. The plots are ordered per INDZ-IMID 1-IMID 2 triplet and colored as yellow, cyan, and orange, respectively. Dashed lines correspond to the mean value of the D-H-A angle.

In the MD trajectories, optimal D-H-A angles promoting strong interaction of the INDZ N-Ha with the hinge portion were also observed, while the values lowered moving to IMID 1 and IMID 2 cores (Figure 7). 

Based on the observed arrangements, we may ascribe the H-bond between IMID C-Ha and Asp133 carbonyl group to a weakened interaction compared to INDZ N-Ha, so substantiating the affinity decrease of IMID 1 and IMID 2 derivatives versus the GSK-3β enzyme. 

Lastly, the H-bond acidity prediction was investigated via QM methods using Jaguar software [39]. The Vα(r) descriptor of the core Ha and the carboxamide Hb was calculated. This is an effective and useful predictor of H-bond strength defined in Peter Kenny’s work and recently implemented in Schrödinger suite [40]. Larger values correlate with stronger HBDs, while HBAs are characterized by low numbers. Although Kenny’s analysis never really considered C-H donors, the principle of the calculation is still well defined. Here, all ligands from Glide SP docking were used as input for Vα(r) calculation. (Table 4). 

The calculation indicated that the INDZ core has the strongest H-bond potential in all compounds, with a very strong N-Ha donor. This illustrates the importance of this moiety at the hinge for good affinity, but it equally explains the reason why it is felt to be an issue for CNS penetration. The calculations also showed that the C-Ha of the IMID 1 and IMID 2 cores are weaker H-bond donors, with some variability between the two new series. In each triplet, there seemed to be a large Vα(r) difference when Ha was compared between INDZ derivatives and the corresponding IMID 1 and IMID 2 analogues (ΔVα(r) > 40). On the other hand, the Vα(r) values between related IMID 1 and IMID 2 compounds within the same triplet resulted in a minor gap. These in silico results appeared to be in line with the experimental IC_50_ values, where potency reduction of one or more orders of magnitude was observed moving from INDZ compounds to the new series, while IMID 1 and IMID 2 derivatives were quite comparably potent as GSK-3β inhibitors. About Hb, no big changes were observed from INDZ to IMID 1 and IMID 2 scaffolds. This suggested that the acidic N-Ha/C-Ha group was a significant discriminant of the cores for inhibition activity, while the carboxamide N-Hb did not make a real difference. With this, we proved Vα(r) to be a valuable descriptor to explore SAR in terms of H-bond acidity. 

These observations were further substantiated using ^1^H-NMR characterization. By way of example, we selected the triplet **2-8-14**. In particular, H-bond donating abilities of the core N-Ha/C-Ha and the carboxamide N-Hb of the three compounds were investigated by means of NMR spectroscopy. In NMR experiments, proton chemical shifts depend upon many factors, among which there are H-bond interactions. In general, a proton involved in such interaction exhibits a decreased shielding and therefore tends to move downfield in the NMR spectrum.

First, the complete assignment of the ^1^H-NMR spectra of **2**, **8** and **14** compounds was carried out in CDCl_3_ by means of the simultaneous ^1^H-^1^H COSY, ^1^H-^1^H NOESY, ^1^H-^13^C HSQC, and ^1^H-^13^C HMBC 2D NMR analyses (see Supplementary Materials), to unequivocally assign the Ha and Hb resonances.

Subsequently, the ^1^H-NMR spectra were acquired in both CDCl_3_ and DMSO-d_6_ as “not good” and “good” H-bond acceptor solvents, respectively [41]. The variation of chemical shifts of Ha and Hb protons in the two solvents was calculated as Δδ (δ_DMSO_ − δ_CDCl3_), using the TMS signal as a reference at 0 ppm (Table 5 and Figures S5–S7). The target concentration of the compounds was selected by a serial dilution method (reaching a 0.3 mM final concentration), until a stable chemical shift difference between the two solvents was observed, and the solute–solute interactions were expected as low as possible.

The large chemical shift difference of 3.4 ppm observed for proton Ha in compound **2** going from DMSO-d_6_ to CDCl_3_ suggested that this proton was involved in H-bond with the former solvent, excluding the formation of an intramolecular H-bond. On the contrary, the decrease in Δδ observed for proton Ha of compounds **8** and **14** suggested a lower acidity of the IMID 1 and IMID 2 core scaffolds. This is in agreement with the pharmacological results where potency reduces moving from the INDZ compounds to the new series, whereas IMID 1 and IMID 2 derivatives are similarly double-digit nanomolar GSK-3β inhibitors. On the other hand, no large differences were observed for the Δδ of Hb within the triplet (Table 5), suggesting comparable acidity of the carboxamide N-Hb in the three different scaffolds. Moreover, the sharper and better resolved triplet observed for the Hb signals moving from CDCl_3_ to DMSO-d_6_ (Figures S5–S7) confirmed the involvement of the protons in H-bond interactions with the latter solvent, which prevented them from engaging in exchange processes with the residual amounts of water in solution.

Overall, preliminary NMR data suggested, as expected, that the INDZ core has the strongest H-bond potential playing a crucial role in the affinity for GSK-3β binding pocket. Moreover, Vα(r) was in accordance with Δδ, as a significant acidity drop of Ha was observed moving from INDZ to IMID 1 and IMID 2 in both calculated and experimental parameters.

**Figure 8:**
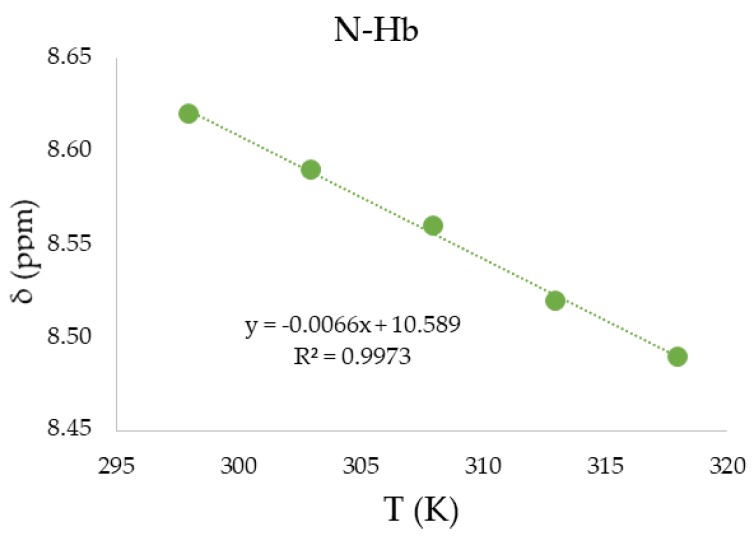
Temperature coefficient calculated for N-Hb of compound **14** (IMID 2).

The proton spectra of the **2**, **8** and **14** were also acquired in deuterated DMSO at five different temperatures (298, 303, 308, 313, and 318 K). The temperature coefficients (T_coeff_) were calculated for N-Hb protons by linear fitting of the chemical shift as function of temperature curves (as exemplified in Figure 8 for compound **14**).

In general, amide proton chemical shifts show a strong temperature dependence and this is mainly due to the occurrence of H-bonds [42]. Chemical shifts depend on the inverse third power of the distance between amide proton and H-bond acceptor; the average H-bond length increases with temperature because of the increased thermal motions [43]. Therefore, the deshielding effect induced by an acceptor decreases at higher temperature and amide protons result in being shifted upfield. In line with this general trend, N-Ha and N-Hb chemical shifts followed this behavior and showed a linear dependence with temperature, allowing the determination of T_coeff_ values from the slope of the curves (Table 6).

Hb atoms have shown similar T_coeff_, more negative than that of the Ha proton in the INDZ core. Indeed, for weaker H-bonds, structural perturbations are more pronounced and N-Hb chemical shifts are more temperature dependent (more negative). In previous studies, T_coeff_ values in the range of -12.3 to −0.5 ppb/K were reported for -OH groups which were solvent exposed and involved in intermolecular H-bonds [44], while a mean value equal to −7.1 ± 2.5 ppb/K was determined for the solvent-exposed amide protons of the protein backbone [42]. In our case, the obtained values reflected the presence of weak intermolecular H-bonds in DMSO-d_6_; the more negative T_coeff_ values for the Hb atoms suggested that, by increasing the temperature, intermolecular solute-solvent H-bonds are broken more easily for N-Hb than for N-Ha within the INDZ core, in agreement with the conclusions drawn in light of the Δδ values. 

From the Δδ downfield shifts reported above, it was possible to estimate the H-bond energies *E_HB_*. The most universal approach is the empirical estimation of the H-bond strength by use of the Equation (3) (see Materials and Methods) [45]. The equation is only valid for weak and moderate values of the H-bond energy. The energy varies within a wide range from weak (1–4 kcal/mol) to medium (4–15 kcal/mol) to strong H-bonds (15–40 kcal/mol).

Based on the calculated values (Table 7), Ha of compound **2** showed the highest energy, while comparable and lower values were observed concerning Hb, regardless of the central core. Accordingly, we can assert that Ha of INDZ and Hb of all core scaffolds are involved in intermolecular H-bonds that can be classified as weak/moderate to highly weak, respectively. Again, this outcome enables to rationalize SAR confirming the key role of the INDZ N-Ha for the high inhibition activity. 

## 3. Materials and Methods

### 3.1. Chemistry

Reagents were purchased from Sigma-Aldrich (Madrid, Spain) and Fluorochem (Hadfield, UK) and used as received.

Reaction progress was monitored by TLC or LC-MS. For TLC analysis, Merck silica gel 60 F254 (0.04–0.063 mm) plates with detection by UV (214 or 254 nm) were used. LC-MS analyses were performed on the Shimadzu LCMS-2020 Single Quadrupole Liquid Chromatograph Mass Spectrometer (Duisburg, Germany) and column Acquity UPLC C18 (2.1 × 50 mm, 1.8 µm) (Etten-Leur, The Netherlands).

Final compound purity (always >95%) was determined by HPLC-UV, by comparing the area of the target peak and total peak areas of minor impurities. HPLC-UV analyses were performed with a pump/autosampler (Waters 2695, Alliance model), a UV photo diode array detector (Waters 2996 model), and a Waters system data management (Empower 2) (Etten-Leur, The Netherlands). The column used was generally X-Bridge C18 (150 × 4.6 mm, 5 μm). 

UPLC-QTOF analyses for HRMS determination of final compounds were carried out by means of a SYNAPT MS-ACQUITY UPLC system (Waters). The system was operated in positive ion mode in the “V-Optics” configuration. Leucine-enkephalin (200 pg/μL) was employed as the lock mass in order to provide authenticated exact mass measurement in MS and MS/MS modes within 5 ppm RMS mass accuracy. The column was an Acquity BEH C18 (50 × 2.1 mm, 1.7 μm). 

Nuclear Magnetic Resonance Spectroscopy (^1^H-NMR and ^13^C-NMR) were obtained using Bruker Advance systems (Milan, Italy), operating at 300, 400 and 500 MHz. Spectra were acquired at 300 K, using DMSO-d_6_, CD_3_OD, and CDCl_3_ as solvents. All resonance bands were referenced to tetramethylsilane (internal standard). For ^1^H-NMR spectroscopy: (s) = singlet; (d) = doublet; (t) = triplet; (br) = broad; (dd) = double doublet; (dt) = double triplet; (ddd) = double double doublet; (dtd) = double triple doublet; (m) = multiplet; *J*= coupling constant; and δ = chemical shift (in ppm).

Compounds were purified with one of the following techniques: (i) flash chromatography on silica gel (Grace Reveleris flash chromatography system with a 40 μM silica cartridge, flow = 60 mL/min), with an appropriate gradient of mixtures of DCM/MeOH or Cyclohexane/EtOAc as eluents; (ii) preparative HPLC-MS system (sample was dissolved at 50 mg/mL in DMSO/CH_3_CN in 1:1 ratio), using an appropriate gradient of CH_3_CN and H_2_O + 0.1% NH_4_HCO_3_; flow =40 mL/min. The preparative HPLC-MS system consisted of a Waters 2767 Sample manager, a Waters 2478 dual λ absorbance detector, and a Waters Micromass ZQ single quadrupole mass spectrometer with an electrospray ionization (ESI) source. The column used was a X-Bridge Prep C18 5 μm OBD with 19 × 10 mm (Waters) precolumn. Fraction collection was available from the system software MassLynx v. 4.1 (Etten-Leur, The Netherlands). Detection wavelength was set to 230 nm and temperature to 25 °C. 

#### General Procedures (A)–(D):

*General amide coupling procedure (A) for the synthesis of compounds ***21***, ***22*** and ***13**–**18***.*

To a solution of carboxylic acid/lithium carboxylate salt (1.0 eq.) in DMF (0.07 M) HATU (1.2 eq.), DIPEA (4.5 eq.) and the proper amine (1.0–1.2 eq.) were added. The solution was stirred at rt until full conversion was observed by HPLC-MS (24–72 h). The solvent was evaporated under reduced pressure, the residue was taken up with EtOAc and washed with 1N aqueous NaOH solution (×2) and brine. The combined organic phases were dried over anhydrous MgSO_4_, filtered and concentrated in vacuo. The crude was purified by flash chromatography (SiO_2_, DCM/MeOH for **21** and **22**) or reverse phase chromatography (C18, NH_4_HCO_3_/ACN for **13**–**18**) to give the target compound.

*General boronate synthesis procedure (B) for the synthesis of compounds ***23*** and ***25***.*

A mixture of bromide (1.0 eq.), bis(pinacolato)diboron (2.0 eq.) and potassium acetate (2.0 eq.) in 1,4-dioxane (0.12 M) was placed in a Schlenk tube. After degassing with N_2_ for 10 min, Pd(dppf)Cl_2_ (0.15 eq.) was added and the mixture was heated at 100 °C overnight. Conversion was checked by HPLC-MS. The mixture was diluted with EtOAc/MeOH, the insoluble solids were removed by filtration through Celite and the solvent was evaporated under reduced pressure. The crude was purified by flash chromatography (SiO_2_, DCM/MeOH) to give the target compound.

*General Suzuki coupling procedure (C) for the synthesis of compounds ***26***–***29*** and ***7***–***12***.*

A mixture of bromide (1.0 eq.), boronic acid/ester (1.0 eq.) and cesium fluoride (2.0 eq.) in 1,4-dioxane and water (4:1, 0.1M) was placed in a Schlenk tube. After degassing with N_2_ for 10 min, Pd(dppf)Cl_2_ (0.1 eq.) was added and the mixture was heated at 90 °C overnight. Conversion was checked by HPLC-MS. The mixture was diluted with EtOAc/MeOH, the insoluble solids were removed by filtration through Celite, and the solvent was evaporated under reduced pressure. The crude was purified by flash chromatography (SiO_2_, Cyclohexane/EtOAc for **26**, **27** and **29**; DCM/MeOH for **28**) or reverse phase chromatography (C18, NH_4_HCO_3_/ACN for **7**–**12**) to give the target compound.

*General ester hydrolysis procedure (D) for the synthesis of compounds ***30***–***33***.*

To a solution of ethyl carboxylate ester (1.0 eq.) in MeOH and water (1:1, 0.03 M), lithium hydroxide hydrate (2.0 eq.) was added. Conversion was checked by HPLC-MS. After stirring at 50 °C overnight, the reaction mixture was concentrated to dryness under reduced pressure to give the target compound that was used in the next step without further purification.

*7--Bromoimidazo[1,5-a]pyridine-1-carbaldehyde* (**19**). To a solution of 7-bromoimidazo[1,5-*a*]pyridine (500 mg, 2.54 mmol) in toluene (2.5 mL), DMF (0.22 mL, 2.87 mmol) and phosphorus oxychloride (0.33 mL, 3.55 mmol) were added. After stirring at 100 °C for 1.5 h, the reaction mixture was carefully quenched with ice and 1N aqueous NaOH solution. The aqueous phase was extracted with DCM (×3), the combined organic layers were dried over anhydrous MgSO_4_, filtered and evaporated under reduced pressure. The crude was purified by flash chromatography (SiO_2_, Cyclohexane/EtOAc), affording 327 mg of the title compound. Yield: 57%. LC-MS (ESI) *m/z*: 224.9 [M + H]^+^.

*7-Bromoimidazo[1,5-a]pyridine-1-carboxylic acid* (**20**). A mixture of 7-bromoimidazo[1,5-*a*]pyridine-1-carbaldehyde **19** (519 mg, 2.306 mmol), silver nitrate (509 mg, 3.00 mmol), and sodium hydroxide (277 mg, 6.92 mmol) in water (4 mL) was stirred at rt for 3 h. The reaction was then diluted with water and the black solid was filtered off and washed with water. The filtrate was made acidic with 1N aqueous HCl solution and the resulting precipitate was filtered and dried. The crude (375 mg) was used in the next step without further purification. Yield: 61%. LC-MS (ESI) *m/z*: 240.9 [M + H]^+^.

*7-Bromo-N-{[1-(2-methoxyethyl)piperidin-4-yl]methyl}imidazo[1,5-a]pyridine-1-carboxamide* (**21**). The title compound was obtained according to general procedure (A) using 7-bromoimidazo[1,5-a]pyridine-1-carboxylic acid **20** (0.73 mmol) and 1-[1-(2-methoxyethyl)piperidin-4-yl]methanamine (0.73 mmol). Yield = 56% (200 mg). LC-MS (ESI) *m/z*: 395.1 [M + H]^+^. ^1^H-NMR (400 MHz, CD_3_OD) δ 8.35-8.32 (m, 1H), 8.30 (s, 1H), 8.28 (dd, 1H, *J* = 7.4, 0.9 Hz), 6.94 (dd, 1H, *J* = 7.4, 1.9 Hz), 3.72–3.68 (m, 2H), 3.57 (d, 2H, *J* = 10.3 Hz), 3.40 (s, 3H), 3.38 (d, 2H, *J* = 6.6 Hz), 3.30-3.26 (m, 1H), 3.06–2.93 (m, 3H), 2.07–1.90 (m, 3H), 1.66–1.53 ppm (m, 2H).

*7-Bromo-N-[(oxan-4-yl)methyl]imidazo[1,5-a]pyridine-1-carboxamide* (**22**). The title compound was obtained according to general procedure (A) using 7-bromoimidazo[1,5-a]pyridine-1-carboxylic acid **20** (0.73 mmol) and 1-(oxan-4-yl)methanamine (0.73 mmol). Yield = 78% (192 mg). LC-MS (ESI) *m/z*: 338.0 [M + H]^+^. ^1^H-NMR (400 MHz, CD_3_OD) δ 8.33 (dt, 1H, *J* = 1.8, 0.7 Hz), 8.27 (s, 1H), 8.25 (dd, 1H, *J* = 7.4, 1.0 Hz), 6.91 (dd, 1H, *J* = 7.4, 2.0 Hz), 3.95 (dd, 2H, *J* = 11.4, 2.6 Hz), 3.40 (td, 2H, *J* = 11.9, 2.1 Hz), 3.30 (d, 2H, *J* = 6.9 Hz), 1.94-1.82 (m, 1H), 1.74-1.65 (m, 2H), 1.42-1.29 ppm (m, 2H).

*N-((Tetrahydro-2H-pyran-4-yl)methyl)-7-(4,4,5,5-tetramethyl-1,3,2-dioxaborolan-2-yl)imidazo[1,5-a]pyridine-1-carboxamide* (**23**). The title compound was obtained according to general procedure (B) using 7-bromo-N-[(oxan-4-yl)methyl]imidazo[1,5-a]pyridine-1-carboxamide **22** (1.36 mmol). Yield = 76% (340 mg). LC-MS (ESI) *m/z*: 386.2 [M + H]^+^. ^1^H-NMR (400 MHz, CD_3_OD) δ 8.62 (s, 1H), 8.31 (s, 1H), 8.27 (dd, 1H, *J* = 7.0, 1.2 Hz), 6.99 (dd, 1H, *J* = 7.0, 1.0 Hz), 3.95 (dd, 2H, *J* = 11.4, 2.7 Hz), 3.41 (td, 2H, *J* = 11.9, 2.1 Hz), 3.34-3.31 (m, 2H), 1.96–1.84 (m, 1H), 1.71 (dd, 2H, *J* = 13.1, 1.8 Hz), 1.43–1.28 (m, 2H), 1.36 ppm (s, 12H).

*Ethyl 6-(4,4,5,5-tetramethyl-1,3,2-dioxaborolan-2-yl)imidazo[1,5-a]pyridine-3-carboxylate* (**25**). The title compound was obtained according to general procedure (B) using 6-bromoimidazo[1,5-*a*]pyridine-3-carboxylate **24** (11.0 mmol). Yield = 100% (4.08 g). LC-MS (ESI) *m/z*: 317.2 [M + H]^+^. ^1^H-NMR (400 MHz, CDCl_3_) δ 9.70 (dd, 1H, *J* = 2.0, 1.0 Hz), 7.61–7.57 (m, 2H), 7.36–7.30 (m, 1H), 4.51 (q, 2H, *J* = 7.2 Hz), 1.49 (t, 3H, *J* = 7.1 Hz), 1.36 ppm (s, 12H, *J* = 3.3 Hz).

*Ethyl 6-(pyridin-3-yl)imidazo[1,5-a]pyridine-3-carboxylate* (**26**). The title compound was obtained according to general procedure (C) using 6-bromoimidazo[1,5-*a*]pyridine-3-carboxylate **24** (0.55 mmol) and pyridin-3-ylboronic acid (0.55 mmol). Yield = 64% (94 mg). LC-MS (ESI) *m/z*: 268.1 [M + H]^+^.

*Ethyl 6-{5-[(propan-2-yl)oxy]pyridin-3-yl}imidazo[1,5-a]pyridine-3-carboxylate* (**27**). The title compound was obtained according to general procedure (C) using 6-bromoimidazo[1,5-*a*]pyridine-3-carboxylate **24** (0.45 mmol) and {5-[(propan-2-yl)oxy]pyridin-3-yl}boronic acid (0.45 mmol). Yield = 93% (140 mg). LC-MS (ESI) *m/z*: 326.1 [M + H]^+^.

*Ethyl 6-[4-(hydroxymethyl)pyridin-3-yl]imidazo[1,5-a]pyridine-3-carboxylate* (**28**). The title compound was obtained according to general procedure (C) using ethyl 6-(4,4,5,5-tetramethyl-1,3,2-dioxaborolan-2-yl)imidazo[1,5-a]pyridine-3-carboxylate **25** (0.68 mmol) and (3-bromopyridin-4-yl)methanol (0.68 mmol). Yield = 51% (105 mg). LC-MS (ESI) *m/z*: 298.1 [M + H]^+^.

*Ethyl 6-(2,3-difluorophenyl)imidazo[1,5-a]pyridine-3-carboxylate* (**29**). The title compound was obtained according to general procedure (C) using 6-bromoimidazo[1,5-*a*]pyridine-3-carboxylate **24** (1.16 mmol) and (2,3-difluorophenyl)boronic acid (1.16 mmol). Yield = 90% (318 mg). LC-MS (ESI) *m/z*: 303.1 [M + H]^+^.

*Lithium 6-(pyridin-3-yl)imidazo[1,5-a]pyridine-3-carboxylate* (**30**). The title compound was obtained according to general procedure (D) using ethyl 6-(pyridin-3-yl)imidazo[1,5-a]pyridine-3-carboxylate **26** (0.35 mmol). Yield = 100% (85 mg). LC-MS (ESI) *m/z*: 240.1 [M + H]^+^. ^1^H-NMR (400 MHz, CD_3_OD) δ 9.66 (s, 1H), 8.91 (d, 1H, *J* = 1.8 Hz), 8.63 (dd, 1H, *J* = 4.9, 1.5 Hz), 8.22 (ddd, 1H, *J* = 8.0, 2.2, 1.7 Hz,), 7.95 (dd, 1H, *J* = 9.4, 0.9 Hz), 7.74 (s, 1H), 7.61 (dd, 1H, *J* = 7.7, 4.7 Hz), 7.54 ppm (dd, 1H, *J* = 9.4, 1.5 Hz).

*Lithium 6-{5-[(propan-2-yl)oxy]pyridin-3-yl}imidazo[1,5-a]pyridine-3-carboxylate* (**31**). The title compound was obtained according to general procedure (D) using ethyl 6-{5-[(propan-2-yl)oxy]pyridin-3-yl}imidazo[1,5-a]pyridine-3-carboxylate **27** (0.43 mmol). Yield = 95% (123 mg). LC-MS (ESI) *m/z*: 298.1 [M + H]^+^. ^1^H-NMR (400 MHz, CD_3_OD) δ 9.63 (s, 1H), 8.44 (d, 1H, *J* = 1.6 Hz), 8.28 (d, 1H, *J* = 2.5 Hz), 7.93 (d, 1H, *J* = 9.1 Hz), 7.74 (s, 1H), 7.71–7.69 (m, 1H), 7.52 (dd, 1H, *J* = 9.4, 1.2 Hz), 4.87–4.78 (m, 1H), 1.40 ppm (d, *J* = 6.0 Hz, 6H).

*Lithium 6-(4-(hydroxymethyl)pyridin-3-yl)imidazo[1,5-a]pyridine-3-carboxylate* (**32**). The title compound was obtained according to general procedure (D) using ethyl 6-[4-(hydroxymethyl)pyridin-3-yl]imidazo[1,5-a]pyridine-3-carboxylate **28** (0.35 mmol). Yield = 100% (97 mg). LC-MS (ESI) *m/z*: 270.1 [M + H]^+^.

*Lithium 6-(2,3-difluorophenyl)imidazo[1,5-a]pyridine-3-carboxylate* (**33**). The title compound was obtained according to general procedure (D) using ethyl 6-(2,3-difluorophenyl)imidazo[1,5-a]pyridine-3-carboxylate **29** (1.05 mmol). Yield = 93% (273 mg). LC-MS (ESI) *m/z*: 275.1 [M + H]^+^.

*N-((1-(2-Methoxyethyl)piperidin-4-yl)methyl)-7-(pyridin-3-yl)imidazo[1,5-a]pyridine-1-carboxamide* (**7**). The title compound was obtained according to general procedure (C) using 7-bromo-*N*-{[1-(2-methoxyethyl)piperidin-4-yl]methyl}imidazo[1,5-a]pyridine-1-carboxamide **21** (0.25 mmol) and pyridin-3-ylboronic acid (0.25 mmol). Yield = 19% (20 mg). HRMS (ESI): calculated 394.2237 [M + H]^+^, found 394.2241 [M + H]^+^. ^1^H-NMR (500 MHz, DMSO-d_6_) δ 8.98 (br s, 1H), 8.64–8.59 (m, 2H), 8.49 (br s, 1H), 8.38 (br s, 1H), 8.17 (br d, 1H, *J* = 7.3 Hz), 8.09 (br s, 1H), 7.54 (br dd, 1H, *J* = 5.0, 6.9 Hz), 7.31 (br d, 1H, *J* = 6.4 Hz), 3.41–3.39 (m, 1H), 3.22 (s, 1H), 3.20–3.18 (m, 2H), 2.84 (br d, 2H, *J* = 10.5 Hz), 2.42 (br t, 2H, *J* = 5.5 Hz), 1.89 (br t, 2H, *J* = 11.0 Hz), 1.62 (br d, 2H, *J* = 12.4 Hz), 1.55 (br s, 1H), 1.21–1.14 ppm (m, 2H). ^13^C-NMR (125 MHz, DMSO-d_6_) δ 163.2, 149.8, 147.8, 134.4, 133.8, 132.4, 131.3, 128.5, 125.4, 125.3, 124.5, 116.1, 113.2, 70.6, 58.4, 57.9, 54.0, 44.2, 36.5, 30.3 ppm.

*7-(5-Isopropoxypyridin-3-yl)-N-((1-(2-methoxyethyl)piperidin-4-yl)methyl)imidazo[1,5-a]pyridine-1-carboxamide* (**8**). The title compound was obtained according to general procedure (C) using 7-bromo-*N*-{[1-(2-methoxyethyl)piperidin-4-yl]methyl}imidazo[1,5-a]pyridine-1-carboxamide **21** (0.25 mmol) and {5-[(propan-2-yl)oxy]pyridin-3-yl}boronic acid (0.25 mmol). Yield = 16% (19 mg). HRMS (ESI): calculated 452.2656 [M + H]^+^, found 452.2652 [M + H]^+^. ^1^H-NMR (500 MHz, CDCl_3_) δ 8.54 (s, 1H), 8.49 (d, 1H, *J* = 1.8 Hz), 8.30 (d, 1H, *J* = 2.7 Hz), 8.07 (dd, 1H, *J* = 0.9, 7.3 Hz), 8.03 (s, 1H), 7.47 (t, 1H, *J* = 2.2 Hz), 7.29 (t, 1H, *J* = 6.0 Hz), 7.02 (dd, 1H, *J* = 1.8, 7.3 Hz), 4.73–4.65 (m, 1H), 3.52 (t, 2H, *J* = 5.7 Hz), 3.39 (t, 2H, *J* = 6.4 Hz), 3.34 (s, 3H), 3.00 (br d, 2H, *J* = 11.0 Hz), 2.58 (t, 2H, J = 5.4 Hz), 2.02 (t, 2H, *J* = 10.8 Hz), 1.80 (br d, 2H, *J* = 12.5 Hz), 1.71–1.63 (m, 1H), 1.52-1.42 (m, 2H), 1.39 ppm (d, 6H, *J* = 6.0 Hz). ^13^C-NMR (125 MHz, CDCl_3_) δ 163.4, 154.3, 139.8, 139.2, 134.4, 132.7, 131.9, 126.6, 125.7, 122.9, 120.2, 117.6, 113.8, 70.9, 70.3, 58.9, 58.2, 54.0, 44.4, 36.3, 29.8, 21.9 ppm.

*7-(Pyridin-3-yl)-N-((tetrahydro-2H-pyran-4-yl)methyl)imidazo[1,5-a]pyridine-1-carboxamide* (**9**). The title compound was obtained according to general procedure (C) using 7-bromo-*N*-[(oxan-4-yl)methyl]imidazo[1,5-a]pyridine-1-carboxamide **22** (0.25 mmol) and pyridin-3-ylboronic acid (0.25 mmol). Yield = 34% (28 mg). HRMS (ESI): calculated 337.1659 [M + H]^+^, found 337.1662 [M + H]^+^. ^1^H-NMR (400 MHz, MeOD) δ 8.95 (dd, *J* = 2.4, 0.8 Hz, 1H), 8.58 (dd, *J* = 4.9, 1.5 Hz, 1H), 8.48–8.46 (m, 2H), 8.34 (s, 1H), 8.23–8.15 (m, 1H), 7.57–7.50 (m, 1H), 7.23 (dd, *J* = 7.7, 1.5 Hz, 1H), 3.96–3.88 (m, 2H), 3.42 (td, *J* = 11.8, 2.1 Hz, 2H), 3.36–3.34 (m, 2H), 1.98–1.85 (m, 1H), 1.77–1.68 (m, 2H), 1.44–1.28 ppm (m, 2H).

*7-(5-Isopropoxypyridin-3-yl)-N-((tetrahydro-2H-pyran-4-yl)methyl)imidazo[1,5-a]pyridine-1-carboxamide* (**10**). The title compound was obtained according to general procedure (C) using 7-bromo-*N*-[(oxan-4-yl)methyl]imidazo[1,5-a]pyridine-1-carboxamide **22** (0.25 mmol) and {5-[(propan-2-yl)oxy]pyridin-3-yl}boronic acid (0.25 mmol). Yield = 28% (30 mg). HRMS (ESI): calculated 395.2078 [M + H]^+^, found 395.2077 [M + H]^+^. ^1^H-NMR (400 MHz, MeOD) δ 8.49 (d, *J* = 1.9 Hz, 1H), 8.46 – 8.43 (m, 2H), 8.34 (s, 1H), 8.23 (d, *J* = 2.7 Hz, 1H), 7.75–7.73 (m, 1H), 7.24–7.20 (m, 1H), 4.83–4.81 (m, 1H), 3.96 (dd, *J* = 11.3, 2.7 Hz, 2H), 3.42 (td, *J* = 11.9, 2.1 Hz, 2H), 3.35 (d, *J* = 6.9 Hz, 2H), 1.91–2.11 (m, 1H), 1.72 (d, *J* = 12.9 Hz, 2H), 1.40 (d, *J* = 6.0 Hz, 6H), 1.42–1.31 ppm (m, 2H).

*7-(4-(Hydroxymethyl)pyridin-3-yl)-N-((tetrahydro-2H-pyran-4-yl)methyl)imidazo [1,5-a]pyridine-1-carboxamide* (**11**). The title compound was obtained according to general procedure (C) using *N*-((tetrahydro-2H-pyran-4-yl)methyl)-7-(4,4,5,5-tetramethyl-1,3,2-dioxaborolan-2-yl)imidazo[1,5-a]pyridine-1-carboxamide **23** (0.35 mmol) and (3-bromopyridin-4-yl)methanol (0.35 mmol). Yield = 48% (12 mg). ^1^H-NMR (400 MHz, MeOD) δ 8.90 (d, *J* = 5.2 Hz, 1H), 8.79 (bs, 1H), 8.74 (dd, *J* = 7.2, 1.1 Hz, 1H), 8.67 (bs, 1H), 8.44 (bs, 1H), 8.04 (d, *J* = 5.1 Hz, 1H), 7.23 (dd, *J* = 7.2, 1.7 Hz, 1H), 4.99 (s, 2H), 4.29–4.24 (m, 2H), 3.72 (td, *J* = 11.9, 2.1 Hz, 2H), 3.65 (d, *J* = 4.1 Hz, 2H), 2.28–2.15 (m, 1H), 2.05–1.98 (m, 2H), 1.72–1.62 ppm (m, 2H).

*7-(2,3-Difluorophenyl)-N-((tetrahydro-2H-pyran-4-yl)methyl)imidazo[1,5-a]pyridine-1-carboxamide* (**12**). The title compound was obtained according to general procedure (C) using 7-bromo-*N*-[(oxan-4-yl)methyl]imidazo[1,5-a]pyridine-1-carboxamide **22** (0.18 mmol) and (2,3-difluorophenyl)boronic acid (0.18 mmol). Yield = 61% (42 mg). HRMS (ESI): calculated 372.1518 [M + H]^+^, found 372.1527 [M + H]^+^. ^1^H-NMR (500 MHz, DMSO-d_6_) δ 8.56 (d, 1H, *J* = 7.2 Hz), 8.51 (s, 1H), 8.30 (s, 1H), 8.16 (br t, 1H, *J* = 6.2 Hz), 7.52–7.47 (m, 2H), 7.35–7.37 (m, 1H), 7.10 (br d, 1H, *J* = 7.2 Hz), 3.83 (br dd, 2H, *J* = 2.4, 11.2 Hz), 3.25 (t, 2H, *J* = 10.0 Hz), 3.19 (t, 2H, *J* = 6.6 Hz), 1.86–1.77 (m, 1H), 1.57 (d, 2H, *J* = 10.0 Hz), 1.27–1.16 ppm (m, 2H). ^13^C-NMR (125 MHz, DMSO-d_6_,) δ 163.2, 150.7, 147.8, 130.9, 129.2, 128.8, 128.7, 125.9, 125.8, 125.6, 124.9, 118.9, 117.8, 114.6, 67.2, 44.3, 35.6, 31.0 ppm.

*N-((1-(2-Methoxyethyl)piperidin-4-yl)methyl)-6-(pyridin-3-yl)imidazo[1,5-a]pyridine-3-carboxamide* (**13**). The title compound was obtained according to general procedure (A) using lithium 6-(pyridin-3-yl)imidazo[1,5-a]pyridine-3-carboxylate **30** (0.40 mmol) and 1-[1-(2-methoxyethyl)piperidin-4-yl]methanamine (0.40 mmol). Yield = 39% (63 mg). HRMS (ESI): calculated 394.2237 [M + H]^+^, found 394.2229 [M + H]^+^. ^1^H-NMR (500 MHz, DMSO-d_6_,) δ 9.70 (s, 1H), 8.92 (d, 1H, *J* = 1.8 Hz), 8.66–8.62 (m, 2H), 8.12 (td, 1H, *J* = 1.9, 7.9 Hz), 7.91 (d, 1H, *J* = 9.3 Hz), 7.64 (s, 1H), 7.55 (dd, 1H, *J* = 4.9, 7.8 Hz), 7.45 (dd, 1H, *J* = 1.4, 9.3 Hz), 3.40 (t, 2H, *J* = 5.9 Hz), 3.22–3.19 (m, 5H), 2.83 (br d, 2H, *J* = 11.3 Hz), 2.42 (t, 2H, *J* = 6.0 Hz), 1.89 (br t, 2H, *J* = 10.9 Hz), 1.63 (br d, 2H, *J* = 12.5 Hz), 1.59–1.56 (m, 1H), 1.22–1.14 ppm (m, 2H). ^13^C-NMR (125 MHz, DMSO-d_6_) δ 159.7, 149.7, 148.0, 134.8, 132.9, 132.5, 130.5, 125.2, 124.5, 122.6, 121.9, 121.0, 119.2, 70.6, 58.4, 57.9, 54.0, 44.4, 36.3, 30.3 ppm.

*6-(5-Isopropoxypyridin-3-yl)-N-((1-(2-methoxyethyl)piperidin-4-yl)methyl)imidazo[1,5-a]pyridine-3-carboxamide* (**14**). The title compound was obtained according to general procedure (A) using lithium 6-{5-[(propan-2-yl)oxy]pyridin-3-yl}imidazo[1,5-a]pyridine-3-carboxylate **31** (0.30 mmol) and 1-[1-(2-methoxyethyl)piperidin-4-yl]methanamine (0.30 mmol). Yield = 46% (60 mg). HRMS (ESI): calculated 452.2656 [M + H]^+^, found 452.2647 [M + H]^+^. ^1^H-NMR (500 MHz, CDCl_3_) δ 9.75 (s, 1H), 8.45 (d, 1H, *J* = 1.5 Hz), 8.31 (d, 1H, *J* = 2.6 Hz), 7.66 (d, 1H, *J* = 9.2 Hz), 7.53-7.50 (m, 1H), 7.49 (s, 1H), 7.42 (t, 1H, *J* = 2.1 Hz), 7.21 (dd, 1H, *J* = 1.1, 9.3 Hz), 4.72 – 4.67 (m, 1H), 3.51 (t, 2H, *J* = 5.6 Hz), 3.38 (t, 2H, *J* = 6.5 Hz), 3.34 (s, 3H), 2.99 (br d, 2H, *J* = 11.4 Hz), 2.57 (t, 2H, *J* =5.7 Hz), 2.01 (br t, 2H, *J* = 11.0 Hz), 1.79 (br d, 2H, *J* = 12.7 Hz), 1.69–1.63 (m, 1H), 1.48–1.42 (m, 1H), 1.40 ppm (d, 1H, *J* = 6.1 Hz). ^13^C-NMR (125 MHz, CDCl_3_) δ 159.8, 154.2, 140.2, 138.5, 133.6, 132.7, 130.3, 125.6, 123.3, 121.8, 121.0, 120.6, 118.3, 70.8, 70.0, 58.9, 58.0, 53.8, 44.5, 36.1, 29.6, 21.9 ppm.

*6-(Pyridin-3-yl)-N-((tetrahydro-2H-pyran-4-yl)methyl)imidazo[1,5-a]pyridine-3-carboxamide* (**15**). The title compound was obtained according to general procedure (A) using lithium 6-(pyridin-3-yl)imidazo[1,5-a]pyridine-3-carboxylate **30** (1.35 mmol) and 1-(oxan-4-yl)methanamine (1.35 mmol). Yield = 9% (41 mg). HRMS (ESI): calculated 337.1659 [M + H]^+^, found 337.1657 [M + H]^+^. ^1^H-NMR (400 MHz, MeOD) δ 9.72 (s, 1H), 8.90–8.89 (m, 1H), 8.60 (dd, *J* = 4.9, 1.5 Hz, 1H), 8.18 (ddd, *J* = 8.0, 2.3, 1.6 Hz, 1H), 7.85 (dd, *J* = 9.4, 1.0 Hz, 1H), 7.61–7.57 (m, 2H), 7.41 (dd, *J* = 9.4, 1.6 Hz, 1H), 3.96 (dd, *J* = 11.4, 2.7 Hz, 2H), 3.42 (td, *J* = 11.9, 2.1 Hz, 2H), 3.35 (d, *J* = 6.9 Hz, 2H), 1.99–1.87 (m, 1H), 1.75–1.71 (m, 2H), 1.45–1.33 ppm (m, 2H).

*6-(5-Isopropoxypyridin-3-yl)-N-((tetrahydro-2H-pyran-4-yl)methyl)imidazo[1,5-a]pyridine-3-carboxamide* (**16**). The title compound was obtained according to general procedure (A) using lithium 6-{5-[(propan-2-yl)oxy]pyridin-3-yl}imidazo[1,5-a]pyridine-3-carboxylate **31** (0.43 mmol) and 1-(oxan-4-yl)methanamine (0.43 mmol). Yield = 22% (37 mg). HRMS (ESI): calculated 395.2078 [M + H]^+^, found 395.2075 [M + H]^+^. ^1^H-NMR (400 MHz, MeOD) δ 9.66 (s, 1H), 8.39 (d, *J* = 1.5 Hz, 1H), 8.23 (d, *J* = 2.5 Hz, 1H), 7.80 (dd, *J* = 9.4, 1.0 Hz, 1H), 7.65–7.63 (m, 1H), 7.56 (d, *J* = 0.7 Hz, 1H), 7.35 (dd, *J* = 9.4, 1.5 Hz, 1H), 4.83–4.79 (m, 1H), 3.95 (dd, *J* = 11.4, 2.8 Hz, 2H), 3.44–3.38 (m, 2H), 3.33 (d, *J* = 6.9 Hz, 2H), 1.97–1.88 (m, 1H), 1.74–1.70 (m, 2H), 1.42–1.32 ppm (m, 8H). 

*6-(4-(Hydroxymethyl)pyridin-3-yl)-N-((tetrahydro-2H-pyran-4-yl)methyl)imidazo[1,5-a]pyridine-3-carboxamide* (**17**). The title compound was obtained according to general procedure (A) using lithium 6-(4-(hydroxymethyl)pyridin-3-yl)imidazo[1,5-a]pyridine-3-carboxylate **32** (0.35 mmol) and 1-(oxan-4-yl)methanamine (0.42 mmol). Yield = 45% (34 mg). HRMS (ESI): calculated 367.1764 [M + H]^+^, found 367.1763 [M + H]^+^. ^1^H-NMR (400 MHz, MeOD) δ 9.43 (bs, 1H), 8.61 (d, *J* = 5.2 Hz, 1H), 8.49 (s, 1H), 7.81 (dd, *J* = 9.3, 1.0 Hz, 1H), 7.74 (d, *J* = 5.2 Hz, 1H), 7.62 (s, 1H), 7.11 (dd, *J* = 9.3, 1.5 Hz, 1H), 4.66 (s, 2H), 3.95 (dd, *J* = 11.4, 2.7 Hz, 2H), 3.41 (td, *J* = 11.9, 2.1 Hz, 2H), 3.34 (d, *J* = 4.8 Hz, 2H), 1.97–1.85 (m, 1H), 1.74–1.68 (m, 2H), 1.42–1.30 ppm (m, 2H).

*6-(2,3-Difluorophenyl)-N-((tetrahydro-2H-pyran-4-yl)methyl)imidazo[1,5-a]pyridine-3-carboxamide* (**18**). The title compound was obtained according to general procedure (A) using lithium 6-(2,3-difluorophenyl)imidazo[1,5-a]pyridine-3-carboxylate **33** (1.05 mmol) and 1-(oxan-4-yl)methanamine (1.05 mmol). Yield = 15% (59 mg). HRMS (ESI): calculated 372.1518 [M + H]^+^, found 372.1520 [M + H]^+^. ^1^H-NMR (500 MHz, DMSO-d_6_) δ 9.66 (s, 1H), 8.69 (t, 1H, *J* = 10.0Hz), 7.90 (d, 1H, *J* = 9.3 Hz), 7.66 (s, 1H), 7.56–7.50 (m, 1H) 7.49–7.46 (m, 1H), 7.39–7.35 (m, 1H), 7.30 (d, 1H, *J* = 10.0Hz), 3.84 (br dd, 2H, *J* = 2.4, 11.3 Hz), 3.25 (t, 2H, *J* = 10.0Hz), 3.21 (t, 2H, *J* = 10.0Hz), 1.89–1.81 (m, 1H), 1.58 (br d, 2H, *J* = 15.0Hz), 1.25–1.17 ppm (m, 2H). ^1^H^13^C-NMR (125 MHz, DMSO-d_6_) δ 159.8, 150.7, 147.8, 132.3, 130.5, 127.3, 126.2, 125.9, 124.6, 123.0, 121.8, 121.1, 118.8, 117.9, 67.2, 44.5, 35.5, 31.0 ppm.

### 3.2. X-ray Crystallography

#### 3.2.1. Crystals of GSK-3β in Complex with Ligand **2**

Prior to crystallization trials, the protein (protein buffer: 20 mM MOPS pH 7.0, 93.5 mM NaCl,10% Glycerin, 1 mM DTT, 1xPIC) was diluted to 4.0 mg/mL and supplemented with 2 mM of the 1H-indazole-3-carboxamide inhibitor **2**. Crystals of GSK-3β/Compound **2** were grown in the following conditions: 12–20% *v/v* PEG 8000, 0.13 M NaCl, 0.10 M TrisAcetate pH 7.5–8.0, 5 mM TCEP at 4 °C. Crystals appeared within 3 to 7 days in hanging drop vapor diffusion setups and were cryo-protected with glycerol, and vitrified in liquid nitrogen. Data collection and model statistics of the crystal structure of GSK-3β in complex with ligand **2** are shown in the Supplementary Materials. The X-ray diffraction data have been collected from complex crystals of human GSK-3β with the ligand **2** at the SWISS LIGHT SOURCE (SLS, Villigen, Switzerland) using cryogenic conditions. The crystals belong to space group C 2 2 2_1_. Data were processed to 2.08 Å resolution using the programs XDS and XSCALE [46]. The phase information necessary to determine and analyze the structure of GSK-3β in complex with ligand **2** was obtained by molecular replacement using a previously solved GSK-3β structure as a search model. CORINA was used for ligand parameterization [47]. Subsequent model building and refinement was performed with the software packages CCP4 and COOT [48,49]. For the measure of the free R-factor, a measure to cross-validate correctness of the final model, about 5.9% of measured reflections were excluded from the refinement procedure. Several rounds of manual model building in COOT and bulk solvent correction, positional, B-factor, and TLS refinement using REFMAC yielded the final model [48,49]. The atomic coordinates and structure factors have been deposited in the Protein Data Bank (PDB entry 6Y9R).

#### 3.2.2. Crystals of GSK-3β in Complex with Ligand **16**

Prior to crystallization trials, the protein (protein buffer: 20 mM MOPS pH 7.0, 93.5 mM NaCl,10% Glycerin, 1mM DTT, 1xPIC) was diluted to 4.0 mg/mL and supplemented with 1 mM of the imidazo[1,5-a]pyridine-3-carboxamide inhibitor **16**. Crystals of GSK-3β/compound **16** were grown in the following condition: 20–28% PEG 8000, 0.13 M NaCl, 0.10 M TrisAcetate pH 7.75–8.25 at 4 °C. Crystals appeared after 2 to 5 days in hanging drop vapor diffusion setups, were also cryo-protected with glycerol, and vitrified in liquid nitrogen. Data collection and model statistics of the crystal structure of GSK-3β in complex with ligand **16** are shown in the Supplementary Materials. The X-ray diffraction data have been collected from complex crystals of human GSK-3β with the ligand **16** at the SWISS LIGHT SOURCE (SLS, Villigen, Switzerland) using cryogenic conditions. The crystals belong to space group P 2_1_. Data were processed to 2.03 Å resolution using the programs XDS and XSCALE [46]. The phase information necessary to determine and analyze the structure of GSK-3β in complex with ligand **16** was obtained by molecular replacement using a previously solved GSK-3β structure as a search model. CORINA was used for ligand parameterization [47]. Subsequent model building and refinement were performed with the software packages CCP4 and COOT [48,49]. For the measure of the free R-factor, a measure to cross-validate correctness of the final model, about 3.3% of measured reflections were excluded from the refinement procedure. Several rounds of manual model building in COOT and bulk solvent correction, positional, B-factor, and TLS refinement using REFMAC yielded the final model [48,49]. The atomic coordinates and structure factors have been deposited in the Protein Data Bank, www.pdb.org (PDB entry 6Y9S).

### 3.3. MDCK-MDR1 Cell Line Assay

MDCK-MDR1 cells were obtained from the NIH (Rockville, MD, USA). Cells were seeded onto Millipore Multiscreen Transwell plates at 3.4 × 105 cells/cm2 and cultured in DMEM. Cell culture and assay incubations were carried out at 37 °C in an atmosphere of 5% CO2 with a relative humidity of 95%. The monolayers were prepared by rinsing both apical and basolateral surfaces twice with Hanks Balanced Salt Solution (HBSS) at the desired pH warmed to 37 °C. Cells were then incubated with HBSS at the desired pH in both apical and basolateral compartments for 40 min to stabilize physiological parameters. The dosing solutions were prepared by diluting test compound with assay buffer to give a final test compound concentration of 10 μM (final DMSO concentration of 1% *v/v*). Analytical standards were prepared from test compound DMSO dilutions and transferred to buffer, maintaining a 1% *v/v* DMSO concentration. Assay buffer was composed of supplemented HBSS pH 7.4. For assessment of A–B permeability, HBSS was removed from the apical compartment and replaced with test compound dosing solution. The apical compartment insert was then placed into a companion plate containing fresh buffer (containing 1% *v/v* DMSO). For assessment of B–A permeability, HBSS was removed from the companion plate and replaced with test compound dosing solution. Fresh buffer (containing 1% *v/v* DMSO) was added to the apical compartment insert, which was then placed into the companion plate. At 60 min, the apical compartment inserts and the companion plates were separated and apical and basolateral samples diluted for analysis. Test compound permeability was assessed in duplicate. Two control compounds were screened alongside the test compounds, propranolol (highly permeable), and prazosin (a substrate for P-gp). Test and control compounds were quantified by LC-MS/MS cassette analysis using an 8-point calibration with appropriate dilution of the samples. Cyprotex generic analytical conditions were used. The starting concentration (C_0_) was determined from the dosing solution and the experimental recovery calculated from C_0_ and both apical and basolateral compartment concentrations. The permeability coefficient (P_app_) for each compound was calculated from the following Equation (1):

(1) $$P_{\mathrm{app}}=\left(\frac{\mathrm{dQ}/\mathrm{dt}}{C_{0}\times A}\right),$$

where dQ/dt is the rate of permeation of the drug across the cells, C_0_ is the donor compartment concentration at time zero, and A is the area of the cell monolayer. C_0_ was obtained from analysis of the dosing solution. For bi-directional experiments, ER was calculated from mean A–B and B–A data. This was derived from Equation (2):

(2) $$\mathrm{ER}=\left(\frac{P_{\mathrm{app}\left(B–A\right)}}{P_{\mathrm{app}\left(A–B\right)}}\right).$$

ER value greater than two indicates that drug efflux is occurring.

### 3.4. Ligand Docking

The Schrödinger suite was used for all calculations. Ligands were converted to 3D structures and prepared with LigPrep tool using default setting [50]. Overall, the most likely tautomers and protonation states were considered for each ligand. Next, a conformational search was carried out using ConfGen resulting in the generation of 10 conformers per ligand [51]. This represented the input dataset for Glide SP docking [30,31,52]. The coordinates of the crystal structure of GSK-3β in complex with **2** (PDB entry 6Y9R) were used. The protein was prepared using Maestro Protein Preparation Wizard [53]. Hydrogen atoms were added, and water molecules were removed from the protein structure. The default setting was used for receptor grid preparation. The binding site was specified as a box of 10 × 10 × 10 Å^3^ centered on the centroid of the co-crystallized inhibitor. In addition, we treated thiol and hydroxyl groups of Tyr134, Thr138, and Cys199 as rotatable (flexible) groups. Three H-bond constraints were defined being the carbonyl oxygen of the hinge residues Asp133 and Val135, and the hydrogen of the N-H group of Val135. In Glide SP docking, we required that at least one such H-bond crucial for compound activity was formed. We also used the enhanced sampling option to enhance the sampling of conformational space by adding variations on the input structure to the conformational search and included aromatic ligand hydrogen atoms as donors. Visual inspection of the docking results was carried out enabling the picking of one pose per ligand as a starting point for further studies. 

A rigid-body superposition of INDZ vs. IMID 1 and IMID 2 analogues within each triplets was performed using Maestro software [54], and RMSD was computed (Table 2). The common substructure was defined considering all heavy atoms with the exception of the ones of the bicyclic system that are different moving from INDZ to IMID 1 and IMID 2 analogues. RMSD was computed without changing structure positions.

Figure 3 and Figure 5 were prepared with PyMOL 1.8.6.2 [55]. Crystal structure of **16** in complex of GSK-3β (Figure 5) was prepared using a default setting of the Maestro Protein Preparation Wizard tool [53].

### 3.5. MD Simulations

The selected binding poses of **1**–**18** within the ATP binding site of GSK-3β were processed in MD simulation using the graphics processing unit (GPU) tool of the Desmond MD package as supplied with the Schrödinger 2019-1 suite [32,56]. The Desmond System Builder was used to create a fully solvated system with simple point charge (SPC) waters and a cubic solvation box with a 10 Å buffer around the original complex. The system was electrostatically neutralized by adding Na^+^ ions in defined positions by using the ion placement option. Default parameters of OPLS-3e force field were used. The prepared systems included approximately 58,900 atoms. The MD simulation protocol followed an extended relaxation schedule consisting of the following steps:

1. 100 ps, constant–volume ensemble (NVT) (T = 10 K); 50 kcal/mol/Å^2^ restraints on the solute heavy atoms;
2. 120 ps, NVT (T = 10 K); 50 kcal/mol/Å^2^ restraints on the solute heavy atoms;
3. 120 ps, constant–pressure ensemble (NPT) (T = 10K, P = 1 atm); 50 kcal/mol/Å^2^ restraints on the solute heavy atoms;
4. 120 ps, NPT (T = 300 K, P = 1 atm); 50 kcal/mol/Å^2^ restraints on the solute heavy atoms;
5. 2400 ps, NPT (T = 300 K, P = 1 atm); unrestrained;
6a. Derivatives **2**, **8** and **14**: 500 ns, NPT (T = 300 K, P = 1 atm); unrestrained;
6b. Remaining compounds: 100 ns, NPT (T = 300 K, P = 1 atm); unrestrained.

From 6a and 6b MD production step, 5000 and 1000 frames were respectively stored. The analysis of the MD trajectory was basically performed with the Schrödinger suite. RMSD and RMSF of both proteins and ligands, ligand–protein contacts along the simulations were calculated with the Simulation Interaction Diagram (SID) tool implemented in the Desmond MD package. MD simulations were visual inspected with VMD 1.9.4 [24]. By default, the deep H-bond involving C-Ha and Asp133 is not detected with the SID tool. Therefore, distances and angles of such interaction in IMID 1 and IMID 2 simulations were monitored and calculated via VMD 1.9.4.

For the comparison of 500 and 100 ns MD simulations of **2**, **8**, and **14**, raw data from the SID output were processed using bash command lines and Knime workflows [57]. In Figure S1, the interactions were categorized based on the definition of the Schrödinger tool. The stacked bar charts were normalized over the course of the trajectory. Values over 1.0 were possible as some protein residues may make multiple contacts of same subtype with the ligand. Figure S1 was realized with Tibco Spotfire 7.11.1 (Palo Alto, CA, USA). 

The percentages of interaction of the outer and central H-bond across each MD trajectory (Table S1) and of other contacts discussed in the Results and Discussion section were basically extracted from the SID outcomes. 

The distances of the outer, central, and deep H-bonds along all the simulations were calculated with VMD 1.9.4, combined with Knime workflow and plotted in Figure 6 with Tibco Spotfire 7.11.1 (Palo Alto, CA, USA). Figure 7 with the angles of the deep H-bond interactions was created with the same software.

By way of illustration, snapshots of some MD trajectories were extracted using VMD 1.9.4 and reported in Figure S3. For the timeline representation of Figure S4, the interaction counts were basically extracted from the SID results. In order table sto count the deep H-bond interactions in IMID 1 and IMID 2 simulations, the SID geometric criteria for ligand-protein H-bond were applied to the distance and angle values collected with VMD 1.9.4. Knime workflow, and bash command lines were used to combine all these results in the timeline representation. 

The average binding free energy (ΔG_bind) based on MM-GBSA was calculated using the thermal_mmgbsa.py script on 100 frames extracted from each production trajectory [35,36] (Table 3). 

### 3.6. QM Studies

The predictions of hydrogen bond acidity are based on the work of Kenny [40]. The Vα(r) descriptor represents the value of the electrostatic potential at a distance r along the X-H vector from the hydrogen atom. The 18 SP docking poses were used for the Vα(r) calculation as implemented in Jaguar software [39]. B3LYP/6-31G** level of theory was used with r = 0.55 Å. 

### 3.7. NMR Study

NMR experiments were performed in deuterated DMSO and CDCl_3_ solutions and recorded with a Bruker Avance spectrometer operating at 500 MHz for ^1^H and 125 MHz for ^13^C, equipped with a 5 mm inverse broad-band probe and z-axis gradients. 

The complete NMR spectra assignment of the three compounds was performed in CDCl_3_ solutions at 298 K. COSY experiments were acquired with a spectral window of 15 × 15 ppm (carrier frequency at 4.5 ppm) using 2048 × 256 data points, 16 transients, with a relaxation delays of 2 s. NOESY experiments were acquired with a spectral window of 15 × 15 ppm (carrier frequency at 4.5 ppm) using 4096 × 256 data points, 32 transients, with a relaxation delay of 2 s and a mixing time of 600 ms. ^1^H-^13^C HSQC experiments were acquired with a spectral window of 15 ppm × 200 ppm (carrier frequencies at 6.5 and 100 ppm) using 2048 × 256 data points, 16 transients with relaxation delays of 2 s. ^1^H-^13^C HMBC experiments were acquired with a spectral window of 15 ppm × 220 ppm (carrier frequency at 6.5 and 110 ppm) using 4096 × 512 data points, 32 transients with relaxation delays of 2 s. All data were processed with TopSpin using a sine squared window function for each dimension of the 2D data. 

The variations of chemical shift (Δδ) for protons of interest were calculated from the difference of the chemical shift in DMSO-d6 and in CDCl_3_, detected in the ^1^H-NMR spectra at 298 K.

For T_coeff_ determination, ^1^H-NMR spectra of the three compounds were performed in deuterated DMSO at five different temperatures: 298 K, 303 K, 308 K, 313 K, and 318 K.

The empirical estimation of the H-bonding strength was performed using the following equation, based on the Δδ values obtained from ^1^H-NMR spectra:

(3) $$E_{HB}\left(\Delta \delta \right)=\Delta \delta +\left(0.4\pm 0.2\right)\;kcal/mol.$$

## 4. Conclusions

In this study, novel derivatives around the lead series core scaffold were investigated by means of ligand docking and MD simulations. The novel IMID derivatives turned out to be GSK-3β inhibitors in the nanomolar scale with a potency drop compared to the previous INDZ series but improved CNS permeability. Interestingly, the in silico-derived models of the binding mode generated during the design process well suited the crystallographic data (PDB entry 6Y9S). In order to rationalize SAR, computational tools were also employed retrospectively. In particular, MD and QM-based calculations shed light on the differences of the H-bond interactions between the core scaffolds and the hinge residues moving from INDZ to IMID 1 and IMID 2 derivatives. The pharmacological profile of IMID 1 and IMID 2 derivatives was associated to the lower acidity of the C-Ha versus N-Ha group of the core scaffolds. NMR-based studies were also carried out to substantiate this hypothesis. Overall, our findings provided adequate evidence that low C-Ha acidity and consequent unfavorable geometric rearrangement affected H-bond donating abilities so reducing the affinity for GSK-3β enzyme. This represented significant structural activity insight into our GSK-3β inhibitor design and demonstrated the pivotal role of computational approaches properly supported by experimental proofs in the different steps of the drug discovery program. In the future, prospective application of the aforementioned in silico tools may lead to further optimized classes of GSK-3β inhibitors. 

## Figures and Tables

**Table 1:** SAR exploration of INDZ, IMID 1, and IMID 2 derivatives.

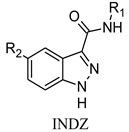				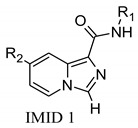				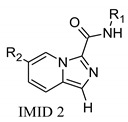			
Cmpd	Core	R_1_	R_2_	GSK-3βIC_50_ (μM) ^1^	P_app_ (10^−6^ cm/s) ^2^	ER ^2^	cLogD	TPSA ^3^	pK_a_^3, 4^	HBD ^3^	MW ^3^
**1**	INDZ	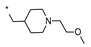	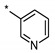	0.031	-	-	1.02	83.14	8.40	2	393.48
**7**	IMID 1			0.410	-	-	0.50	71.76	8.40	1	393.48
**13**	IMID 2			0.370	1.260	52.10	0.75	71.76	8.41	1	393.48
**2**	INDZ	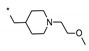	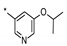	0.009	0.486	43.60	1.59	92.37	8.40	2	451.56
**8**	IMID 1			0.120	0.522	50.80	1.09	80.99	8.40	1	451.56
**14**	IMID 2			0.230	3.100	18.10	1.42	80.99	8.41	1	451.56
**3**	INDZ	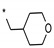	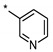	0.022	15.800	2.72	1.91	79.90	4.41	2	336.39
**9**	IMID 1			0.180	-	-	1.39	68.52	2.72	1	336.39
**15**	IMID 2			0.091	32.100	1.57	1.72	68.52	3.29	1	336.39
**4**	INDZ	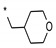	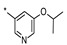	0.004	1.490	56.10	2.56	89.13	3.83	2	394.47
**10**	IMID 1			0.200	4.930	15.50	2.30	77.75	2.08	1	394.47
**16**	IMID 2			0.072	-	-	2.63	77.75	2.67	1	394.47
**5**	INDZ	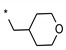	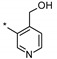	0.033	-	-	1.06	100.10	4.23	3	366.41
**11**	IMID 1			1.400	-	-	0.60	88.75	2.54	2	366.41
**17**	IMID 2			0.910	-	-	1.02	88.75	3.11	2	366.41
**6**	INDZ	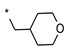	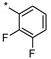	0.014	13.300	4.17	3.10	67.01	2.09	2	371.38
**12**	IMID 1			0.280	41.900	1.09	2.49	55.63	1.34	1	371.38
**18**	IMID 2			0.240	37.500	0.856	2.83	55.63	1.21	1	371.38

^1^ IC_50_ values were calculated from data points obtained as average of duplicate wells. ^2^ In vitro permeability experiments were performed on MDCK-MDR1 cell line at 10 μM. P_app_ values were calculated from data points obtained as average of duplicate wells. ER = Efflux Ratio. ^3^ cLogD (pH = 7.4), TPSA and MW values were calculated using ACD/Percepta 2017.2. ^4^ pK_a_ refers to the most basic center.

**Table 2:** Glide SP docking score of the selected poses of derivatives **1**–**18**. RMSD of the docking poses within each triplet (INDZ vs. IMID 1 and IMID 2 analogues). ^1^ RMSD based on the core and carboxamide portion. ^2^ RMSD based on the whole molecule. Ref. stands for reference compound.

ID	Core	Glide SP Docking Score (kcal/mol)	RMSD Core + Carboxamide (Å) ^1^	RMSD (Å) ^2^
**1**	INDZ	−8.48	Ref.	Ref.
**7**	IMID 1	−8.58	0.44	1.24
**13**	IMID 2	−8.69	0.34	0.76
**2**	INDZ	−8.77	Ref.	Ref.
**8**	IMID 1	−7.94	0.45	1.36
**14**	IMID 2	−8.44	0.43	0.92
**3**	INDZ	−8.88	Ref.	Ref.
**9**	IMID 1	−8.28	0.15	0.70
**15**	IMID 2	−8.25	0.19	0.39
**4**	INDZ	−8.44	Ref.	Ref.
**10**	IMID 1	−7.91	0.18	1.00
**16**	IMID 2	−7.72	0.15	1.51
**5**	INDZ	−8.16	Ref.	Ref.
**11**	IMID 1	−8.51	0.22	0.72
**17**	IMID 2	−8.61	0.34	0.28
**6**	INDZ	−7.96	Ref.	Ref.
**12**	IMID 1	−8.14	0.26	1.08
**18**	IMID 2	−8.95	0.67	1.42

**Table 3:** MM-GBSA ΔG_bind average from each MD simulation (100 snapshots per compound).

Compound	Core	MM-GBSA ΔG_bind (kcal/mol)
**1**	INDZ	−63.69
**7**	IMID 1	−58.34
**13**	IMID 2	−56.96
**2**	INDZ	−70.98
**8**	IMID 1	−65.23
**14**	IMID 2	−63.42
**3**	INDZ	−53.88
**9**	IMID 1	−49.25
**15**	IMID 2	−50.19
**4**	INDZ	−59.97
**10**	IMID 1	−57.98
**16**	IMID 2	−58.30
**5**	INDZ	−55.87
**11**	IMID 1	−53.68
**17**	IMID 2	−51.97
**6**	INDZ	−55.56
**12**	IMID 1	−51.02
**18**	IMID 2	−52.11

**Table 4:** Vα(r) values (kcal/mol) of the core Ha and carboxamide Hb.

Compound	Core	Vα(r)	
		Ha	Hb
**1**	INDZ	256.37	245.08
**7**	IMID1	211.13	234.78
**13**	IMID2	198.73	247.25
**2**	INDZ	258.51	248.31
**8**	IMID1	212.36	237.47
**14**	IMID2	198.57	235.57
**3**	INDZ	219.28	191.94
**9**	IMID1	174.58	184.25
**15**	IMID2	160.91	185.69
**4**	INDZ	219.30	192.82
**10**	IMID1	173.32	183.04
**16**	IMID2	159.89	184.80
**5**	INDZ	218.93	191.53
**11**	IMID1	176.54	184.68
**17**	IMID2	160.85	186.16
**6**	INDZ	218.71	192.92
**12**	IMID1	175.11	183.15
**18**	IMID2	160.20	185.42

**Table 5:** Chemical shifts of Ha and Hb observed in the ^1^H-NMR spectra of **2**, **8** and **14** derivatives (0.3 mM) in CDCl_3_ and DMSO-d_6_.

**Ha**				
**Compound**	**Core**	**δ (ppm) in DMSO-d_6_**	**δ (ppm) in CDCl_3_**	**Δδ (ppm)**
**2**	INDZ	13.7	10.3	**3.4**
**8**	IMID 1	8.5	8.0	**0.5**
**14**	IMID 2	7.6	7.5	**0.1**
**Hb**				
**Compound**	**Core**	**δ (ppm) in DMSO-d_6_**	**δ (ppm) in CDCl_3_**	**Δδ (ppm)**
**2**	INDZ	8.4	7.2	**1.2**
**8**	IMID 1	8.1	7.3	**0.8**
**14**	IMID 2	8.6	7.5	**1.1**

**Table 6:** Temperature coefficients (T_coeff_) obtained for N-Ha proton of compound **2** and N-Hb protons of compounds **2**, **8**, and **14**.

T_coeff_ (ppb/K)			
Hydrogen	INDZ (2)	IMID 1 (8)	IMID 2 (14)
**Ha**	−4.6	-	-
**Hb**	−6.8	−6.3	−6.6

**Table 7:** Energy of the H-bonds E_HB_ (kcal/mol) estimated using Equation (1) for compounds **2-8-14**.

*E_HB_* ± 0.2 (kcal/mol)			
Hydrogen	INDZ (2)	IMID 1 (8)	IMID 2 (14)
**Ha**	3.7	-	-
**Hb**	1.7	1.2	1.5

## Supplementary Materials

The following are available online, Figure S1: Ligand–Protein interactions monitored throughout 100 ns (a) and 500 ns (b) simulations, Figure S2: Ligand RMSF from each MD simulation, Figure S3: representative snapshots of MD simulations, Figure S4: Timeline representation of the interactions along 100 ns simulations for each compound, Figures S5–S7: ^1^H-NMR spectrum (500 MHz) of compounds **2**, **8**, and **14** in CDCl_3_ and DMSO-d6 at 298 K. Table S1: Percentage of interaction of the outer and central H-bond across each MD trajectory, Table S2: Data collection and model statistics of crystal structure of **2** in complex with GSK-3β enzyme (PDB entry 6Y9R), Table S3: Data collection and model statistics of crystal structure of **16** in complex with GSK-3β enzyme (PDB entry 6Y9S), HNMR spectra of final compounds, 2D NMR of compounds **2**, **8**, and **14**.
