# Supplementary material for: Discovery of Novel Imidazopyridine GSK-3β Inhibitors Supported by Computational Approaches
Source: Molecules. 2020 May 5;25(9):2163. doi: 10.3390/molecules25092163 (PMC7248956; doi:10.3390/molecules25092163)

molecules

Supplementary Materials

Discovery of Novel Imidazopyridine GSK-3β Inhibitors Supported by Computational Approaches

Rosa Buonfiglio, Federica Prati, Martina Bischetti, Claudia Cavarischia, Guido Furlotti, and Rosella Ombrato *

Angelini Pharma S.p.A., Viale Amelia, 70 - 00181 Rome, Italy
* Correspondence: rosella.ombrato@angelinipharma.com

Table of contents **Pg**

Figure S1	S2
Figure S2	S3
Figure S3	S4
Figure S4	S5
Figure S5	S6
Figure S6	S6
Figure S7	S7
Table S1	S7
Table S2	S8
Table S3	S8
HPLC analysis data of final compounds	S10-S21
^{1}HNMR and ^{13}CNMR spectra of final compounds	S22-S31
2D NMR of compounds **2**, **8** and **14**	S32-S37

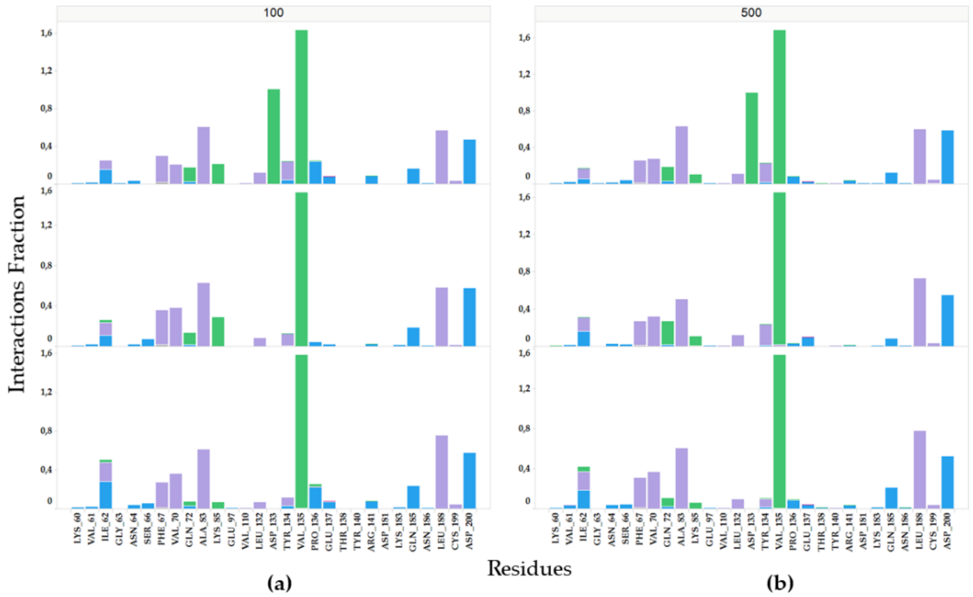

(a) **(b)**

Figure S1. Ligand-Protein interactions monitored throughout 100 ns (**a**) and 500 ns (**b**) simulations. The upper, medium and lower panel correspond to **2**, **8** and **14** complexes. The interactions are categorized as follows: H-bonds (green), Hydrophobic (purple), Ionic (fuchsia), Water Bridges (cyan), Pi-Cation (yellow), Pi-Pi (grey). The stacked bar charts are normalized over the course of the trajectory. The unconventional deep H-bond involving C-Ha and Asp133 is not detected by default in the Simulation Interaction Diagram tool, therefore the interaction fraction of Asp133 approaches zero in **8** and **14** (both 100 and 500 ns timeline) compared to **2**.

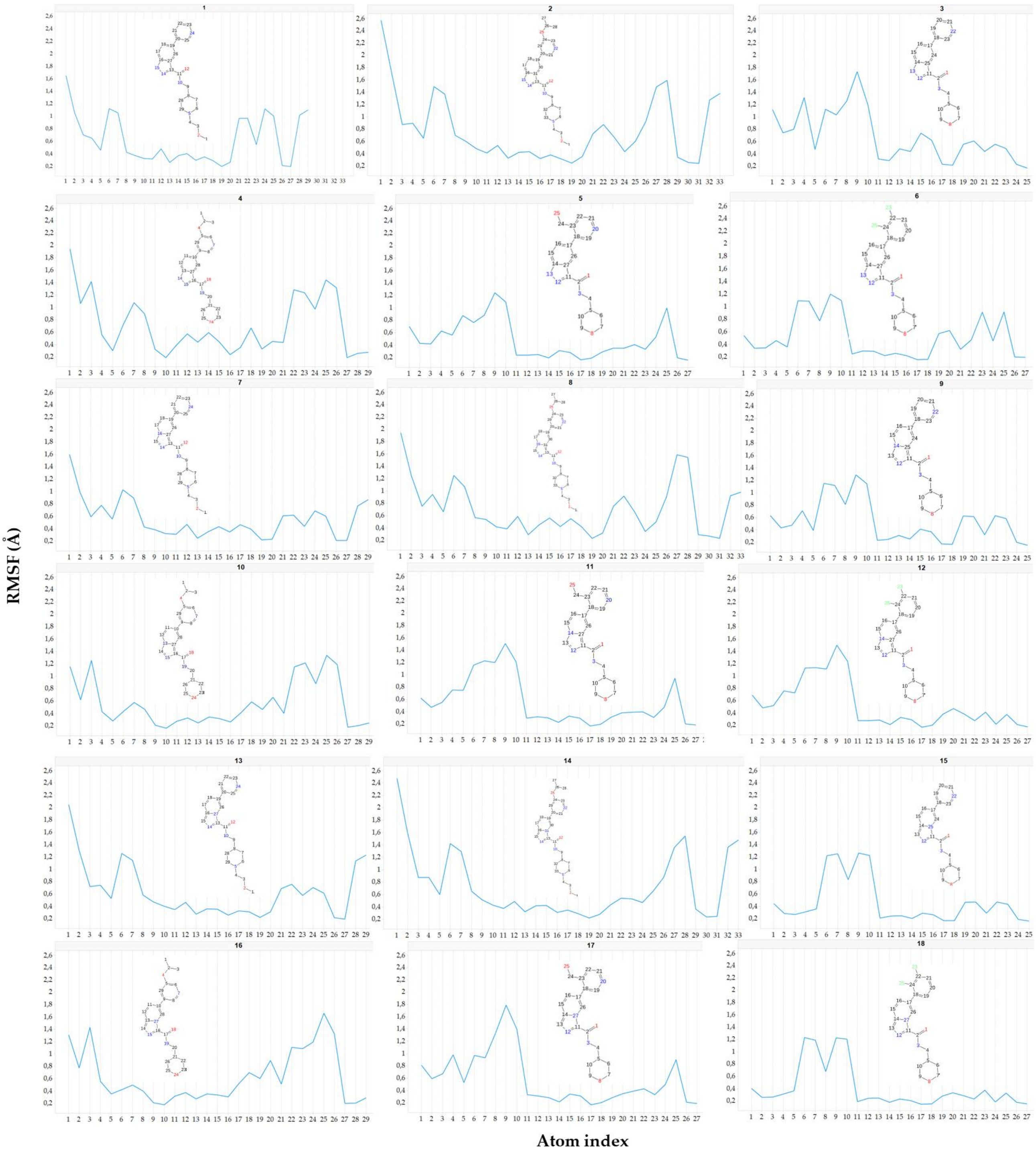

Figure S2. Ligand RMSF from each MD simulation broken down by atom, corresponding to the 2D structure within the plot. Compound ID is reported in the grey row on the top part of each plot.

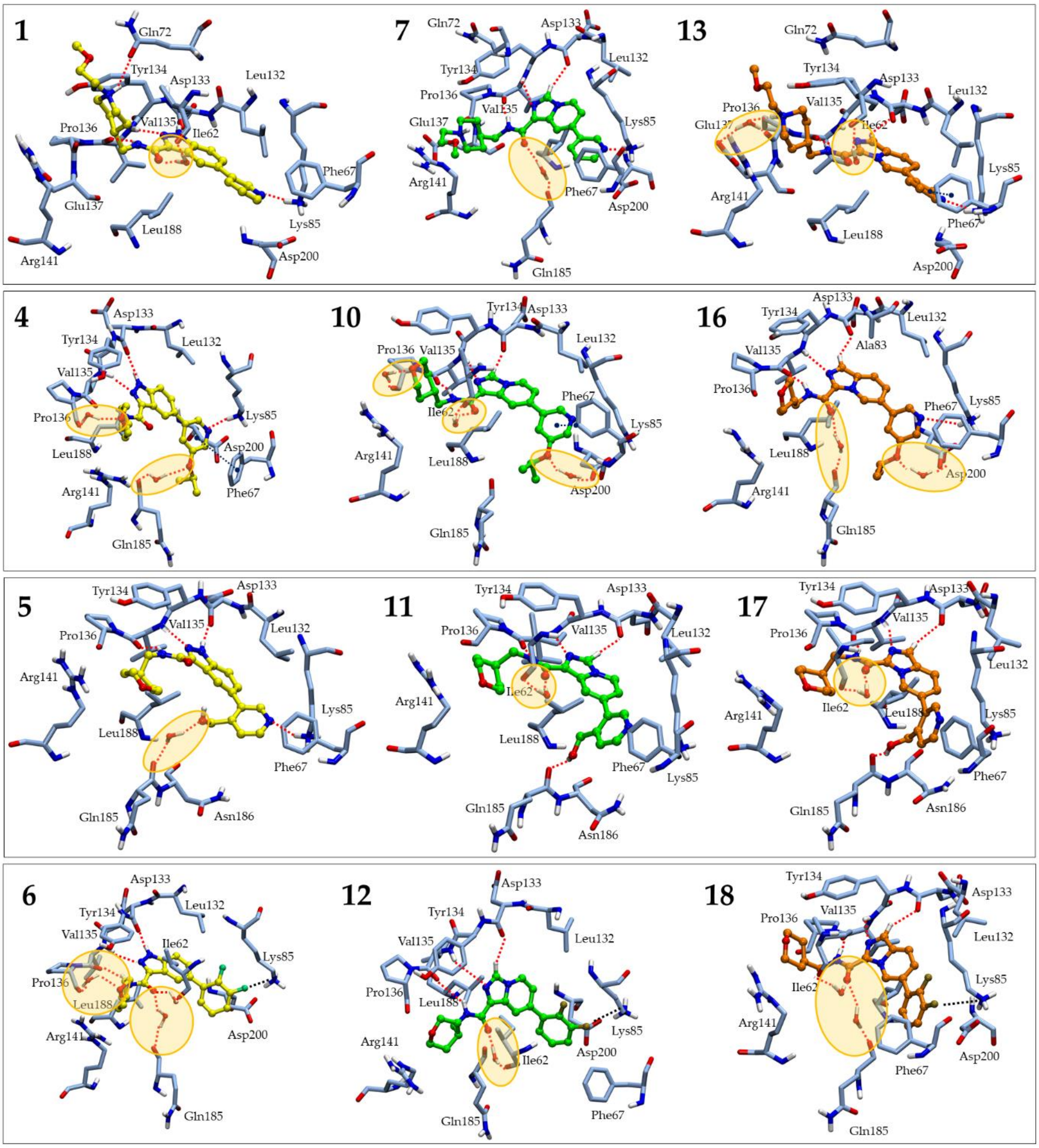

Figure S3. Representative snapshots from MD simulations. INDZ, IMID 1, and IMID 2 derivatives are reported as yellow, green and orange ball and sticks representation, respectively. The most relevant interactions are represented with dashed lines. Water mediated contacts are highlighted with yellow circles.

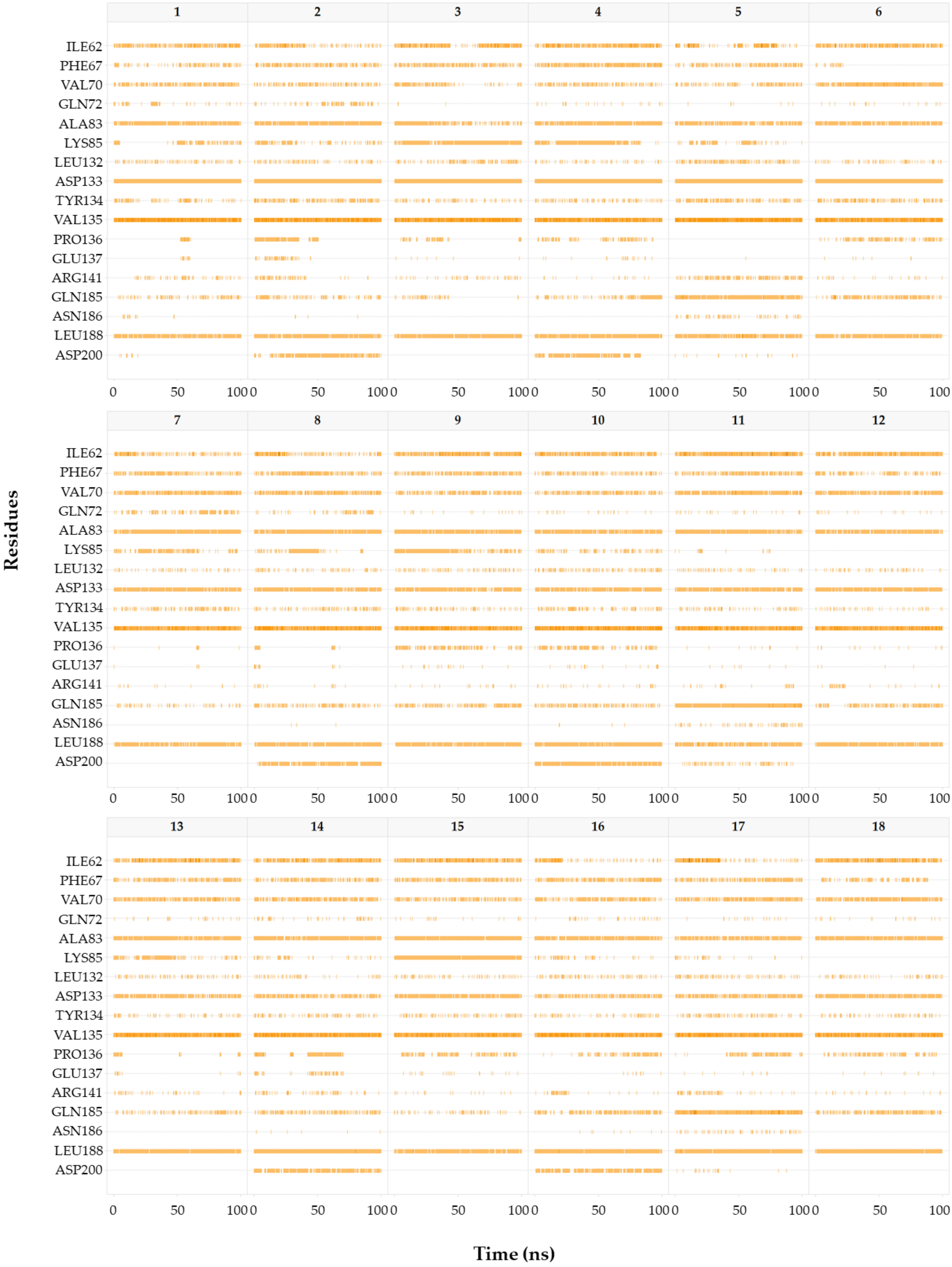

Figure S4. Timeline representation of the interactions along 100 ns simulations for each compound. The top, medium and bottom row refer to **1-6** (INDZ), **7-12** (IMID 1) and **13-18** (IMID 2) derivatives. Residues making more than one specific contact with the ligand are represented by a darker shade of orange.

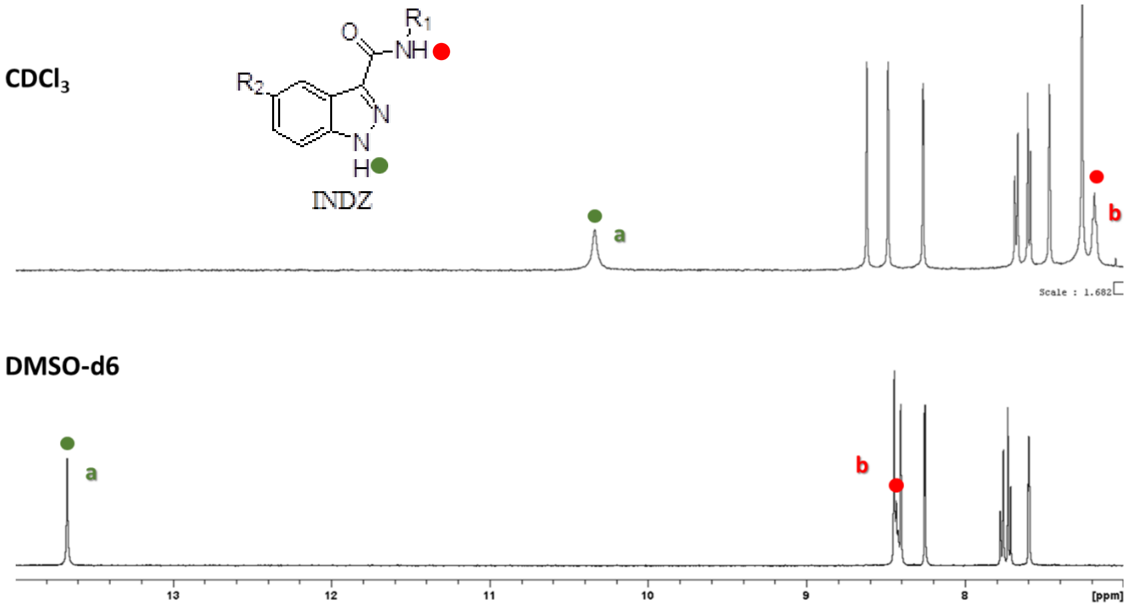

Figure S5. ^{1}H-NMR spectrum (500 MHz) of compound **2** in $CDCl_3$ (upper panel) and DMSO-d6 (lower panel) at 298 K. Ha and Hb are reported as a and b, respectively.

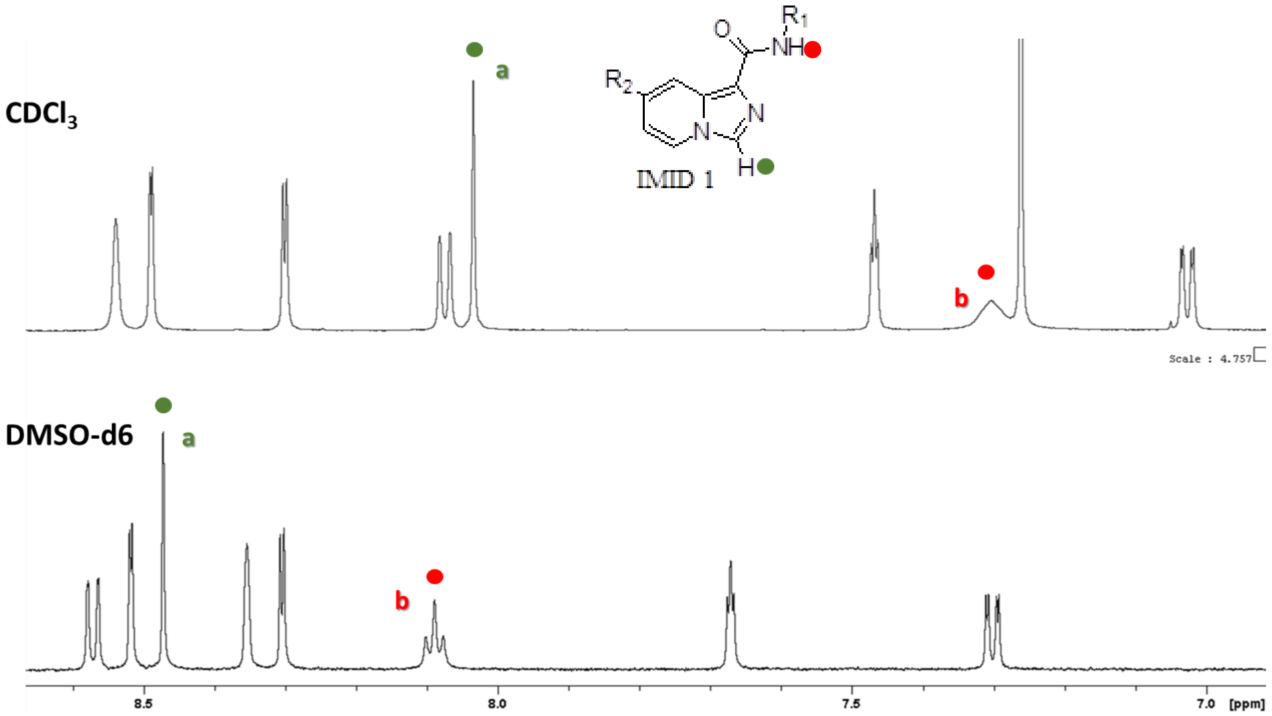

Figure S6. ^{1}H-NMR spectrum (500 MHz) of compound **8** in $CDCl_3$ (upper panel) and DMSO-d6 (lower panel) at 298 K. Ha and Hb are reported as a and b, respectively.

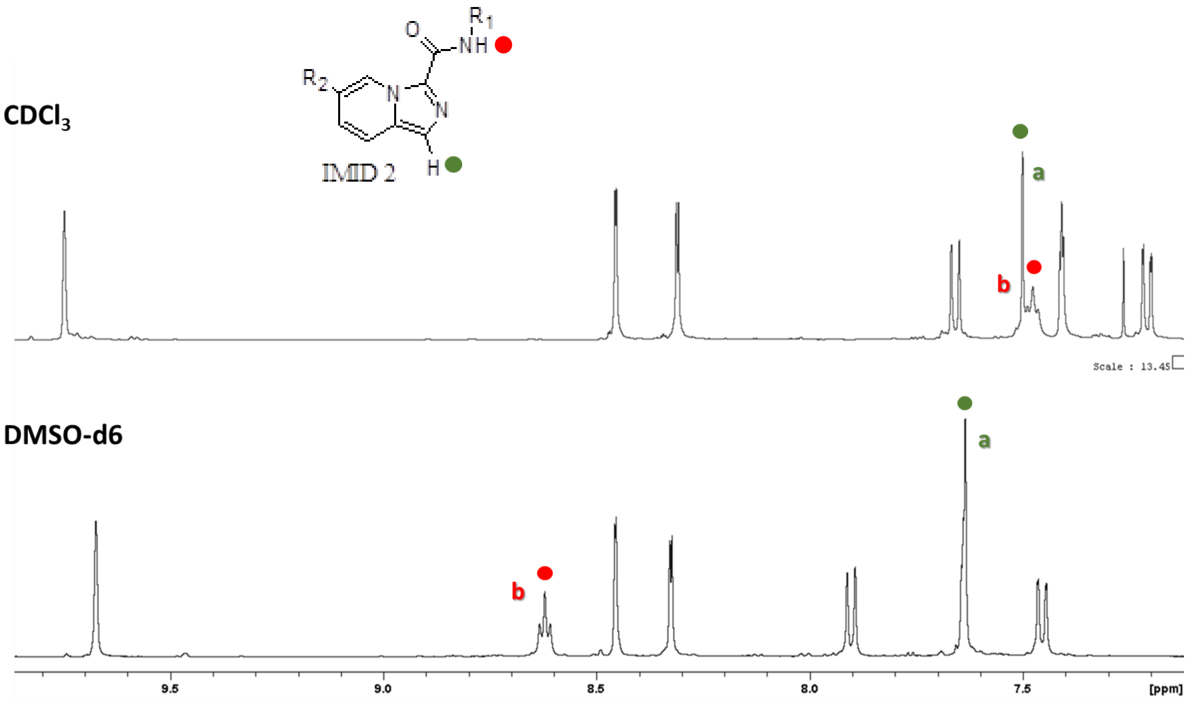

Figure S7. ^{1}H-NMR spectrum (500 MHz) of compound **14** in $CDCl_3$ (upper panel) and DMSO-d6 (lower panel) at 298 K. Ha and Hb are reported as a and b, respectively.

Table S1: Percentage of interaction of the outer and central H-bond across each MD trajectory

Cmpd	Core	Outer H-bond (%)	Central H-bond (%)
**1**	INDZ	98	74
**7**	IMID 1	98	43
**13**	IMID 2	98	55
**2**	INDZ	98	64
**8**	IMID 1	99	52
**14**	IMID 2	99	58
**3**	INDZ	93	70
**9**	IMID 1	97	47
**15**	IMID 2	99	46
**4**	INDZ	94	55
**10**	IMID 1	98	64
**16**	IMID 2	99	61
**5**	INDZ	89	76
**11**	IMID 1	89	45
**17**	IMID 2	94	55
**6**	INDZ	92	58
**12**	IMID 1	91	46
**18**	IMID 2	97	48

Table S2. Data collection and model statistics of crystal structure of **2** in complex with GSK-3β enzyme (PDB entry 6Y9R)

**Ligand**	**2**
X-ray source	PXI/X06SA (SLS)
Wavelength [Å]	1.0000
Detector	EIGER X 16M
Temperature [K]	100
Space group	$C\ 2\ 2\ 2_1$
Cell: a; b; c; [Å]	84.58; 108.29; 103.47
α; β; γ; [°]	90.0; 90.0; 90.0
Resolution [Å]	2.08 (2.33-2.08)
Unique reflections	28265 (7982)
Multiplicity	5.2 (5.2)
Completeness [%]	97.8 (97.2)
R_{sym} [%]	5.5 (43.5)
R_{meas} [%]	6.1 (48.3)
Mean(I)/sd	16.89 (4.23)
Number of reflections (working /test)	26594 / 1671
Rcryst [%]	18.9
Rfree[%]2	22.6
Total number of atoms:	
Protein	2863
Water	133
Ligand	33
Acetate	4
Glycerol	12
Deviation from ideal geometry:	
Bond lengths [Å]	0.008
Bond angles [°]	1.37
Bonded B's [Å2]	4.2
Ramachandran plot:	
Most favoured regions [%]	90.5
Additional allowed regions [%]	9.2
Generously allowed regions [%]	0.0
Disallowed regions [%]	0.3

Table S3. Data collection and model statistics of crystal structure of **16** in complex with GSK-3β enzyme (PDB entry 6Y9S)

**Ligand**	**16**
X-ray source	PXII/X10SA (SLS)
Wavelength [Å]	1.0000
Detector	PILATUS 6M
Temperature [K]	100
Space group	$P\ 2_1$
Cell: a; b; c; [Å]	67.11; 96.19; 67.56
α; β; γ; [◦]	90.0; 103.6; 90.0
Resolution [Å]	2.03 (2.28-2.03)
Unique reflections	50808 (14391)
Multiplicity	2.0 (1.9)
Completeness [%]	94.2 (91.4)
R_{sym} [%]	5.5 (44.8)
R_{meas} [%]	7.4 (59.9)
Mean(I)/sd	9.63 (1.78)
Number of reflections (working /test)	49126 / 1682
Rcryst [%]	20.2
Rfree[%]2	24.9
Total number of atoms:	
Protein	5501
Water	282
Ligand	58
Acetate	12
Deviation from ideal geometry:	
Bond lengths [Å]	0.011
Bond angles [°]	1.51
Bonded B's [Å2]	5.1
Ramachandran plot:	
Most favoured regions [%]	92.3
Additional allowed regions [%]	7.0
Generously allowed regions [%]	0.3
Disallowed regions [%]	0.3

HPLC analysis data of final compounds

Injection Volume	5 uL
Detector	UV (252 nm)
Mobile Phase	CH3CN + 0.01%TFA/Water+0.01%TFA (10:90 v/v)
Temperature	25°C

Column Name	Xbridge C18
Length	15 cm
Diameter	0.46 cm
Particle Size	5 um
Column Flow Rate	2 ml/min

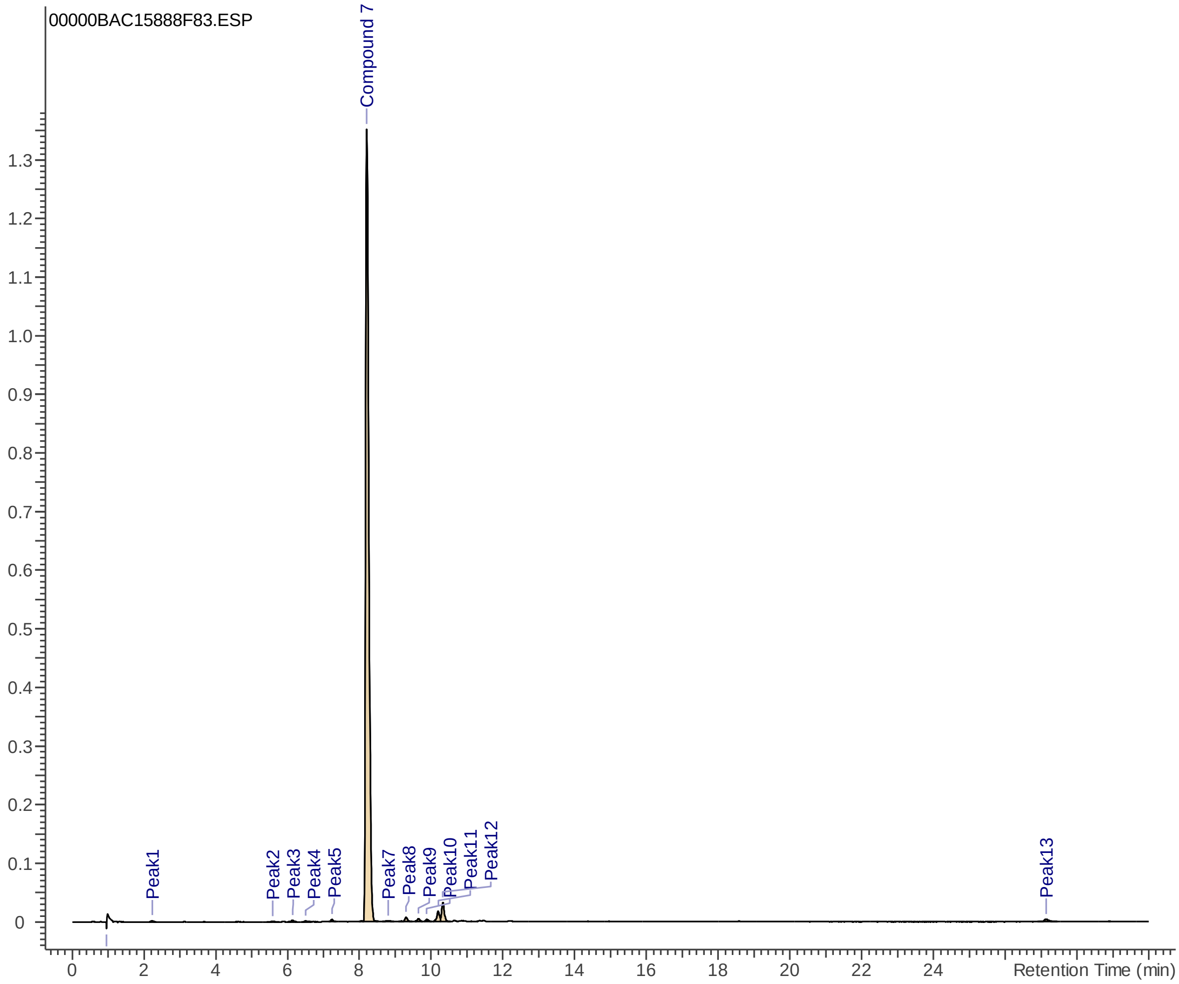

No.	Name	tR	Peak Area (Y units*ms)	Area Percent	Width	Height
1	Peak1	2.23	8.334	0.096	0.121	1.816
2	Peak2	5.58	5.613	0.064	0.128	0.894
3	Peak3	6.14	13.612	0.156	0.115	2.799
4	Peak4	6.50	8.073	0.093	0.125	1.458
5	Peak5	7.23	16.448	0.189	0.116	3.675
6	Compound 7	8.21	8285.923	95.059	0.165	1351.611
7	Peak7	8.81	11.478	0.132	0.227	1.420
8	Peak8	9.30	37.914	0.435	0.131	6.973
9	Peak9	9.64	22.774	0.261	0.126	4.767
10	Peak10	9.88	16.371	0.188	0.135	3.247
11	Peak11	10.20	89.828	1.031	0.133	17.647
12	Peak12	10.33	160.495	1.841	0.126	32.108
13	Peak13	27.13	39.783	0.456	0.232	4.223

Sampling Rate	120.0017
Injection Volume	5 uL
Detector	UV (231 nm)

Mobile Phase A	Acetonitrile
Mobile Phase B	10mM KH2PO4 pH3HClO4
Gradient	99-54B (30 min)
Temperature	25°C

Column Name	Xbridge C18
Length	15 cm
Diameter	0.46 cm
Particle Size	5 um
Column Flow Rate	2 ml/min

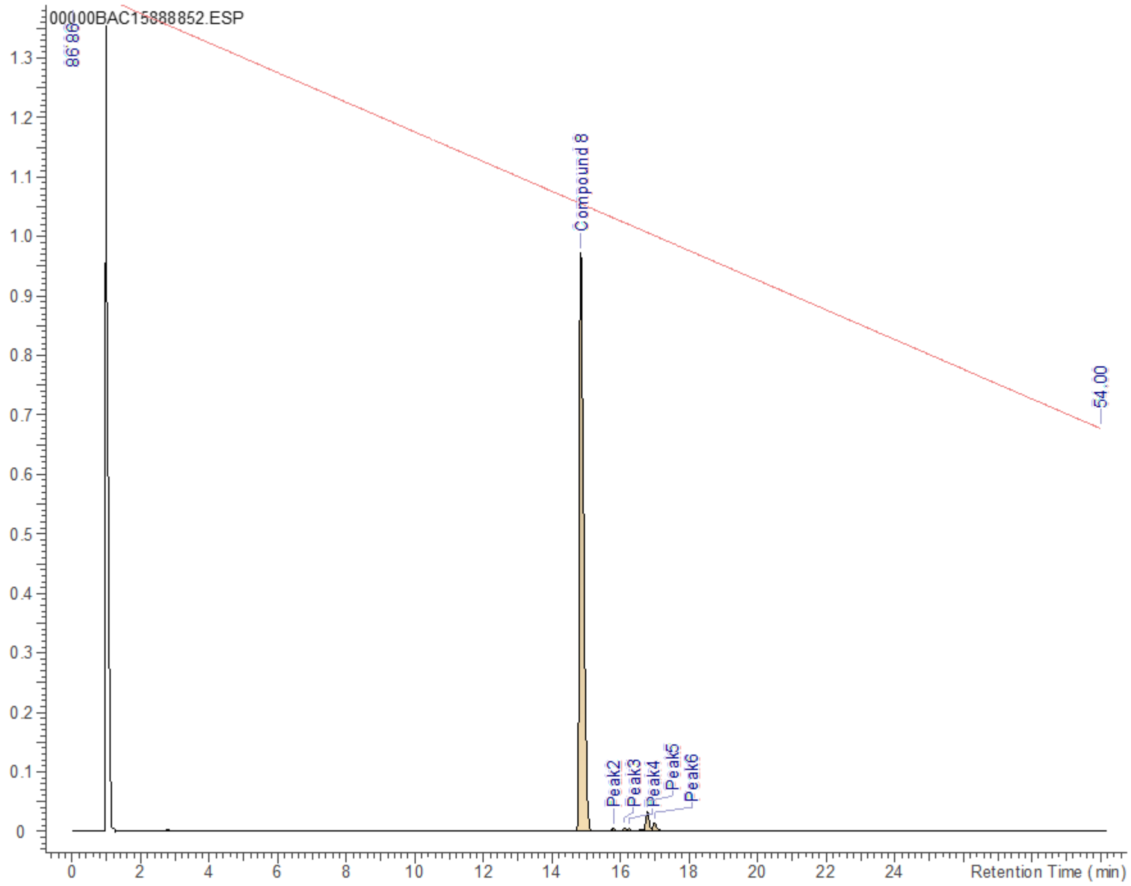

No.	Name	tR	Peak Area (Y units*ms)	Area Percent	Width	Height
1	Compound 8	14.84	8553.905	95.652	0.238	971.057
2	Peak2	15.79	25.038	0.280	0.190	3.306
3	Peak3	16.11	27.699	0.310	0.069	4.697
4	Peak4	16.24	25.948	0.290	0.109	3.686
5	Peak5	16.78	210.063	2.349	0.166	32.439
6	Peak6	16.99	100.119	1.120	0.176	13.614

Injection Volume	5 uL

Detector	UV (252 nm)

Mobile Phase A	ACN+0.01%TFA
Mobile Phase B	water+0.01%TFA
Gradient	99-54B (30 min)
Temperature	25°C

Column Name	Xbridge C18
Length	15 cm
Diameter	0.46 cm
Particle Size	5 um
Column Flow Rate	2 ml/min

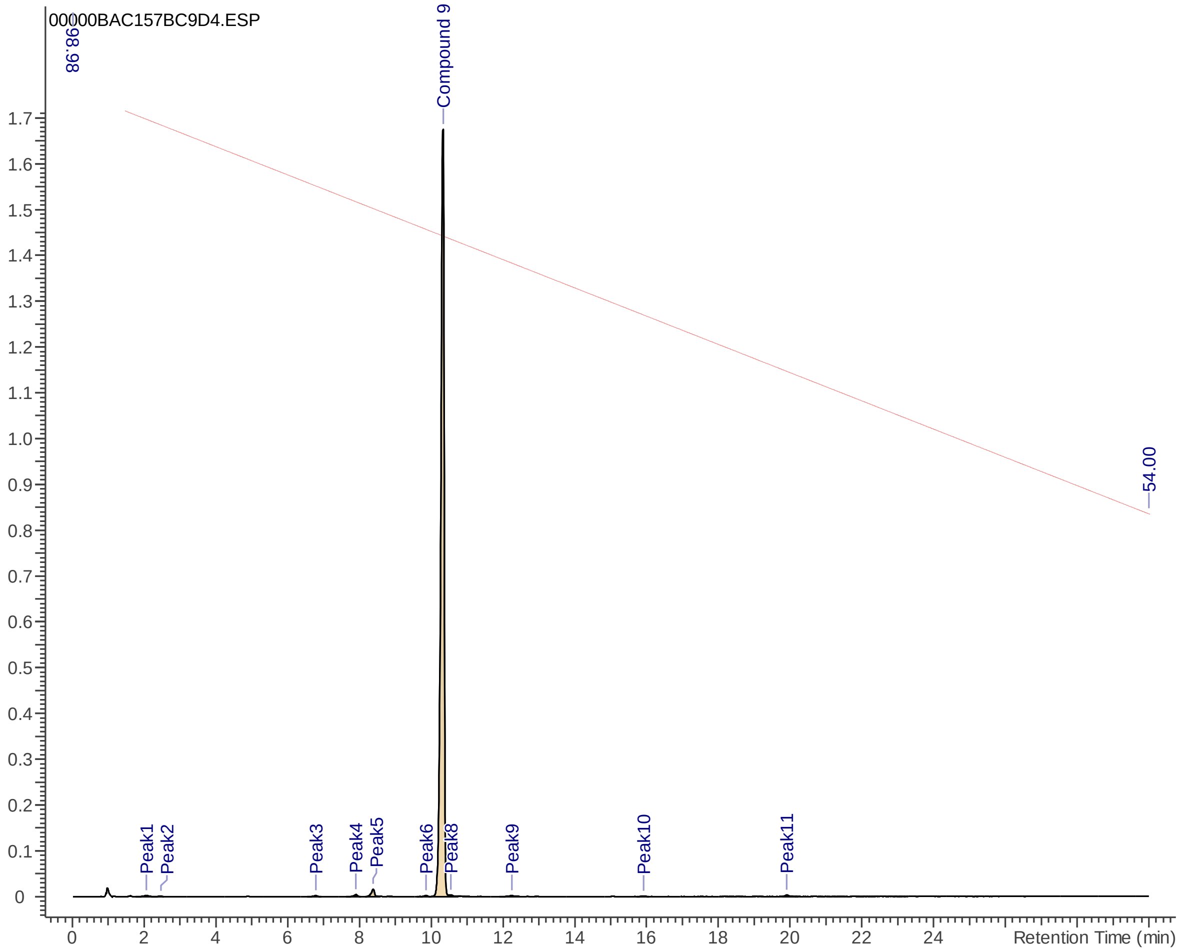

No.	Name	tR	Peak Area (Y units*ms)	Area Percent	Width	Height
1	Peak1	2.05	20.771	0.190	0.274	1.867
2	Peak2	2.47	8.073	0.074	0.165	1.303
3	Peak3	6.79	13.309	0.122	0.141	2.219
4	Peak4	7.90	27.044	0.247	0.152	4.270
5	Peak5	8.38	98.225	0.897	0.150	16.143
6	Peak6	9.85	14.090	0.129	0.135	2.465
7	Compound 9	10.33	10696.009	97.648	0.167	1675.245
8	Peak8	10.55	24.288	0.222	0.096	2.931
9	Peak9	12.25	19.816	0.181	0.208	1.980
10	Peak10	15.91	8.071	0.074	0.155	1.214
11	Peak11	19.91	23.964	0.219	0.189	3.117

Sampling Rate	120.0017
Injection Volume	5 uL
Detector	UV (231 nm)

Mobile Phase A	Acetonitrile
Mobile Phase B	10mM KH2PO4 pH3HClO4
Gradient	99-39B (30 min)
Temperature	25°C

Column Name	Xbridge C18
Length	15 cm
Diameter	0.46 cm
Particle Size	5 um
Column Flow Rate	2 ml/min

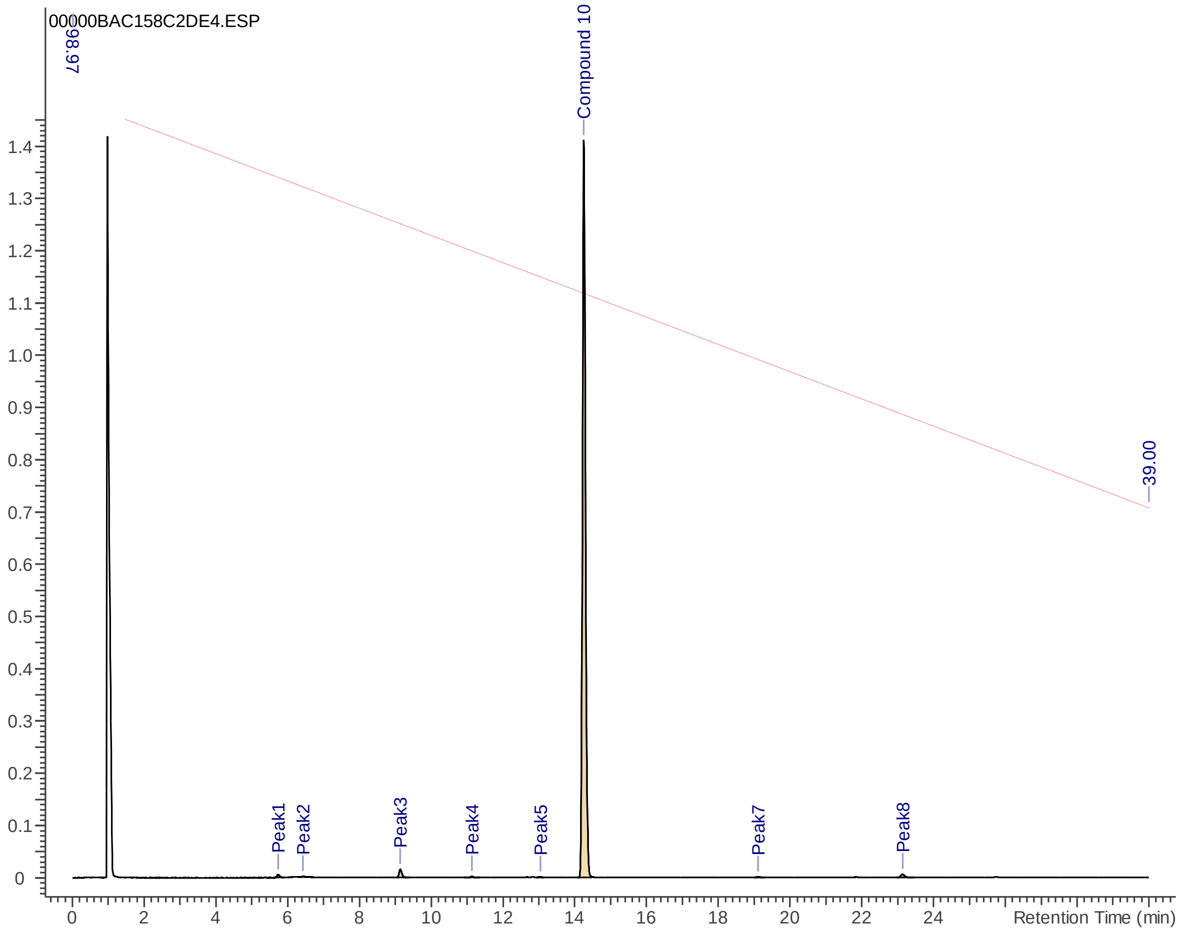

No.	Name	tR	Peak Area (Y units*ms)	Area Percent	Width	Height
1	Peak1	5.73	30.350	0.372	0.148	5.321
2	Peak2	6.43	10.838	0.133	0.152	1.519
3	Peak3	9.14	87.484	1.072	0.141	15.903
4	Peak4	11.14	14.331	0.176	0.136	2.515
5	Peak5	13.04	6.350	0.078	0.164	1.031
6	Compound 10	14.25	7958.520	97.497	0.145	1410.702
7	Peak7	19.11	9.705	0.119	0.215	1.118
8	Peak8	23.14	45.271	0.555	0.177	6.291

Injection Volume	1 uL
Detector	UV (210 nm)
Mobile Phase A	ACN
Mobile Phase B	water+0.01%NH_4CO_3
Gradient	95B (8min)
Temperature	22°C
Column Name	Kinetex EVO C18
Length	5 cm
Diameter	0.46 cm
Particle Size	2.6 um
Column Flow Rate	1.5 ml/min

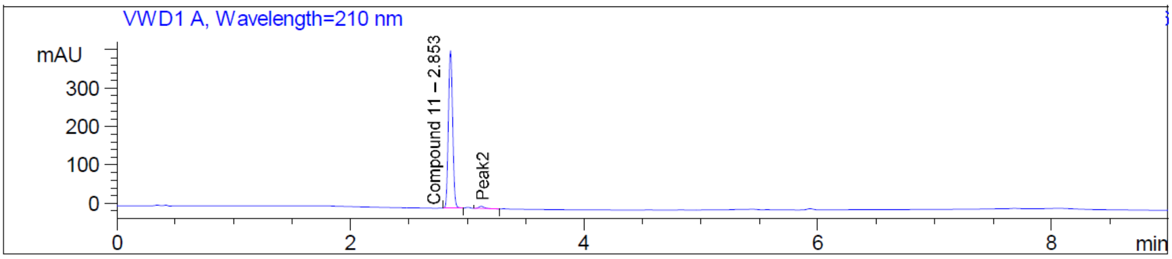

# Meas.	Ret	Peak	Width	Area	Response
Compd 11	2.853	VV	0.040	1.047e3	97.488
Peak2	3.116	VV	0.059	26.985	2.512

Sampling Rate	120.002

Injection Volume	5 uL

Detector	UV (246 nm)

Mobile Phase A	ACN+0.01%TFA
Mobile Phase B	water+0.01%TFA
Gradient	85-40B (30 min)
Temperature	25°C

Column Name	Xbridge C18
Length	15 cm
Diameter	0.46 cm
Particle Size	5 um
Column Flow Rate	2 ml/min

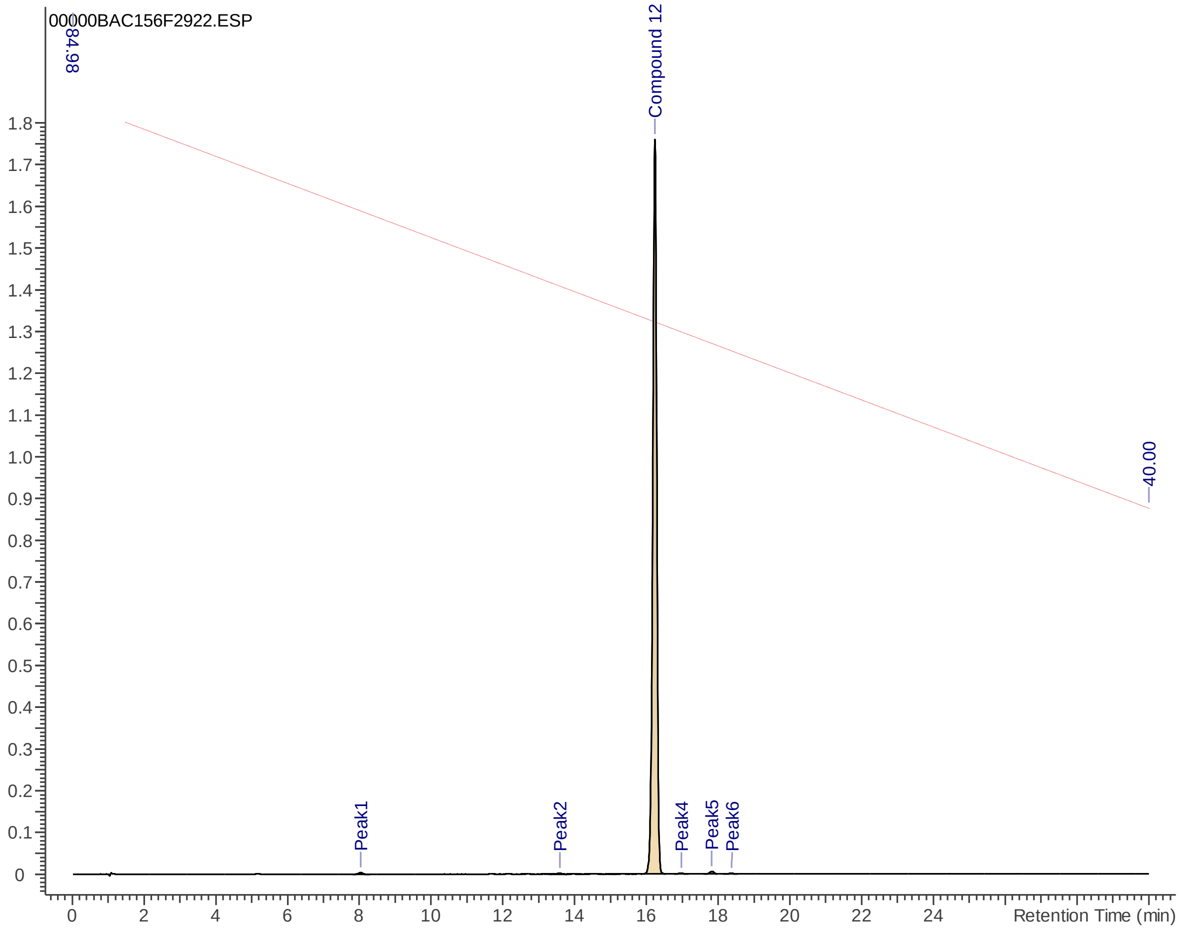

No.	Name	tR	Peak Area (Y units*ms)	Area Percent	Width	Height
1	Peak1	8.04	27.763	0.204	0.180	3.852
2	Peak2	13.58	22.097	0.162	0.208	2.472
3	Compound 12	16.24	13464.981	99.001	0.199	1759.242
4	Peak4	16.97	18.712	0.138	0.244	1.868
5	Peak5	17.82	49.314	0.363	0.208	6.119
6	Peak6	18.37	18.049	0.133	0.208	2.083

Sampling Rate	120.002
Injection Volume	5 uL
Detector	UV (222 nm)

Mobile Phase A	Acetonitrile
Mobile Phase B	10mM KH2PO4 pH3HClO4
Gradient	99-69B (30 min)
Temperature	25°C

Column Name	Xbridge C18
Length	15 cm
Diameter	0.46 cm
Particle Size	5 um
Column Flow Rate	2 ml/min

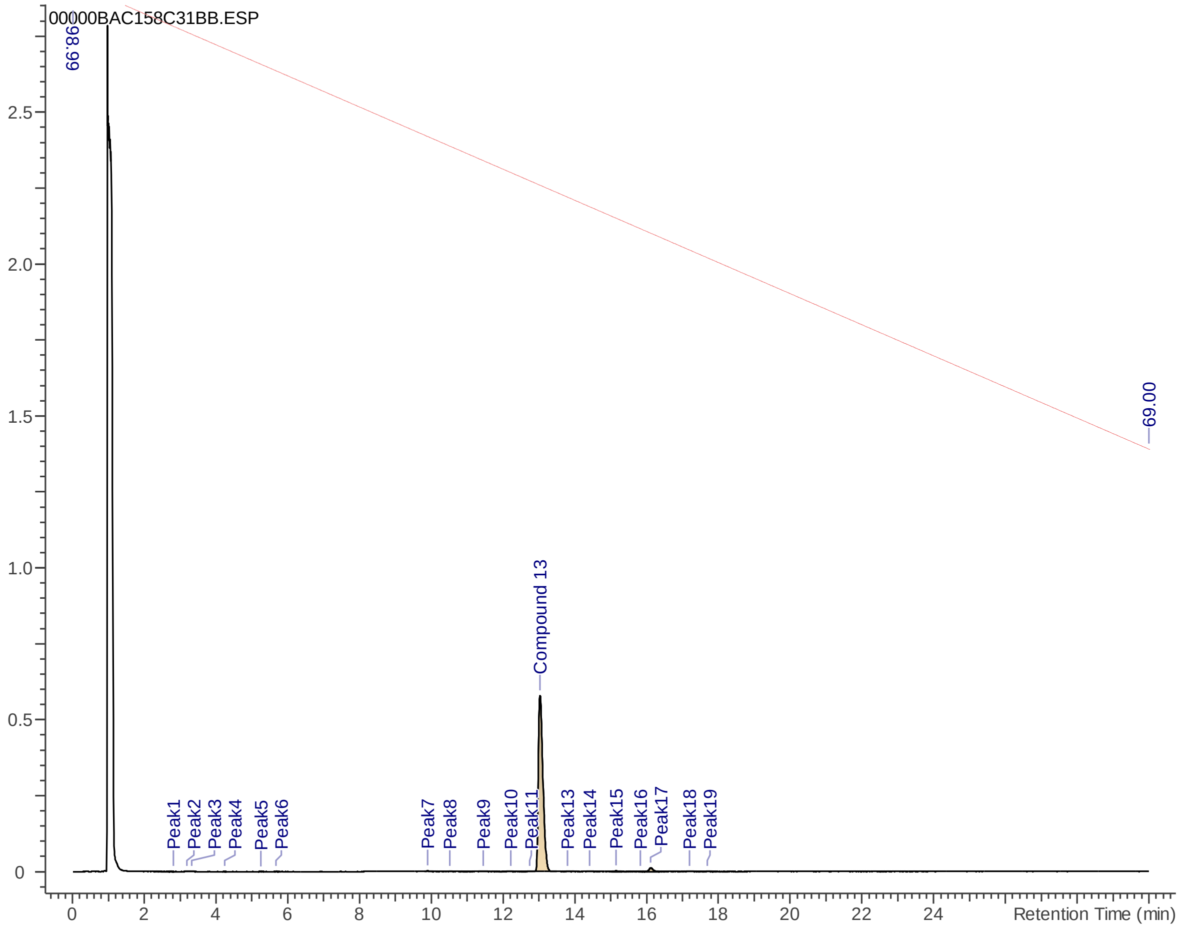

No.	Name	tR	Peak Area (Y units*ms)	Area Percent	Width	Height
1	Peak1	2.82	1.563	0.029	0.150	347256.313
2	Peak2	3.18	3.338	0.063	0.100	790386.250
3	Peak3	3.33	12.593	0.237	0.233	1393638.000
4	Peak4	4.24	1.047	0.020	0.100	321880.438
5	Peak5	5.24	2.517	0.047	0.150	437450.469
6	Peak6	5.68	1.751	0.033	0.125	340323.375
7	Peak7	9.89	12.124	0.228	0.250	1911032.500
8	Peak8	10.52	1.420	0.027	0.108	313021.781
9	Peak9	11.45	8.413	0.159	0.283	1184674.250
10	Peak10	12.21	2.475	0.047	0.192	395336.813
11	Peak11	12.75	3.137	0.059	0.150	471904.531
12	Compound 13	13.03	5134.103	96.739	0.683	578861248.000
13	Peak13	13.79	2.667	0.050	0.233	367421.063

Sampling Rate	120.002
Injection Volume	5 uL
Detector	UV (217 nm)

Mobile Phase A	Acetonitrile
Mobile Phase B	10mM KH2PO4 pH3HClO4
Gradient	95-50B (30 min)
Temperature	25°C

Column Name	Xbridge C18
Length	15 cm
Diameter	0.46 cm
Particle Size	5 um
Column Flow Rate	2 ml/min

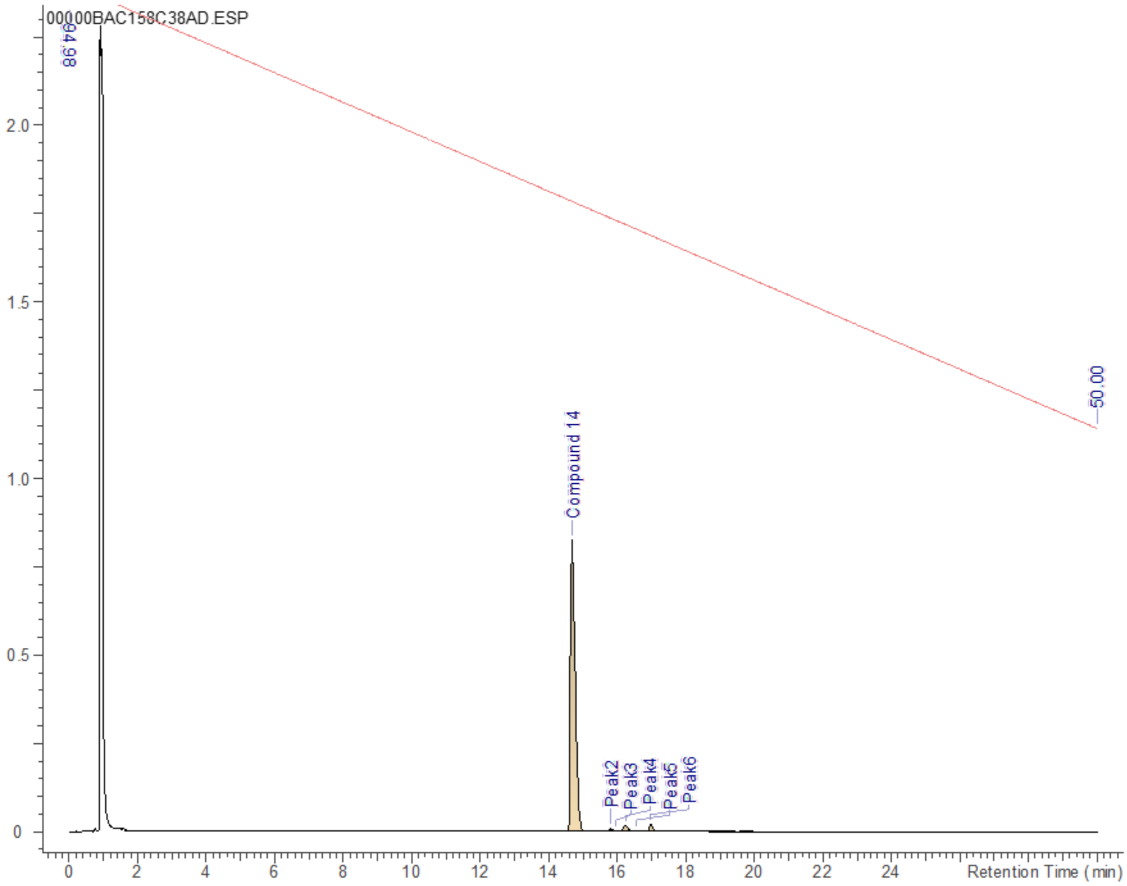

No.	Name	tR	Peak Area (Y units*ms)	Area Percent	Width	Height
1	Compound 14	14.68	8035.843	95.133	0.264	824.394
2	Peak2	15.82	58.945	0.698	0.199	7.159
3	Peak3	15.95	17.589	0.208	0.126	2.826
4	Peak4	16.24	163.902	1.940	0.256	16.797
5	Peak5	16.55	20.933	0.248	0.197	2.655
6	Peak6	16.98	149.701	1.772	0.191	19.263

Injection Volume	5 uL

Detector	UV (222 nm)

Mobile Phase A	ACN+0.01%TFA
Mobile Phase B	water+0.01%TFA
Gradient	99-54B (30 min)
Temperature	25°C

Column Name	Xbridge C18
Length	15 cm
Diameter	0.46 cm
Particle Size	5 um
Column Flow Rate	2 ml/min

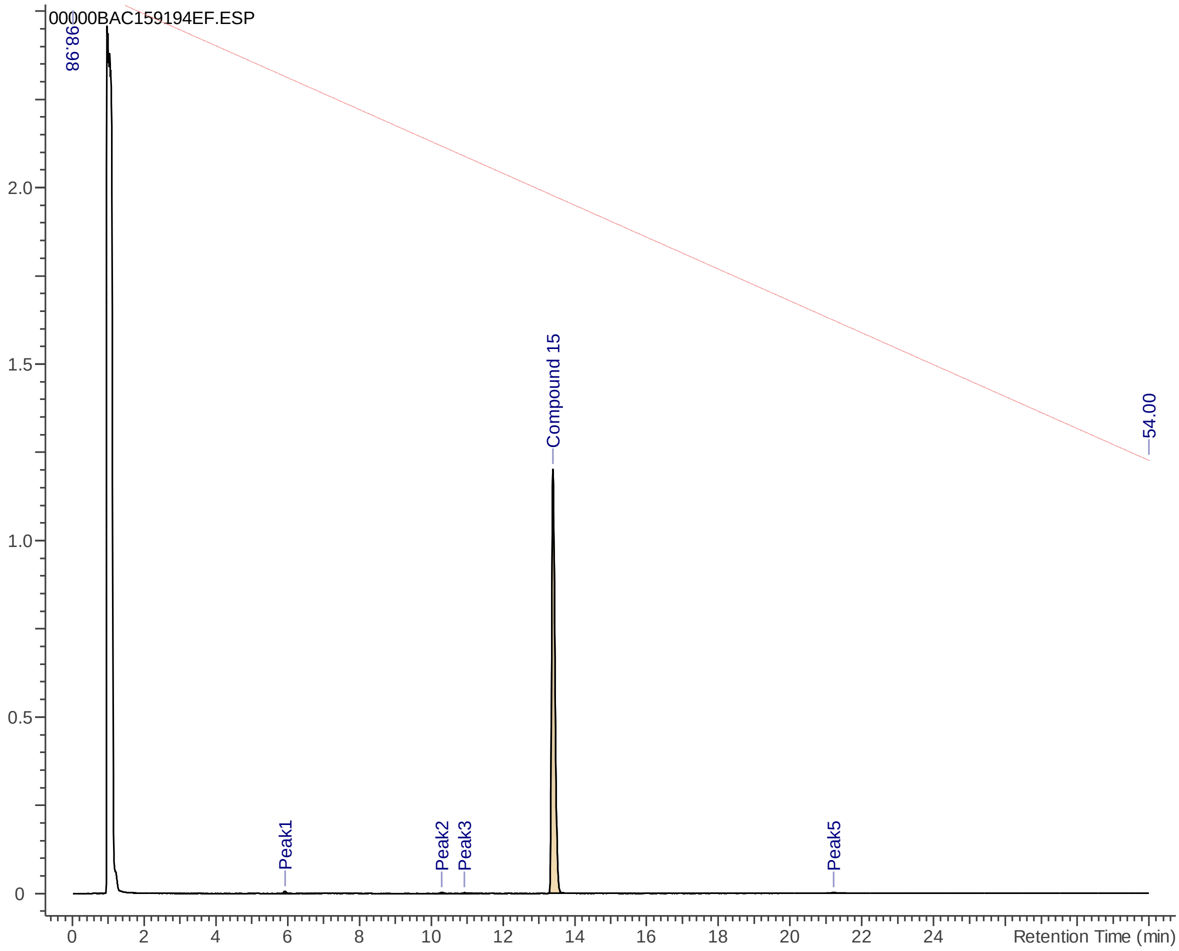

No.	Name	tR	Peak Area (Y units*ms)	Area Percent	Width	Height
1	Peak1	5.92	31.841	0.410	0.129	6.158
2	Peak2	10.30	16.881	0.217	0.134	2.886
3	Peak3	10.93	12.970	0.167	0.131	2.004
4	Compound 15	13.40	7683.434	98.910	0.169	1200.746
5	Peak5	21.21	22.943	0.295	0.187	2.786

Sampling Rate	120.002

Injection Volume	5 uL

Detector	UV (235 nm)

Mobile Phase A	ACN+0.01%TFA
Mobile Phase B	water+0.01%TFA
Gradient	90-45B (30 min)
Temperature	25°C

Column Name	Xbridge C18
Length	15 cm
Diameter	0.46 cm
Particle Size	5 um
Column Flow Rate	2 ml/min

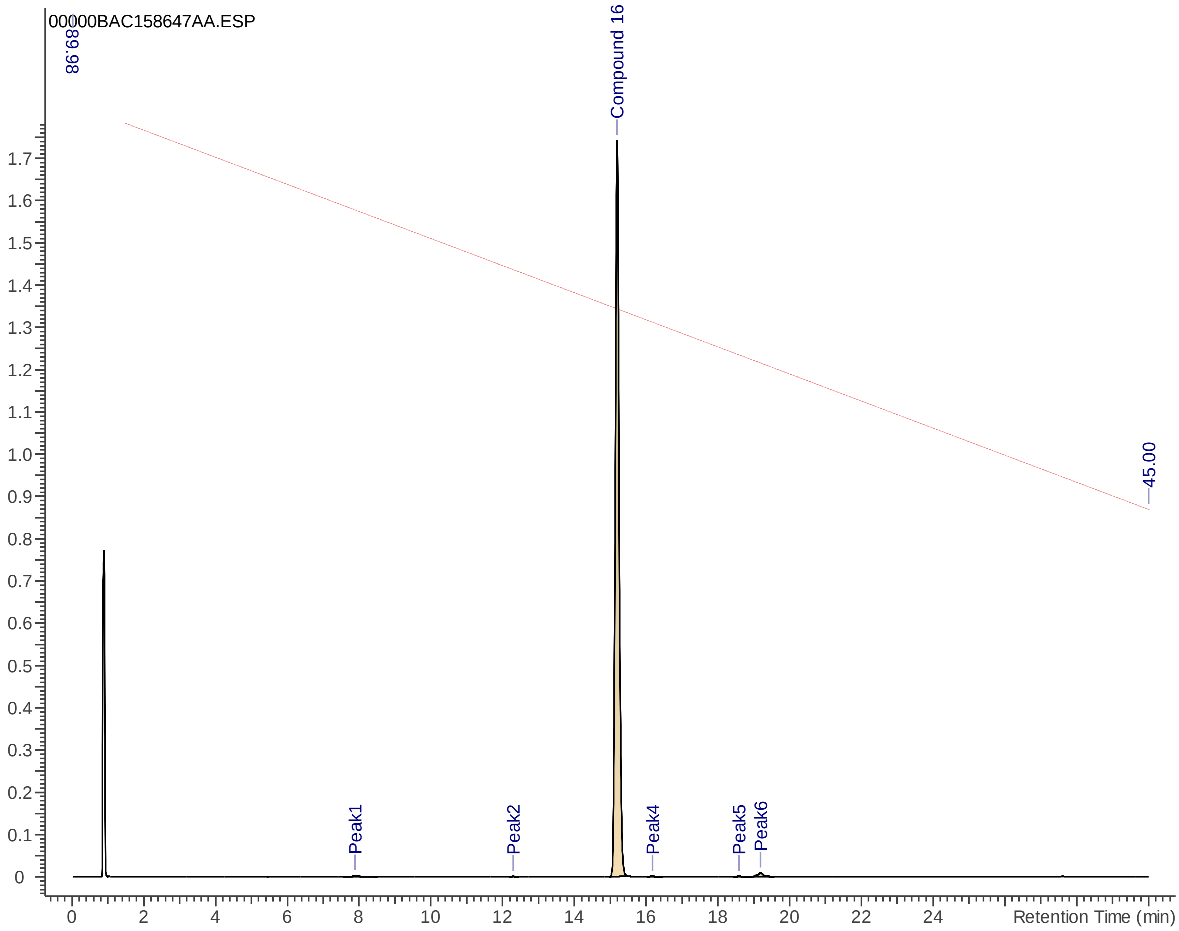

No.	Name	tR	Peak Area (Y units*ms)	Area Percent	Width	Height
1	Peak1	7.88	41.542	0.324	0.310	3.322
2	Peak2	12.29	7.573	0.059	0.195	1.062
3	Compound 16	15.19	12659.291	98.652	0.187	1741.617
4	Peak4	16.17	12.063	0.094	0.198	1.237
5	Peak5	18.59	11.435	0.089	0.205	1.245
6	Peak6	19.19	100.305	0.782	0.240	9.075

Injection Volume	5 uL
Detector	UV (234 nm)
Mobile Phase A	ACN+0.01%TFA
Mobile Phase B	water+0.01%TFA
Gradient	99-69B (30 min)
Temperature	22°C
Column Name	Xbridge C18
Length	15 cm
Diameter	0.46 cm
Particle Size	5 um
Column Flow Rate	2ml/min

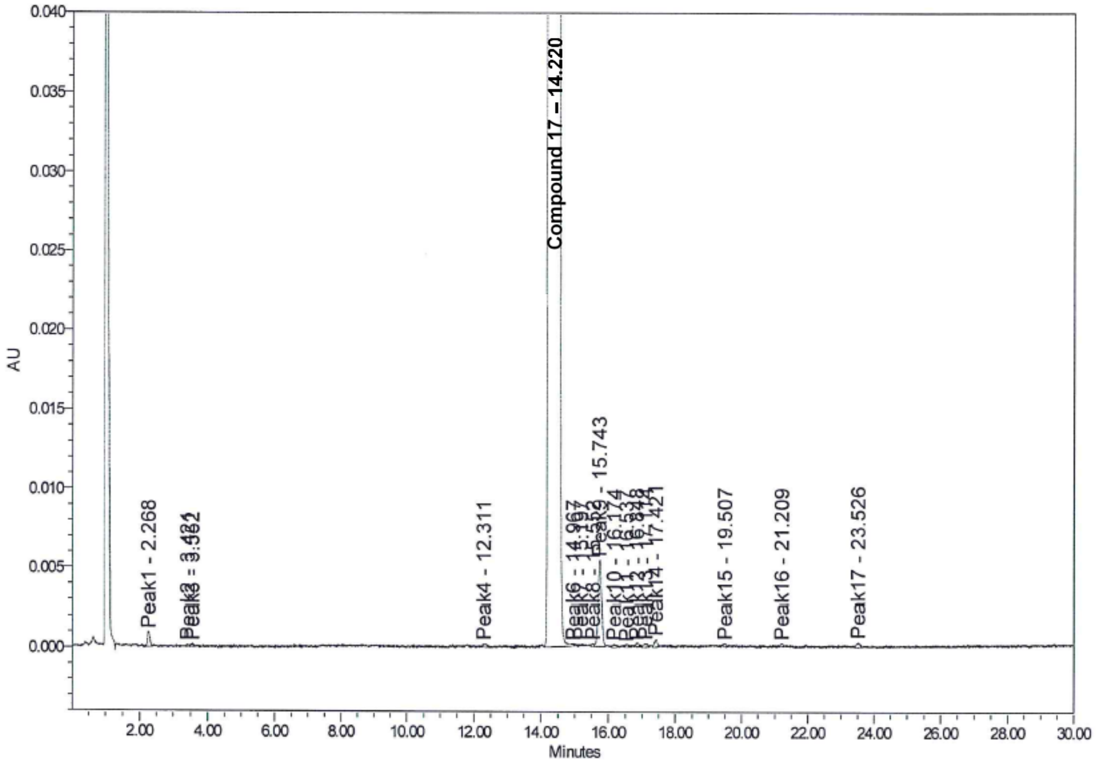

Peak Results

	Peak Name	RT	Area (µV*sec)	% Area	RT Ratio
1	Peak1	2.268	4949	0.077	0.159
2	Peak2	3.421	623	0.010	0.241
3	Peak3	3.552	1134	0.018	0.250
4	Peak4	12.311	1912	0.030	0.866
5	Compd17	14.220	6401174	99.041	
6	Peak6	14.967	542	0.008	1.053
7	Peak7	15.197	669	0.010	1.069
8	Peak8	15.552	792	0.012	1.094
9	Peak9	15.743	38570	0.597	1.107
10	Peak10	16.174	1154	0.018	1.137
11	Peak11	16.537	1073	0.017	1.163
12	Peak12	16.848	1718	0.027	1.185
13	Peak13	17.114	1805	0.028	1.204
14	Peak14	17.421	2747	0.043	1.225
15	Peak15	19.507	973	0.015	1.372
16	Peak16	21.209	1021	0.016	1.492
17	Peak17	23.526	2302	0.036	1.654

Sampling Rate	120.0017
Injection Volume	5 uL
Detector	UV (246 nm)

Mobile Phase A	ACN+0.01%TFA
Mobile Phase B	water+0.01%TFA
Gradient	75-30B (30 min)
Temperature	25°C

Column Name	Xbridge C18
Length	15 cm
Diameter	0.46 cm
Particle Size	5 um
Column Flow Rate	2 ml/min

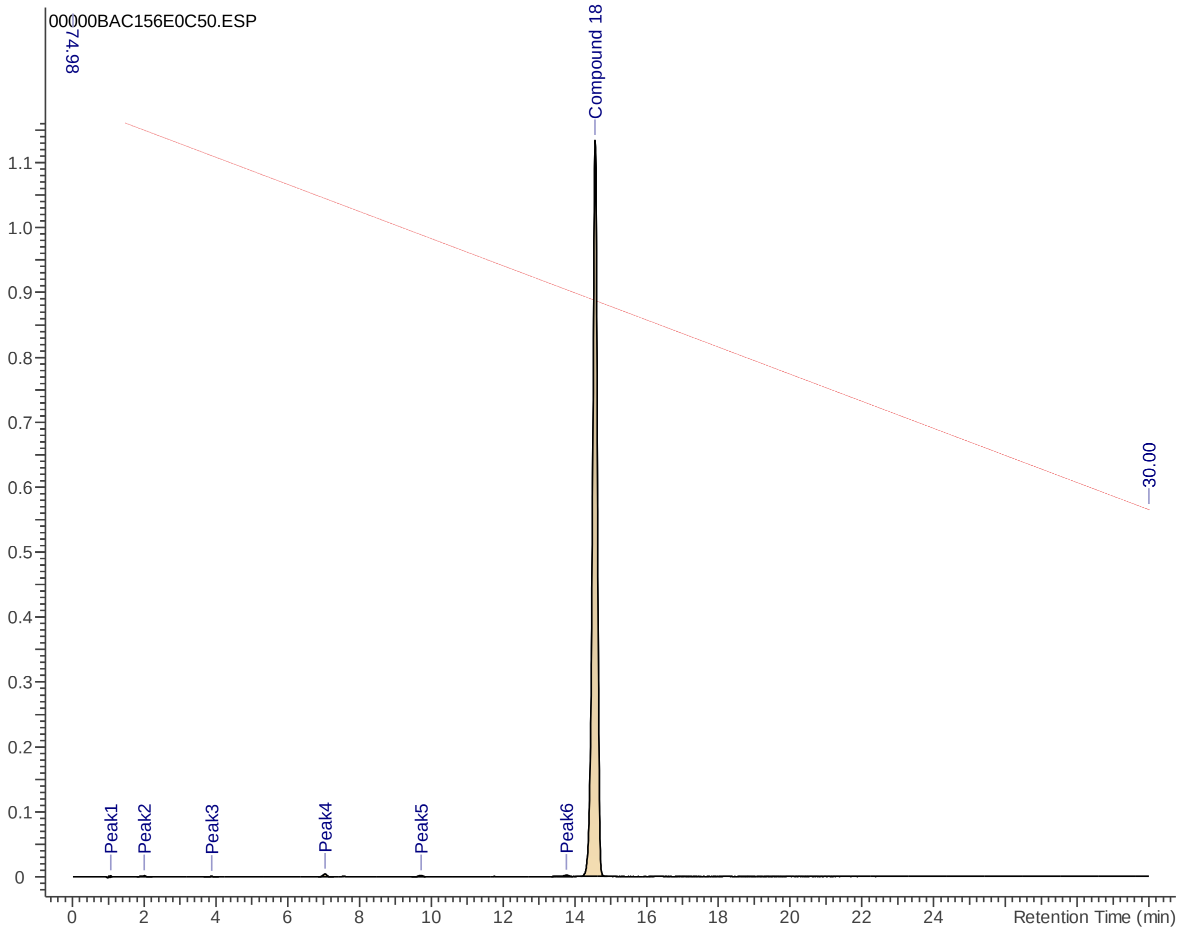

No.	Name	tR	Peak Area (Y units*ms)	Area Percent	Width	Height
1	Peak1	1.07	9.980	0.092	0.152	1.896
2	Peak2	2.00	13.836	0.128	0.157	1.654
3	Peak3	3.88	6.682	0.062	0.139	1.014
4	Peak4	7.04	33.129	0.307	0.171	4.682
5	Peak5	9.71	14.613	0.135	0.196	1.879
6	Peak6	13.77	26.458	0.245	0.262	2.073
7	Compound 18	14.57	10693.979	99.030	0.244	1133.489

¹HNMR and ¹³CNMR spectra of final compounds

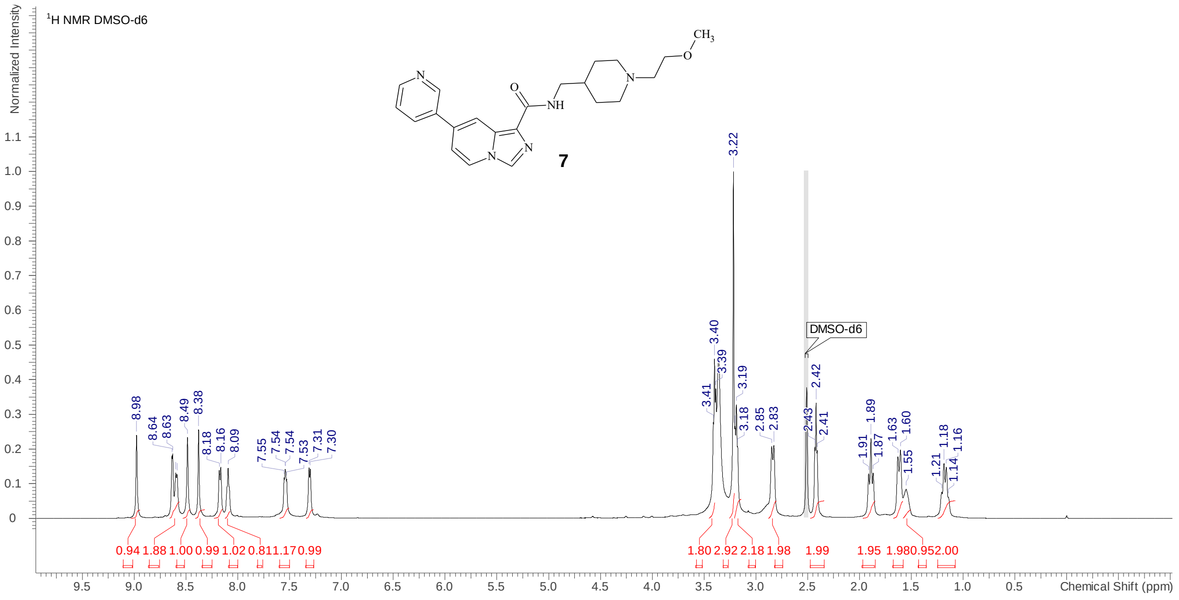

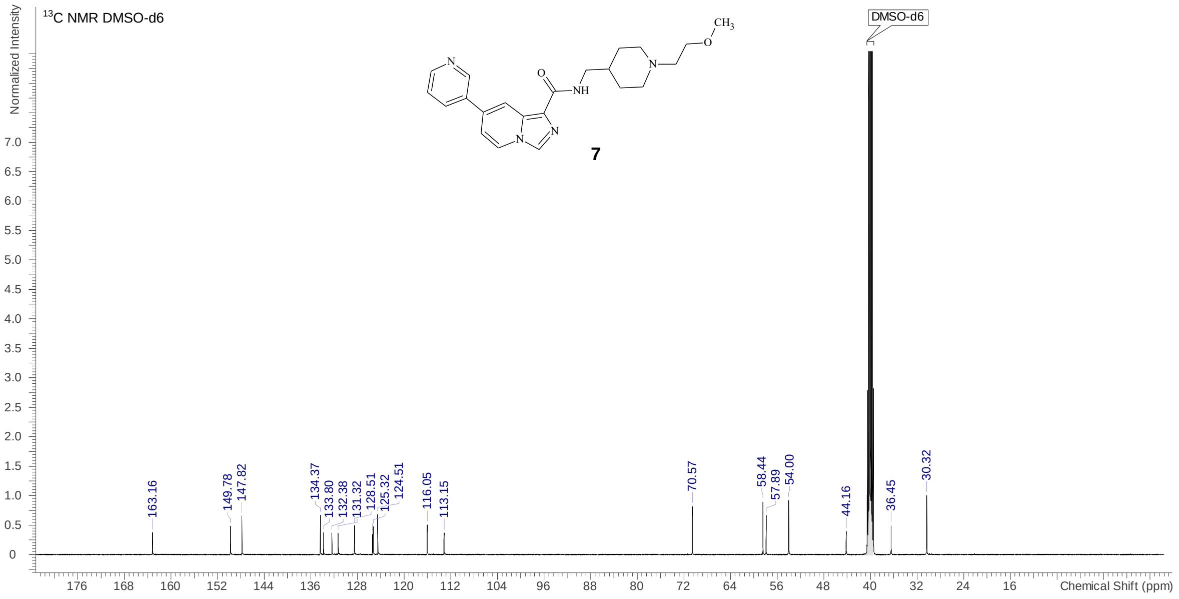

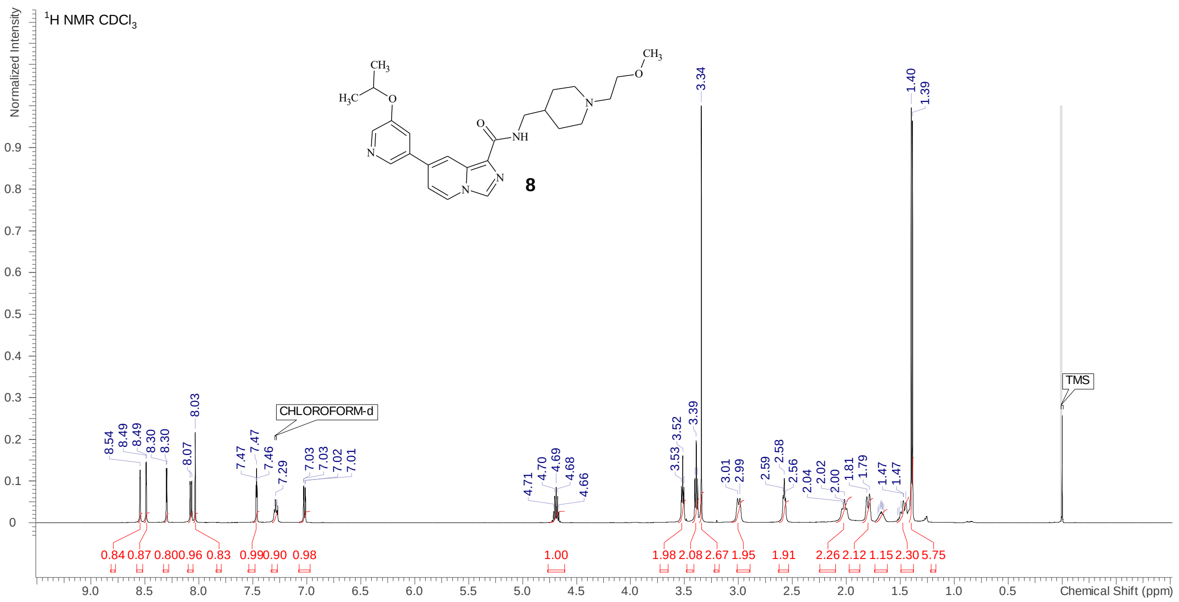

^{1}H NMR CDCl$_3$
Normalized Intensity
8
CHLOROFORM-d
TMS
Chemical Shift (ppm)

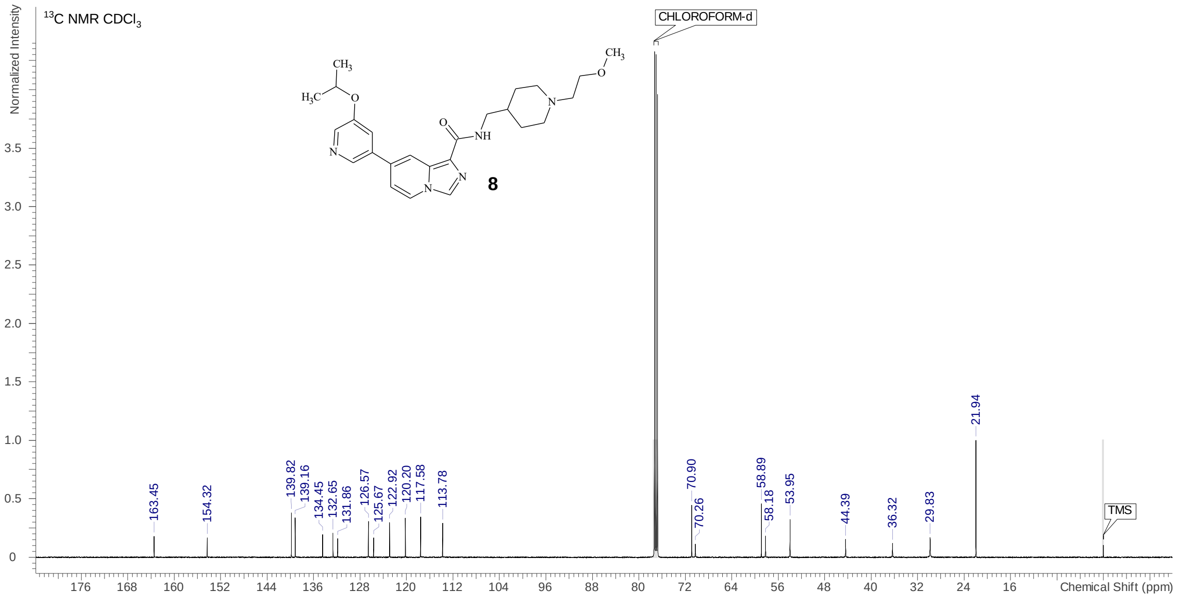

^{13}C NMR CDCl$_3$
Normalized Intensity
8
CHLOROFORM-d
TMS
Chemical Shift (ppm)

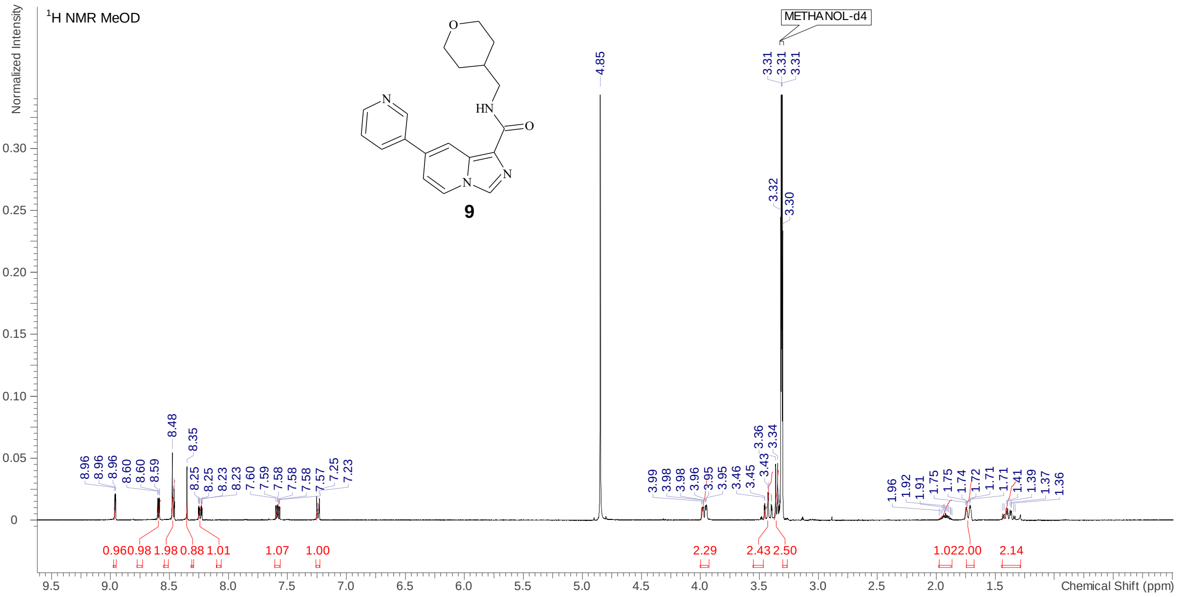
1H NMR MeOD
Normalized Intensity
METHANOL-d4
9
0.96 0.98 1.98 0.88 1.01 1.07 1.00 2.29 2.43 2.50 1.02 2.00 2.14
Chemical Shift (ppm)

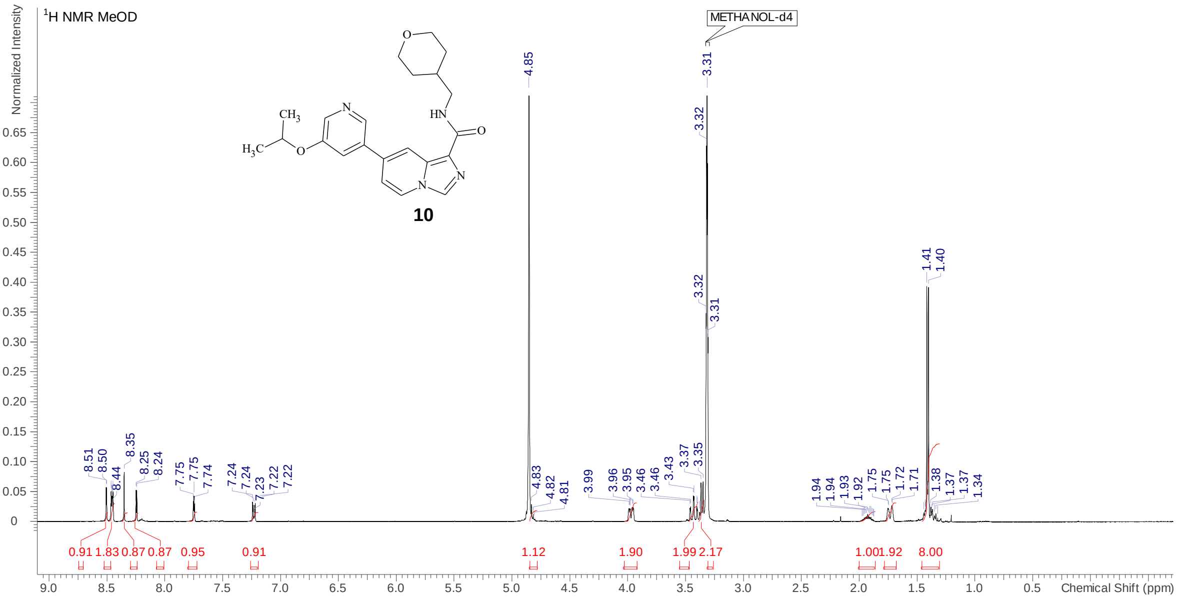
1H NMR MeOD
Normalized Intensity
METHANOL-d4
10
0.91 1.83 0.87 0.87 0.95 0.91 1.12 1.90 1.99 2.17 1.00 1.92 8.00
Chemical Shift (ppm)

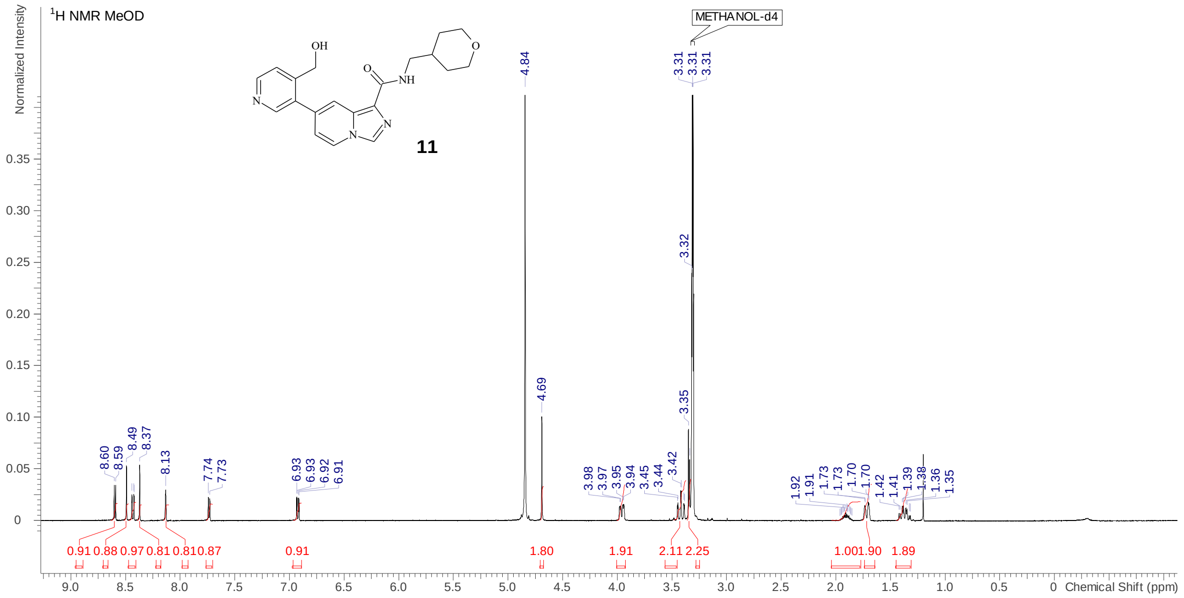

1H NMR MeOD
Normalized Intensity
METHANOL-d4
11
Chemical Shift (ppm)

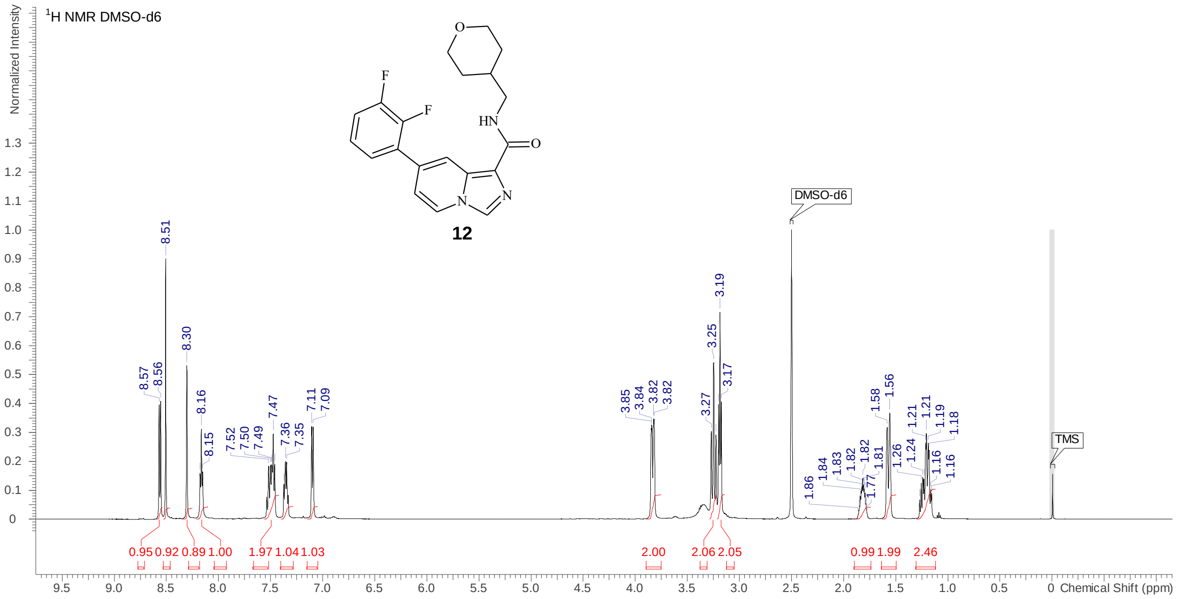

1H NMR DMSO-d6
Normalized Intensity
Chemical Shift (ppm)
DMSO-d6
TMS

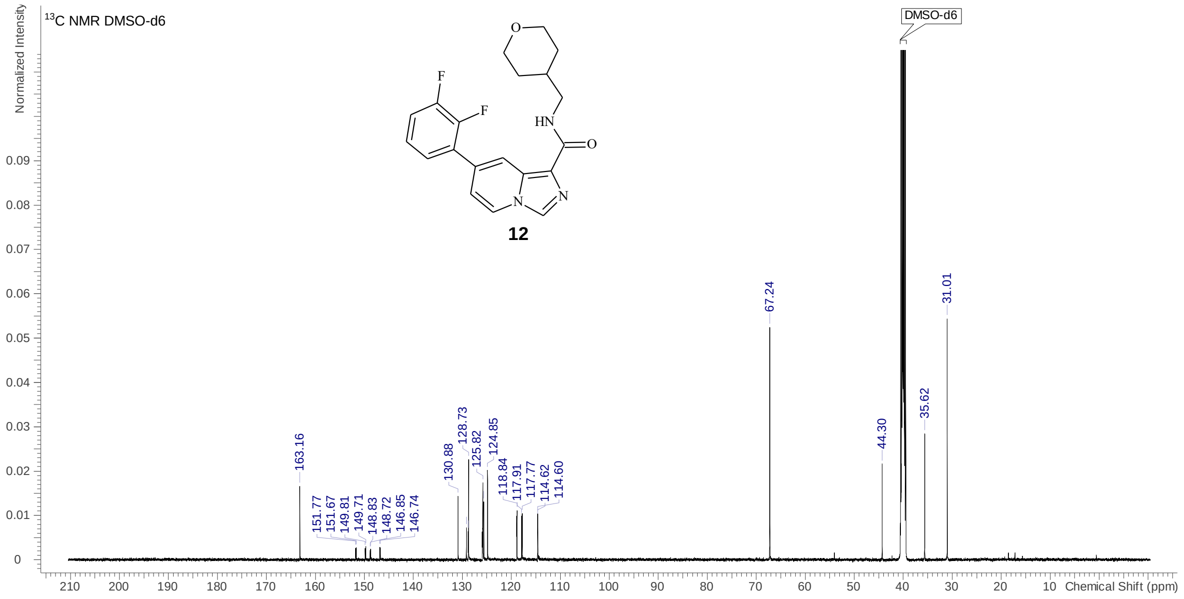

13C NMR DMSO-d6
Normalized Intensity
Chemical Shift (ppm)
DMSO-d6

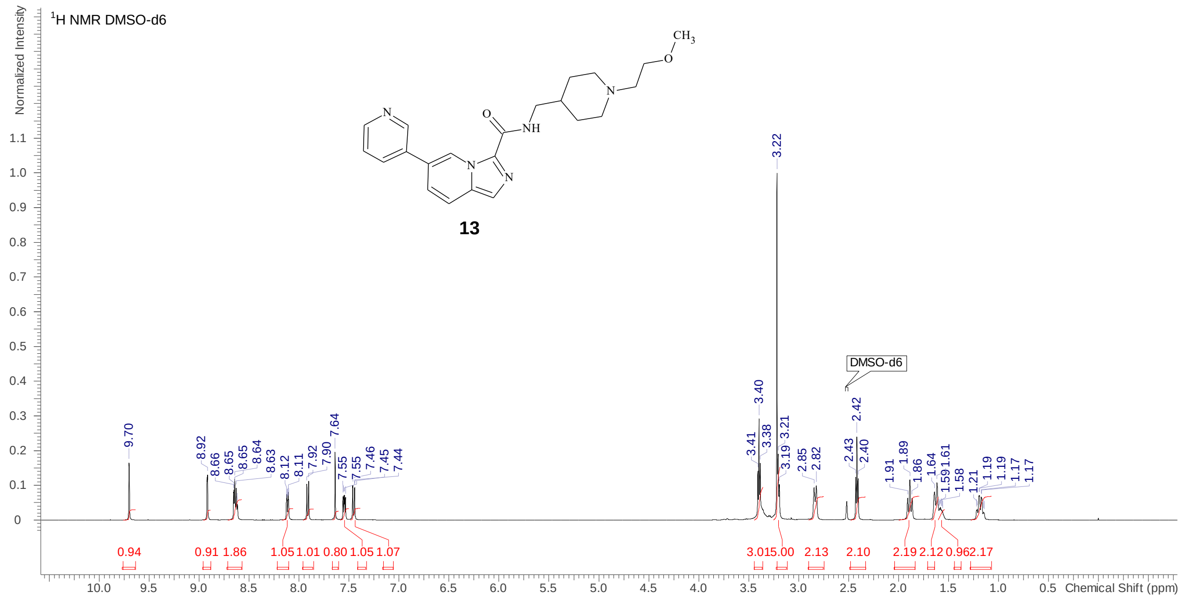

1H NMR DMSO-d6
13
DMSO-d6
9.70
8.92 8.66 8.65 8.65 8.64 8.63 8.12 8.11 7.92 7.90 7.64 7.55 7.55 7.46 7.45 7.44
3.41 3.40 3.38 3.22 3.21 3.19 2.85 2.82 2.43 2.42 2.40 1.91 1.89 1.86 1.64 1.61 1.59 1.58 1.21 1.19 1.19 1.17 1.17
0.94 0.91 1.86 1.05 1.01 0.80 1.05 1.07 3.01 15.00 2.13 2.10 2.19 2.12 0.96 2.17
Normalized Intensity
Chemical Shift (ppm)

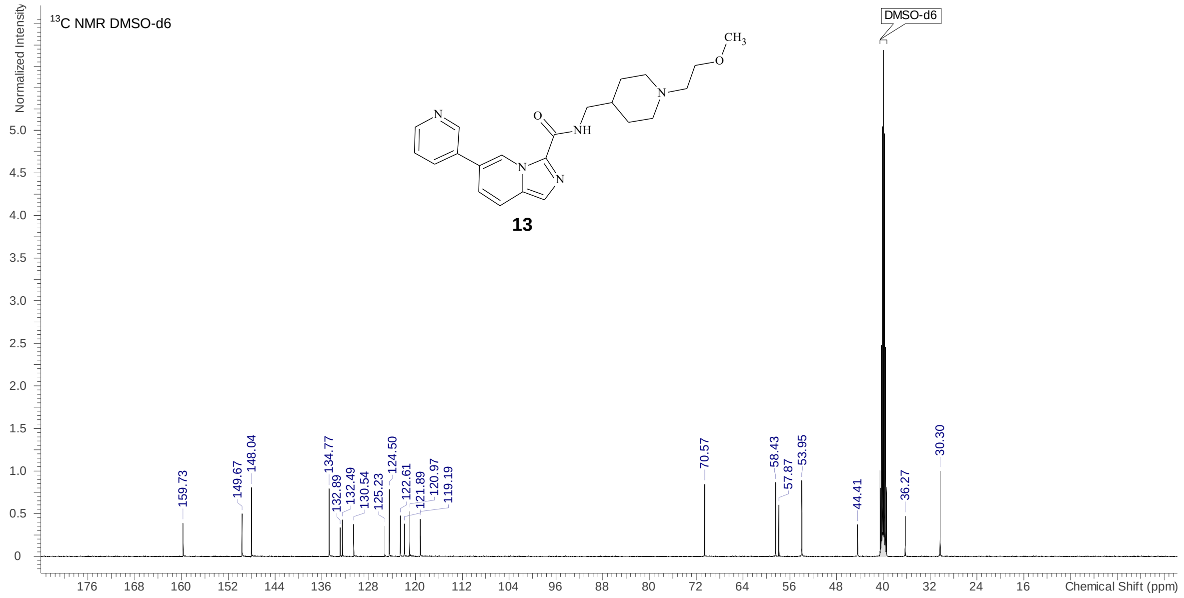

13C NMR DMSO-d6
13
DMSO-d6
159.73 149.67 148.04 134.77 132.89 132.49 130.54 125.23 124.50 122.61 121.89 120.97 119.19
70.57 58.43 57.87 53.95 44.41 36.27 30.30
Normalized Intensity
Chemical Shift (ppm)

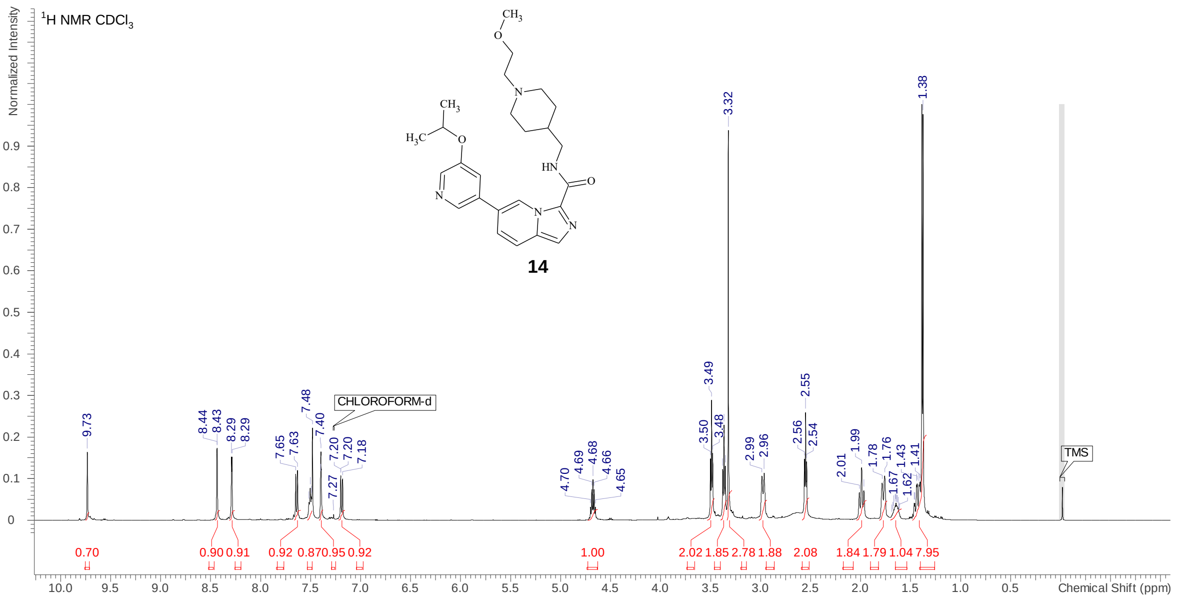
^{1}H NMR $CDCl_3$
Normalized Intensity
CHLOROFORM-d
TMS
14
Chemical Shift (ppm)

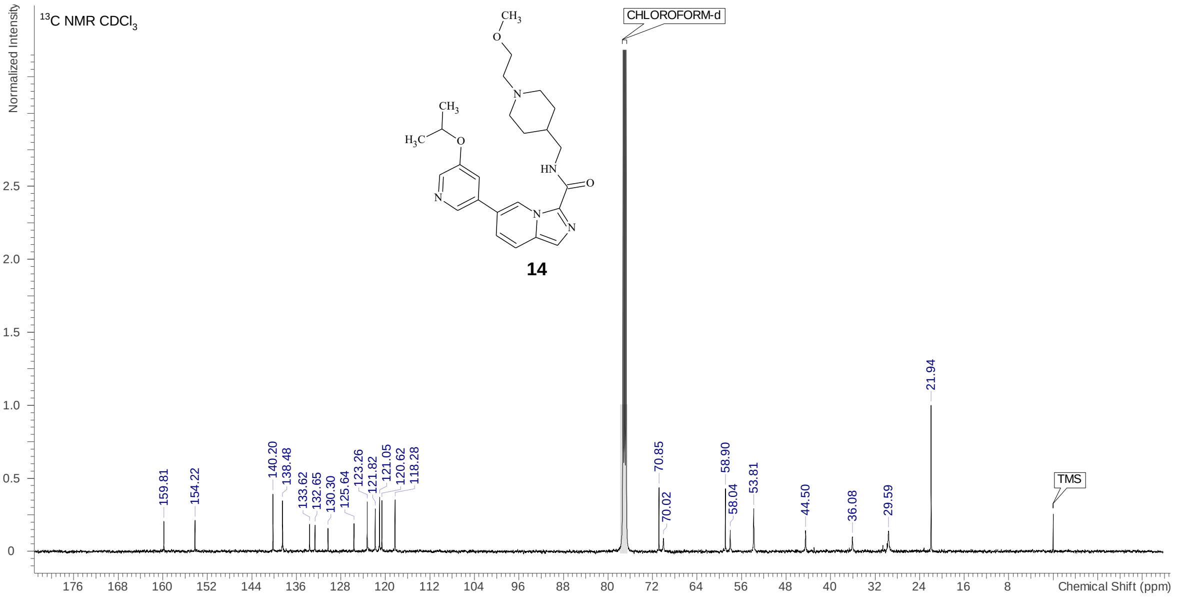
^{13}C NMR $CDCl_3$
Normalized Intensity
CHLOROFORM-d
TMS
14
Chemical Shift (ppm)

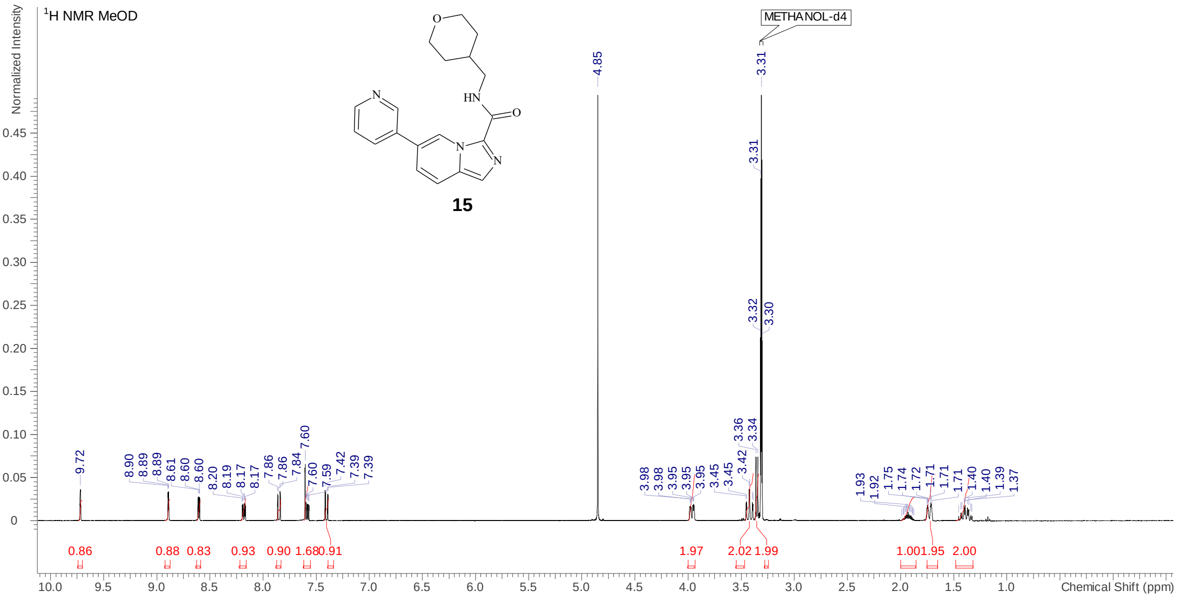

^{1}H NMR MeOD
Normalized Intensity
METHANOL-d4
Chemical Shift (ppm)

15

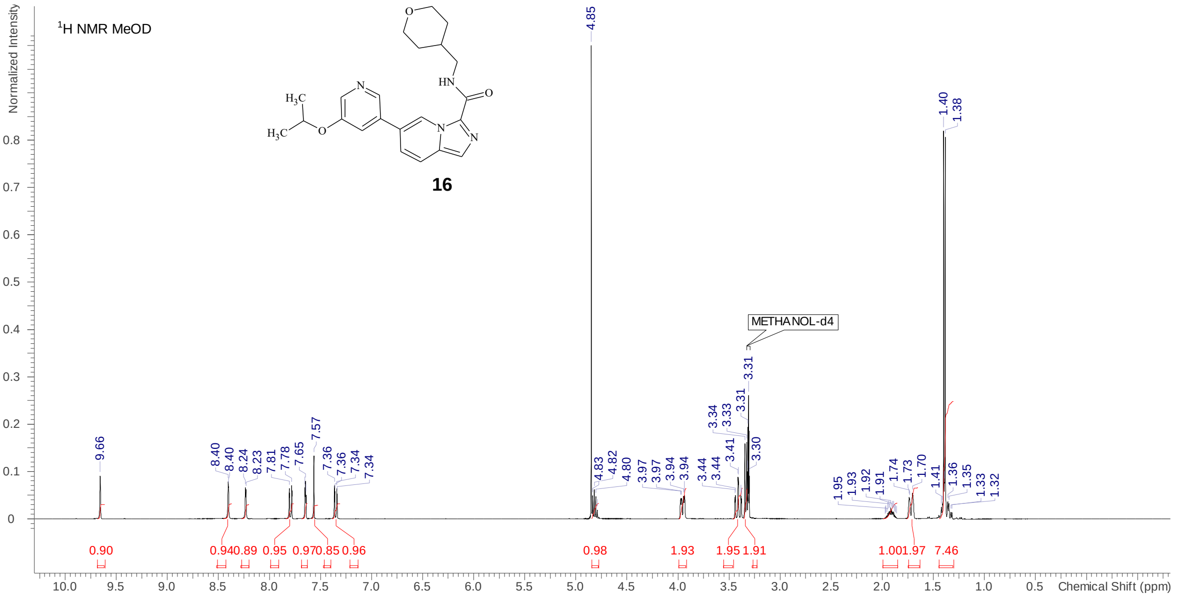

^{1}H NMR MeOD
Normalized Intensity
METHANOL-d4
Chemical Shift (ppm)

16

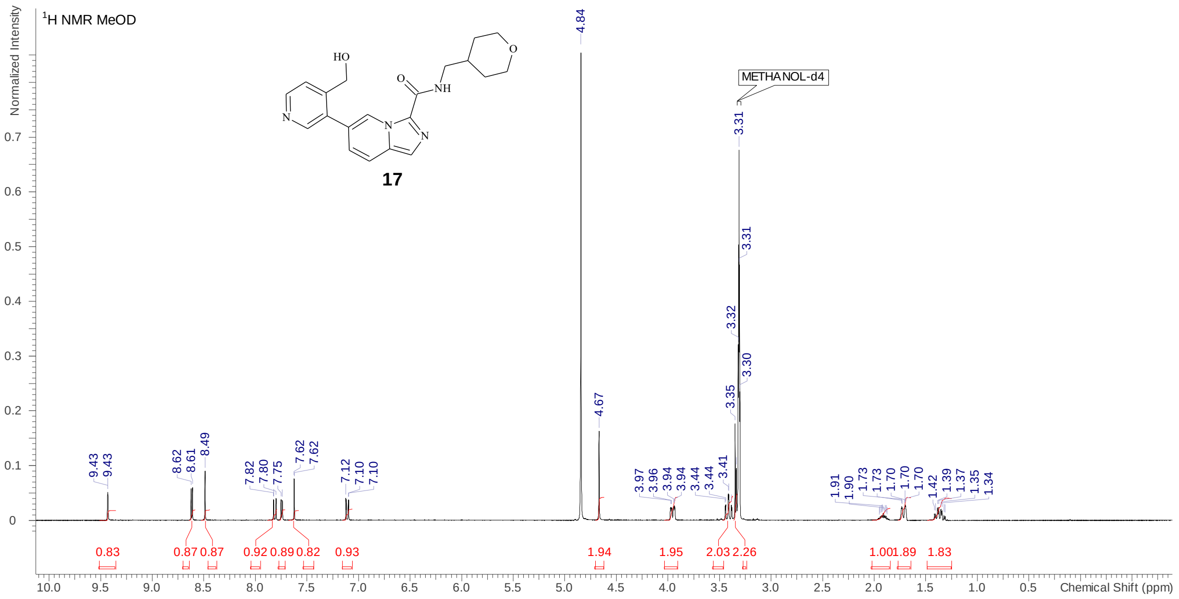

^{1}H NMR MeOD
Normalized Intensity
HO
O
NH
N
N
N
O
17
METHANOL-d4
9.43 9.43
8.62 8.61 8.49
7.82 7.80 7.75
7.62 7.62
7.12 7.10 7.10
4.84
4.67
3.97 3.96 3.94 3.94 3.44 3.44 3.41
3.35 3.32 3.31 3.31 3.30
1.91 1.90 1.73 1.73 1.70 1.70 1.70 1.42 1.39 1.37 1.35 1.34
0.83 0.87 0.87 0.92 0.89 0.82 0.93 1.94 1.95 2.03 2.26 1.00 1.89 1.83
Chemical Shift (ppm)

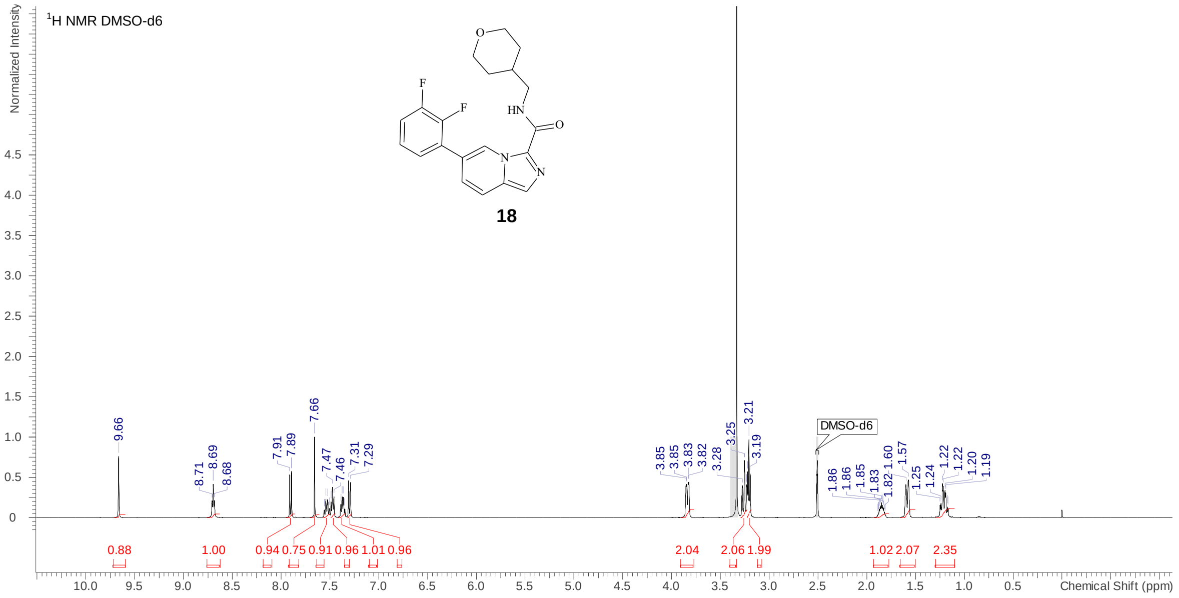

1H NMR DMSO-d6
18
DMSO-d6
Normalized Intensity
Chemical Shift (ppm)

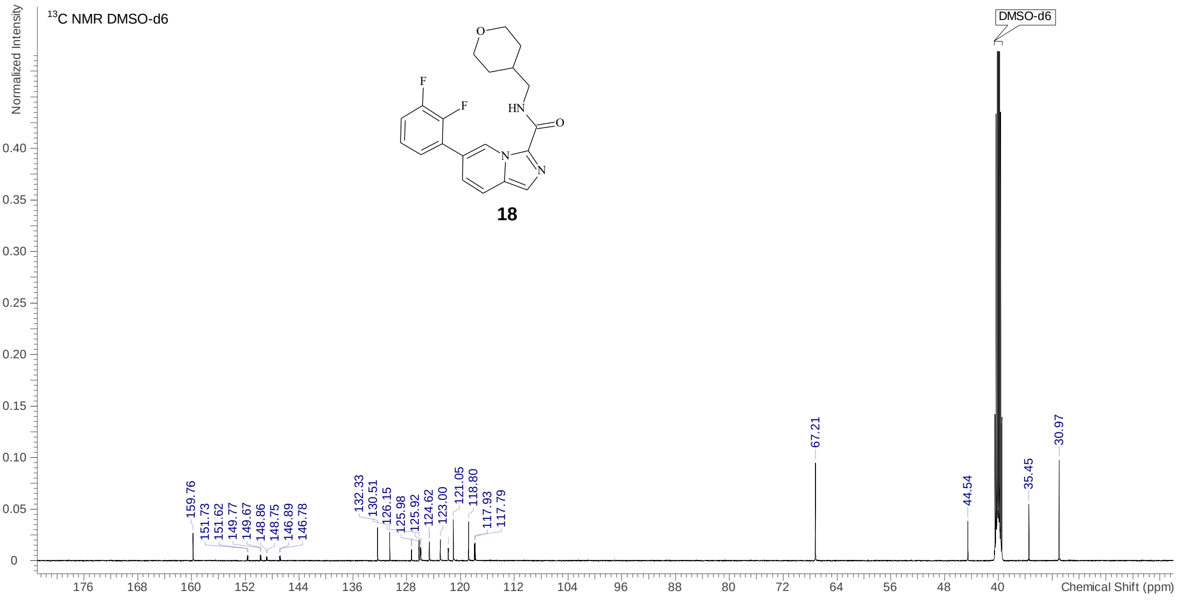

13C NMR DMSO-d6
18
DMSO-d6
Normalized Intensity
Chemical Shift (ppm)

2D NMR spectra of compound 2

^{1}H-^{1}H COSY

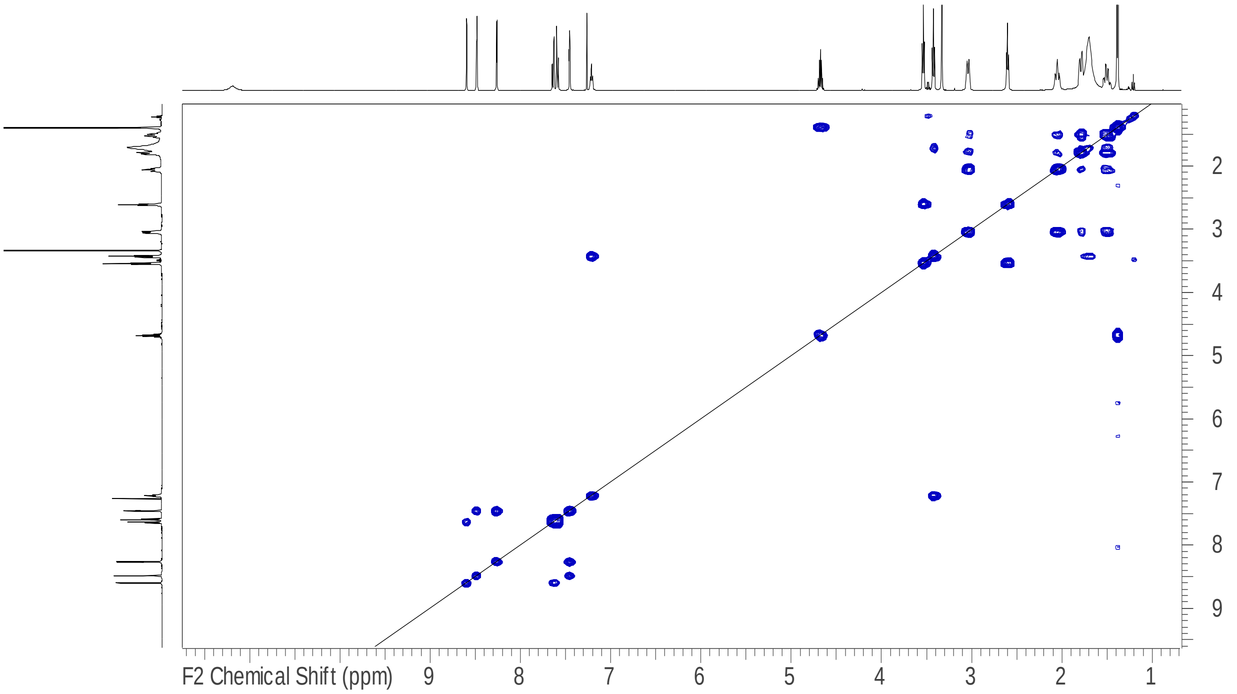

^{1}H-^{1}H NOESY

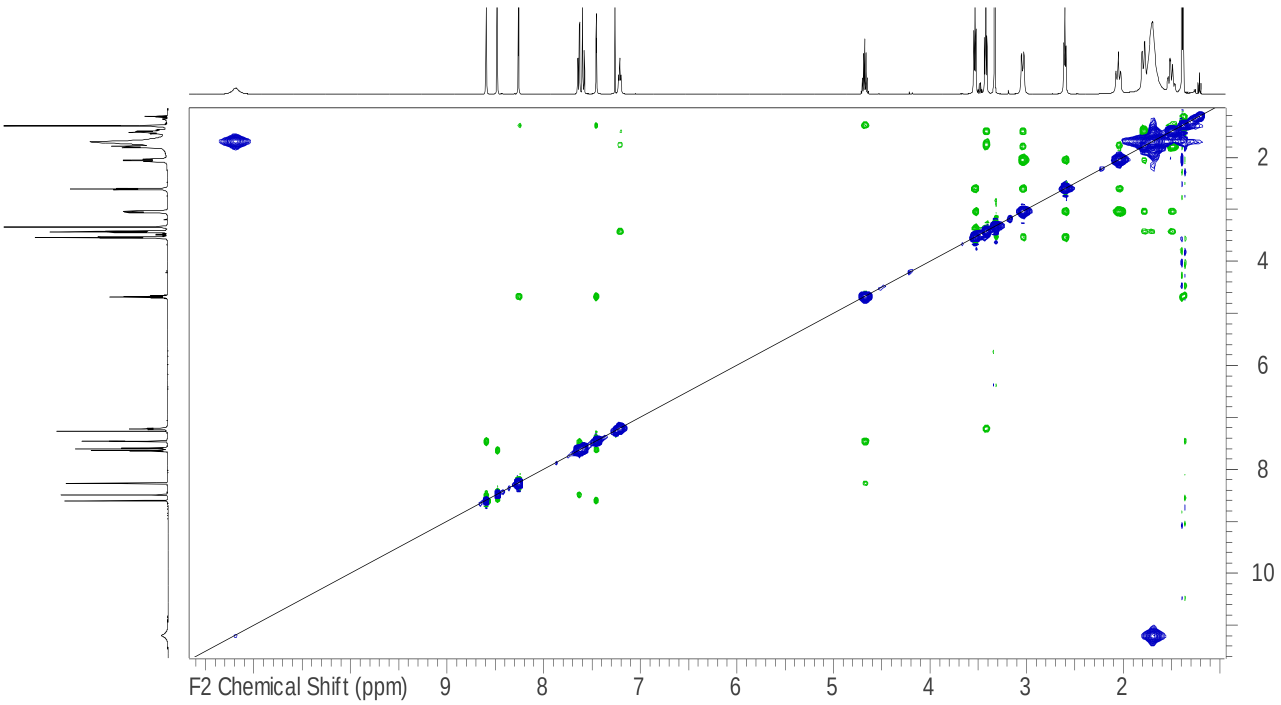

^{1}H-^{13}C **HSQC-DEPT**

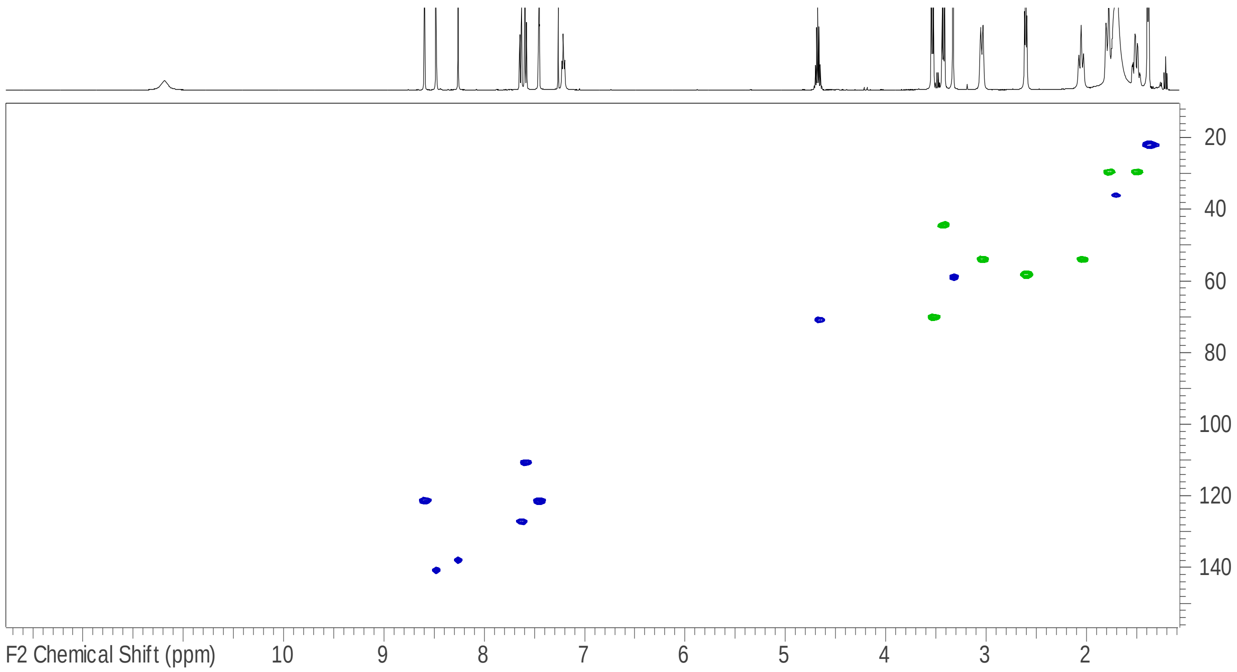

^{1}H-^{13}C **HMBC**

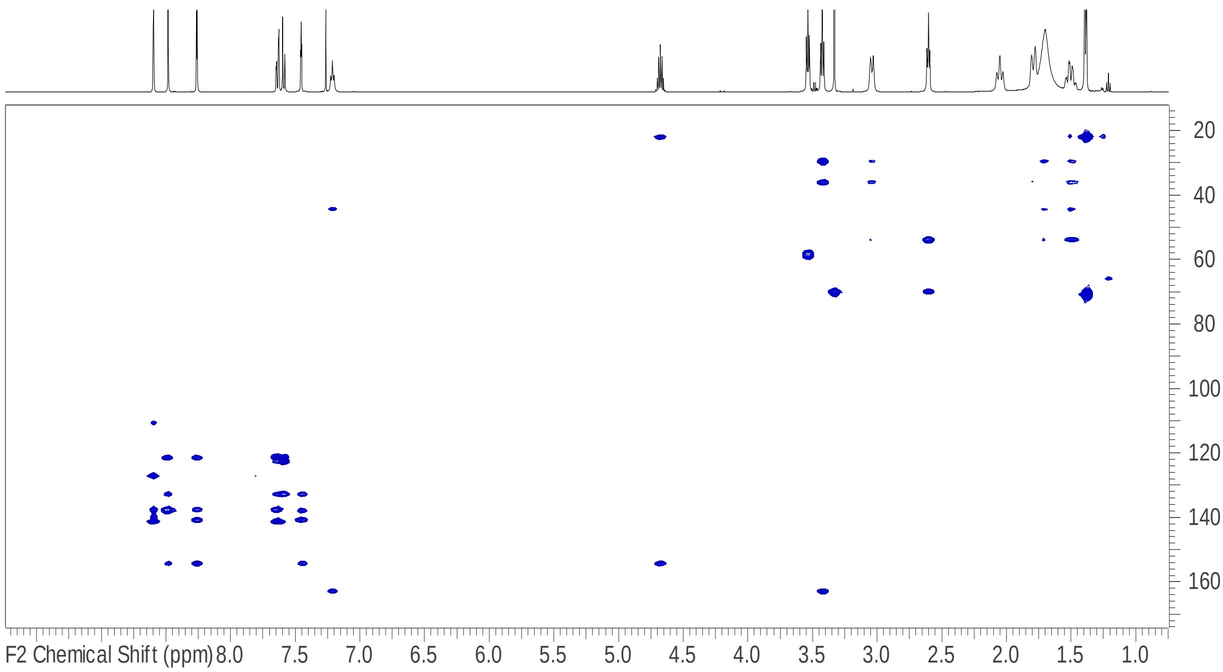

2D NMR spectra of compound 8

^{1}H-^{1}H COSY

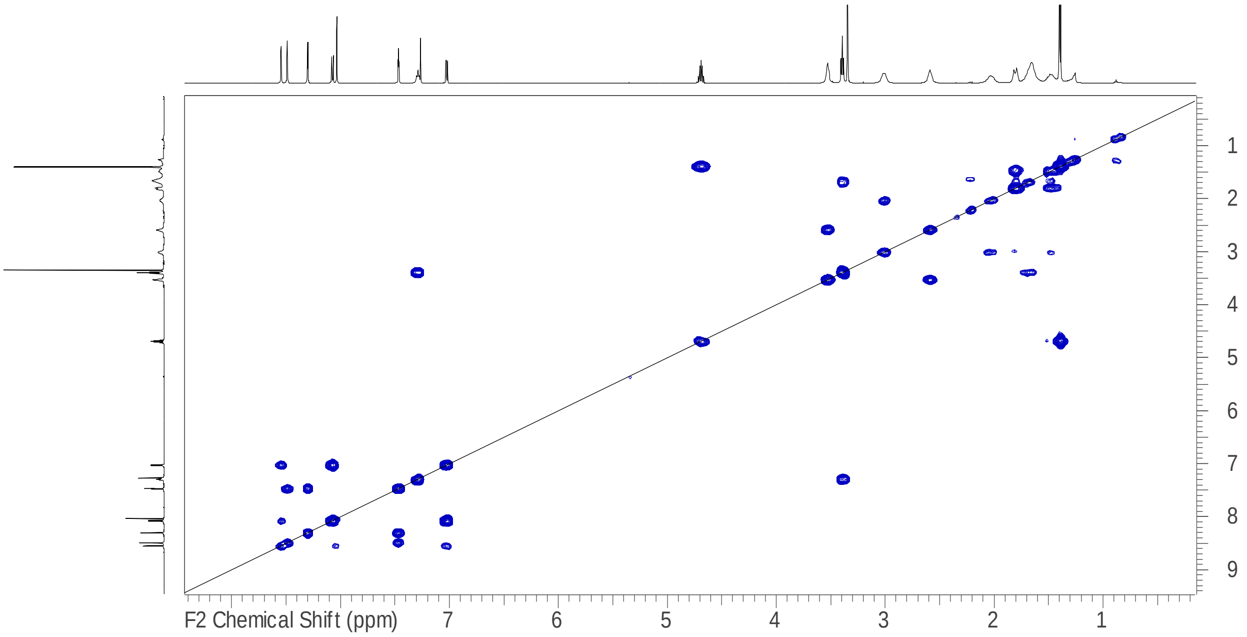

^{1}H-^{1}H NOESY

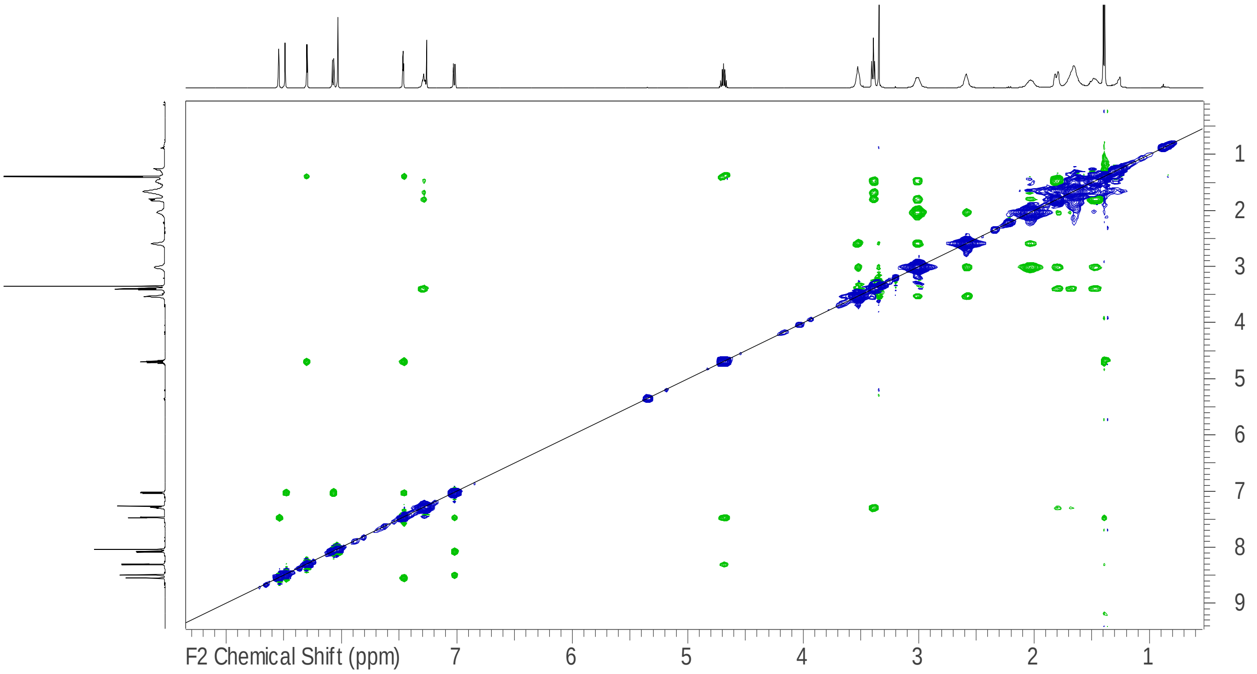

^{1}H-^{13}C HSQC-DEPT

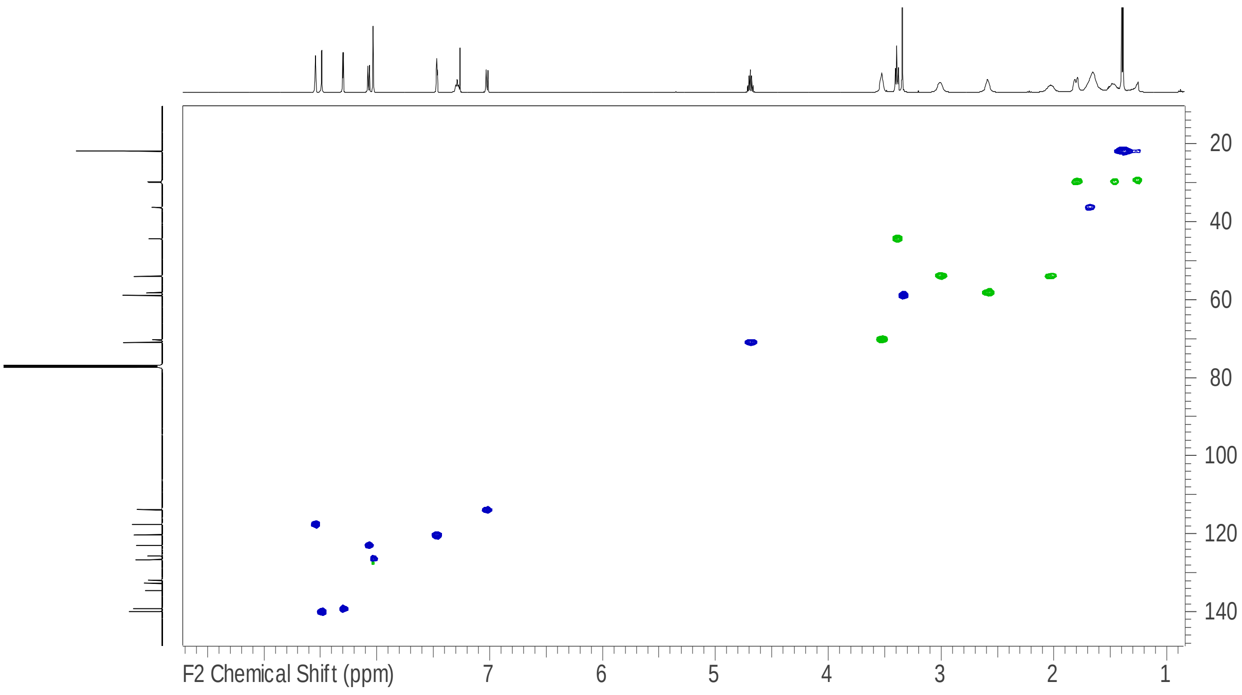

^{1}H-^{13}C HMBC

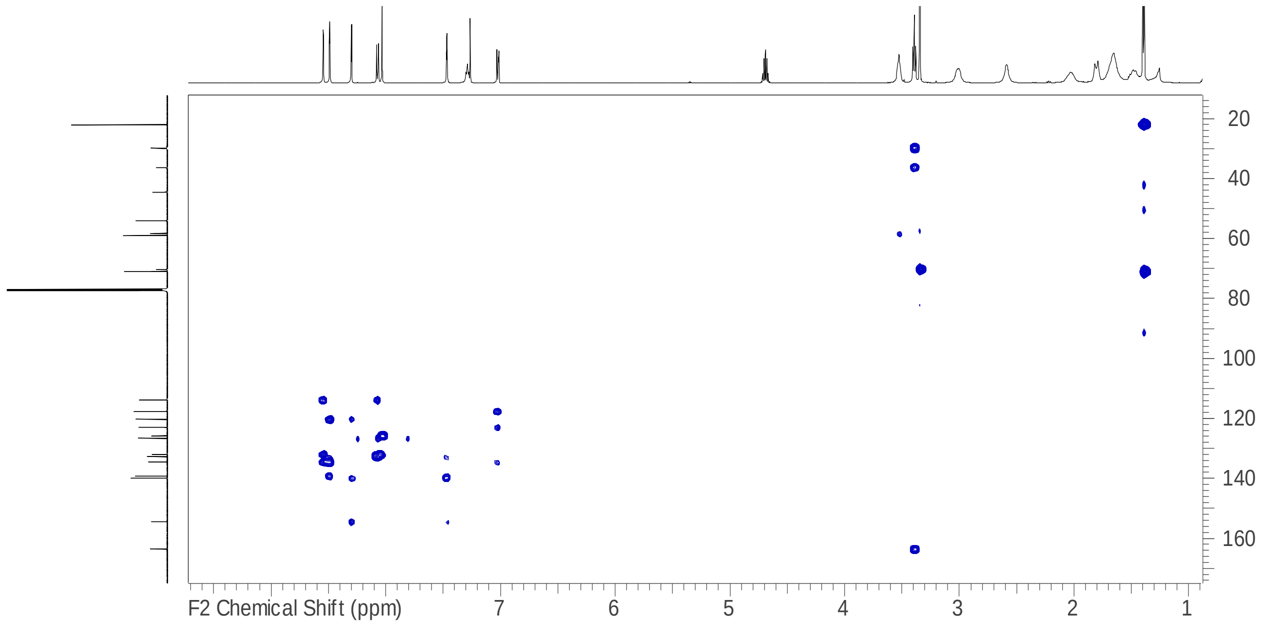

2D NMR spectra of compound 14

^{1}H-^{1}H COSY

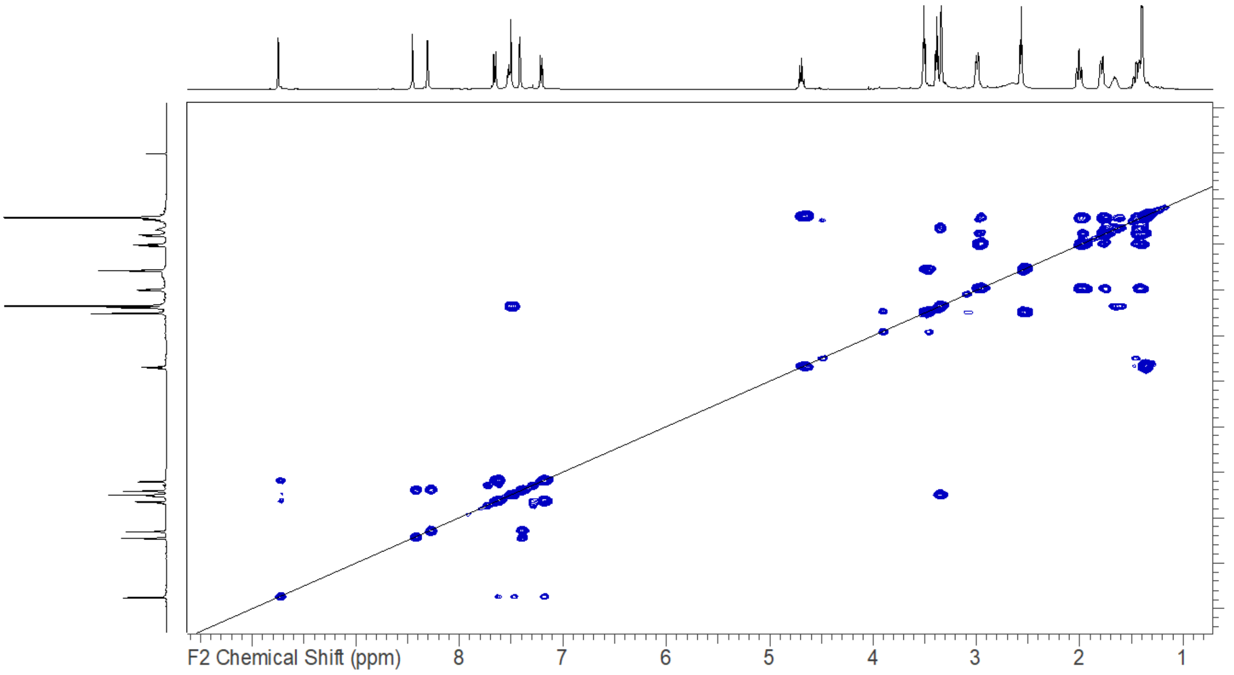

^{1}H-^{1}H NOESY

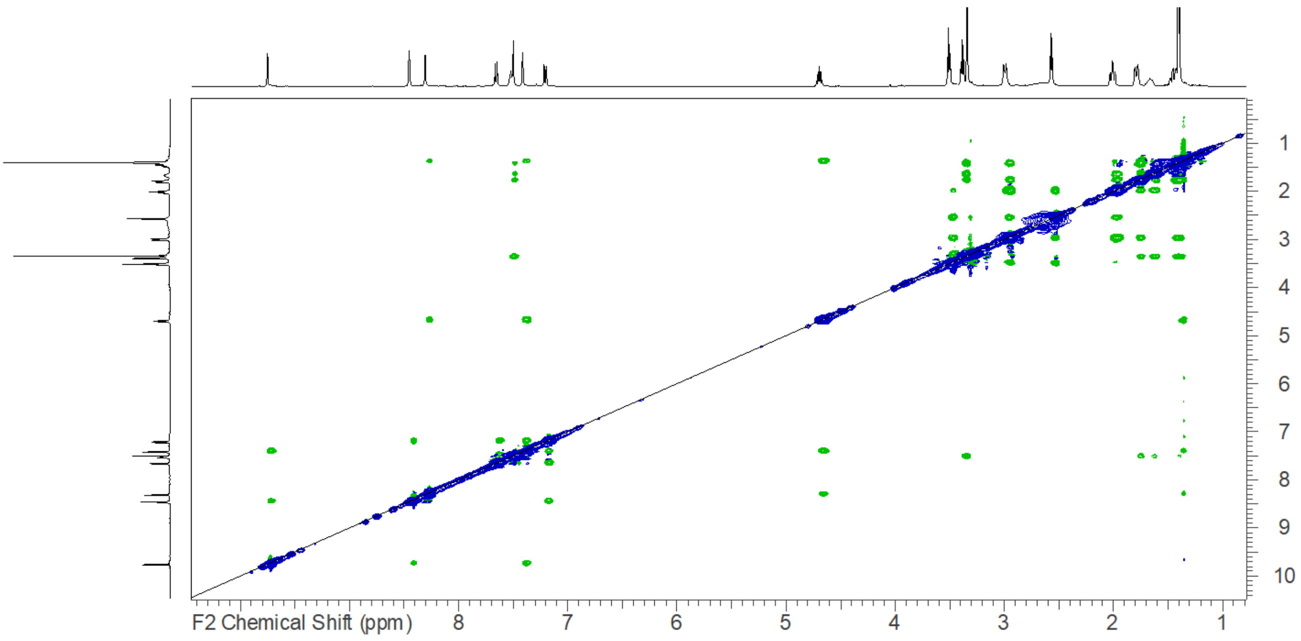

^{1}H-^{13}C HSQC-DEPT

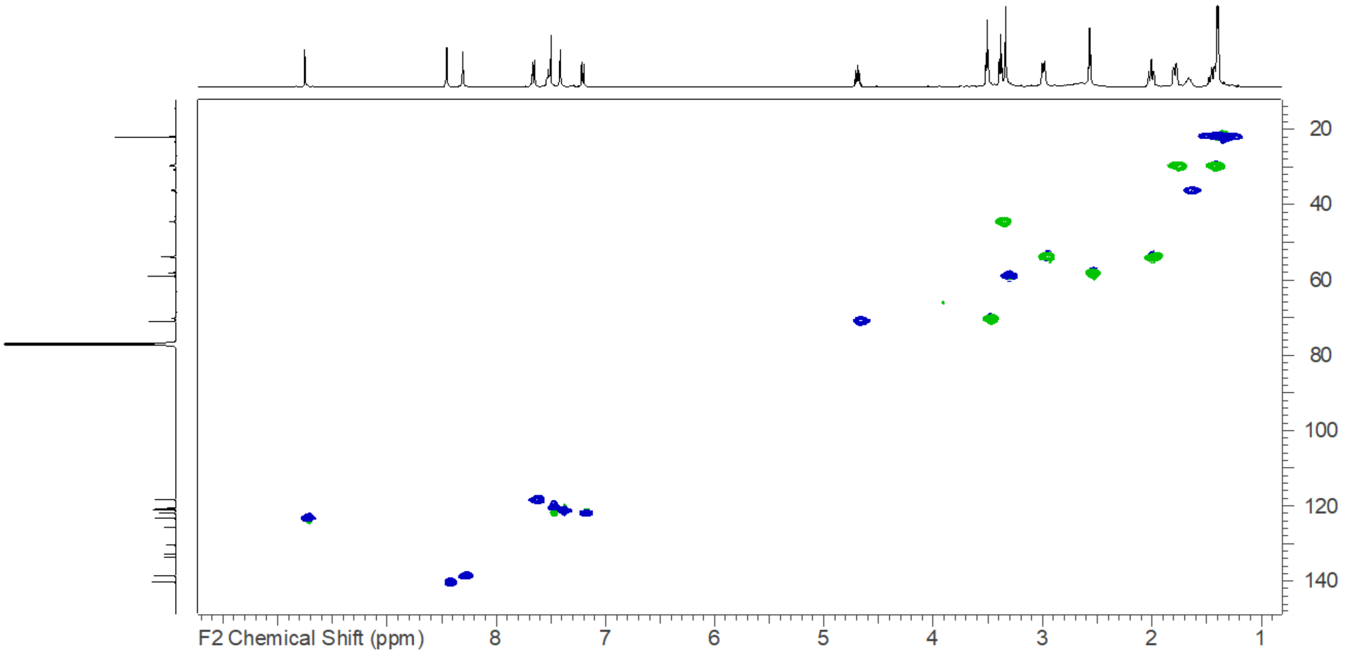

^{1}H-^{13}C HMBC

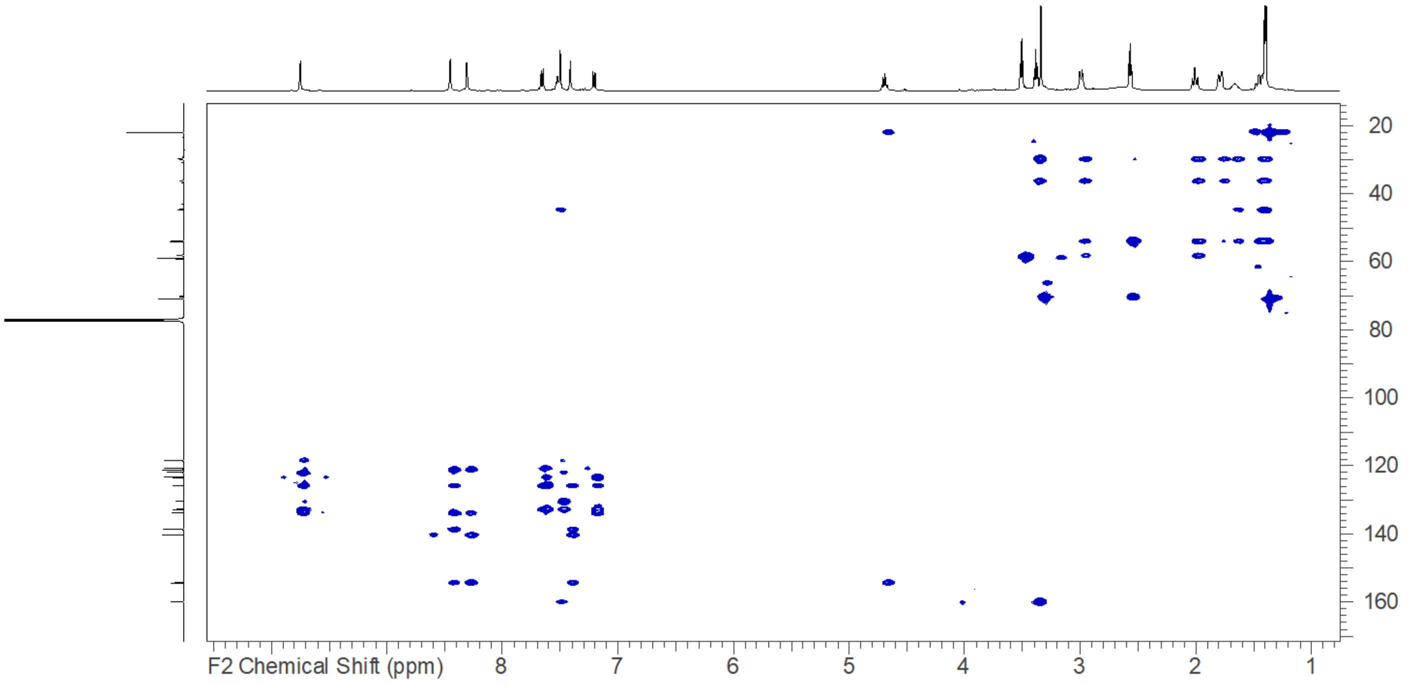

Supplement: Supplementary file 1 [file molecules-25-02163-s001.pdf]
